# Preparation of a chiral hyperbranched polymer based on cinchona alkaloids and investigation of its catalytic activity in asymmetric reactions

**DOI:** 10.55730/1300-0527.3677

**Published:** 2024-01-02

**Authors:** Rafiqul ISLAM, Mohammad Shahid ULLAH, Md. Abdus SALAM, Shinichi ITSUNO

**Affiliations:** 1 Department of Applied Chemistry and Life Science, Faculty of Engineering, Toyohashi University of Technology, Toyohashi, Aichi, Japan; 2 Department of Chemistry, Faculty of Natural Science and Mathematics, University of Houston, Houston, Texas, USA; 3 Department of Arts and Sciences, Faculty of Engineering, Ahsanullah University of Science and Technology, Dhaka, Bangladesh; 4 Department of Chemistry, Faculty of Science, University of Dhaka, Dhaka, Bangladesh; 5 Department of Applied Chemistry and Life Science, Toyohashi University of Technology, Toyohashi, Aichi, Japan; 5 Gifu College, National Institute of Technology, Kamimakuwa, Motosu, Gifu, Japan

**Keywords:** Hyperbranched polymers, sulfonamide, polymeric chiral organocatalyst, Michael addition reaction

## Abstract

Cinchona alkaloid-derived sulfonamides and ester dimers containing chiral hyperbranched polymers have been successfully synthesized and applied as catalysts in asymmetric reactions. Several hyperbranched polymers derived from cinchona alkaloids, incorporating sulfonamides and esters, were synthesized through Mizoroki–Heck coupling polymerization. These polymers were subsequently applied in enantioselective Michael addition reactions. As the prepared polymers are not soluble in frequently used organic solvents, they act as efficient catalysts in the enantioselective reaction of β-ketoesters to nitroolefins, achieving up to 99% enantioselectivity with good yields. The insoluble property allows them to better satisfy “green chemistry” requirements and be used several times without losing the enantioselectivity.

## 1. Introduction

Cinchona alkaloids are obtained from members of the plant family Rubiaceae, being derived from the bark of various species of cinchona trees [1]. Cinchona alkaloids are chemical substances with a vivid past and there are many instances of cinchona alkaloids being used today as chiral resolving agents [2–5]. The primary use of cinchona alkaloids in chemistry is for expediting various enantioselective transformations in both homogeneous and heterogeneous catalytic systems. Bredig and Fiske documented the first asymmetric reaction utilizing a cinchona base as early as 1912 [6,7]. The use of cinchona derivatives in asymmetric catalysis has grown dramatically since the publication of many ground-breaking studies. It is now understood that cinchona alkaloids and their derivatives are among the most prominent organic chirality inducers, activating nearly all chemical processes in a highly stereoselective manner. Their chiral induction and discrimination mechanisms were explained by structural analysis of cinchona alkaloids utilizing spectroscopic and computational techniques [8,9]. The main reason for the widespread use of cinchona alkaloids by numerous researchers [10,11] in various reactions, including hetero-[2+2] cycloadditions [12–14], phase-transfer catalyzed epoxidation [15–18], alkylation [19], conjugate additions [20,21], and phosphonylation reactions of aldehydes [22,23], was to obtain chiral catalysts with the use of these compounds in the 1970s and 1980s. Cinchona alkaloids have a variety of functions that are essential for producing chirality in asymmetric products, either on their own or in chemically altered forms [24], because they contain both acidic and basic sites that behave as dual-function chiral organocatalysts. A nucleophile and an electrophile can both be activated and oriented by the hydroxyl moiety and tertiary amine, respectively [25]. Cinchona alkaloids and their analogs are able to serve as catalysts that are chiral in four distinct types of transformations, including the formation of carbon-carbon bonds, carbon-oxygen bonds, and carbon-hydrogen heteroatom bonds, as well as additional processes including desymmetrization and hydrogenation. Bifunctional chiral catalysts, which can concurrently interact with and activate both reacting sites, are reliable and efficient materials for the stereoselective production of significant asymmetric molecules. Sulfonamides, which can be produced from cinchona alkaloids, are among the most important catalysts. In contrast to the tertiary nitrogen of quinuclidine, which in cinchona alkaloids may function as both a base and a hydrogen-bond acceptor, the acidic NH component of sulfonamide is capable of functioning as a hydrogen-bond donor. Since cinchona alkaloid-derived sulfonamides have both acidic and basic sites, they have the unusual ability of keeping a substrate in a certain orientation, creating a chiral environment [26]. Additionally, C9 ester derivatives of cinchona alkaloids with free OH [27], quinuclidine nitrogen [28–30], and a methoxy group adjacent to the C6’ position of the quinoline molecule have been extensively studied and effectively used in numerous asymmetric processes [31–33]. In addition to alternative varieties like bifunctional cinchona alkaloid derivatives, natural cinchona alkaloids are commonly utilized as a flexible source for organocatalysts in the field of catalytic enantioselective chemical synthesis [34,35]. Together with cinchona alkaloids with 6’-OH groups [36], dual-function cinchona alkaloid catalysts have also been identified, such as cinchona alkaloids with thiourea moiety [37] and cinchona alkaloids with 9-squaramide [38]. Sulfonamide catalysts based on cinchona alkaloids have been used to carry out asymmetric Michael-type reactions successfully. For instance, according to Luo et al., the asymmetric Michael reaction of 1,3-dicarbonyl compounds with nitrostyrene demonstrated good catalytic activity for quinidine-derived sulfonamide [39]. According to research by Itsuno and colleagues, the Michael addition reaction between ketoester and nitrostyrene exhibited greater stereoselectivity when cinchonidine sulfonamides served as bifunctional chiral organocatalysts [40].

Polymeric chiral organocatalysts are now used to great effect in the production of diverse chiral building blocks. A chiral organocatalyst (such as cinchona squaramides, sulfonamides, quaternary ammonium salt, cinchona ester, etc.) can be incorporated to produce a polymer that can be used as a chiral polymeric organocatalyst in many asymmetric reactions. Chiral polymers can include helical polymers, side-chain chiral polymers, main-chain chiral polymers, chiral ligands with dendritic molecules, and polymers with hyperbranched chirality. Polymeric chiral organocatalysts have attracted considerable interest in the chemical synthesis of molecules that are optically active due to their ease of removal from reaction mixtures and their capacity for multiple reuse. The design of chiral polymeric catalysts for hyperbranched chiral polymer organocatalysts is the primary focus of this work. Chiral catalysts were produced by copolymerizing a variety of chiral catalytic monomers with achiral monomers. A chiral catalyst was added to the main structure of the polymer during polymer immobilization. In recent years, significant advancements have been made in chiral main-chain polymeric catalyst synthesis processes. In addition, several polymer-immobilized catalysts have greater enantioselectivities compared to the corresponding catalysts with low molecular weight [41]. Different kinds of synthetic polymers, both organic and inorganic, have been employed as supports for chiral catalysts, and it has been documented which polymer network is best for each reaction [27]. As a substrate for chiral catalysts, various polymers have been used, such as cross-linked, branching, dendritic, and linear polymers. A functional polymer with a chiral ligand can be polymerized to create a polymer-support chiral organocatalyst, and different monomers can be utilized depending on the type of polymerization. Extremely branched three-dimensional macromolecules are known as hyperbranched polymers (HBPs) [42]. Due to their advantageous physical characteristics compared to their linear analogs, such as lower inherent viscosity, a lower glass transition temperature, and a larger number of terminal groups, HBPs attract significant attention [42–47]. They are appropriate for a variety of uses, serving as lubricants, coatings, medication delivery systems, and catalysts [48–52]. Furthermore, HBPs are relatively simple to manufacture in single-step polymerization using single-monomer or double-monomer methodology [53]. Our research team has already established that the Mizoroki–Heck coupling process is reliable in forming C-C bonds to produce chiral polymers from cinchona alkaloid derivatives [31,54,55], and we are now concentrating on this coupling reaction in the present study to synthesize HBPs. The olefinic double bond of the sulfonamide dimer generated from cinchona alkaloids, the cinchona ester dimer, and the halide of the trifunctionalized aromatic iodide were combined in the Mizoroki–Heck process to create chiral HBPs. With the asymmetric Michael addition reaction, we employed these HBPs as chiral polymeric organocatalysts.

## 2. Results and discussion

### 2.1 Synthesis of cinchona-derived sulfonamide and ester dimers and their corresponding chiral hyperbranched polymers

**Scheme 1:**
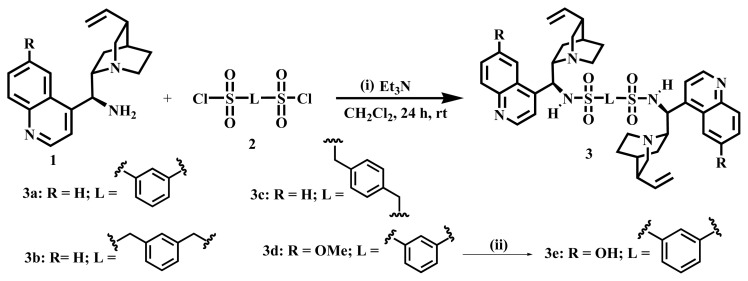
i) Synthesis of cinchona-based sulfonamide dimers. ii) Demethylation of **3d** dimer by 1 M BBr_3_, dry CH_2_Cl_2_, Ar gas, −78 °C to rt, 48 h.

In this study, we focused on designing HBPs based on cinchona sulfonamide and cinchona ester dimers. These HBPs contain rigid catalytic centers that are substantially more numerous, which may create a favorable microenvironment at the catalytic sites and enable the systematic manipulation of their catalytic characteristics. We have synthesized various polymers of chiral organocatalysts by ion-exchange polymerization, etherification polymerization, neutralization polymerization, and quaternization polymerization. Sulfonyl chloride is highly reactive towards amine, giving sulfonamide derivatives even in mild reaction conditions. Thus, sulfonamide dimers **3**, excluding **3e**, were designed and synthesized by the combination of C-9 aminated cinchona alkaloids **1** [3(*R*),4(*S*),8(*S*),9(*S*)] and disulfonyl chloride **2** (Scheme 1) at room temperature. A reaction time of 24 h with an excess amount of **1** (roughly double the amount of **2**) resulted in good yield. C-9 aminated cinchona alkaloids **1** were synthesized from cinchona alkaloids **4** [3(*R*),4(*S*),8(*S*),9(*R*)] having the C-9 hydroxyl group using the reported procedure [56,57].

**Scheme 2:**
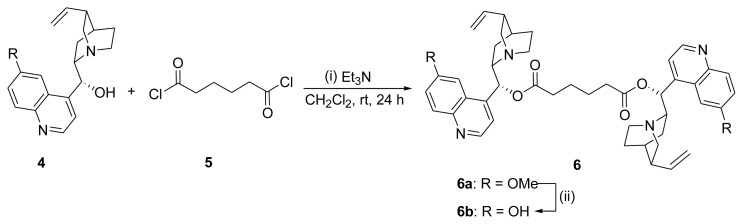
i) Synthesis of ester dimers of cinchona. ii) Demethylation of **6a** dimer by 1 M BBr_3_, dry CH_2_Cl_2_, Ar gas, −78 °C to rt, 48 h.

C-6’ OH-carrying dimer **3e** was procured by demethylation of **3d** using BBr_3_ (Scheme 1) at −78 °C for 2 days. On the other hand, dimeric ester **6a** was obtained from C-9 hydroxyl cinchona alkaloids **4** and hexa-acid chloride **5**. Cinchona ester dimer **6b** was obtained from **6a** as **3e** was prepared by demethylation (Scheme 2).

**Scheme 3:**
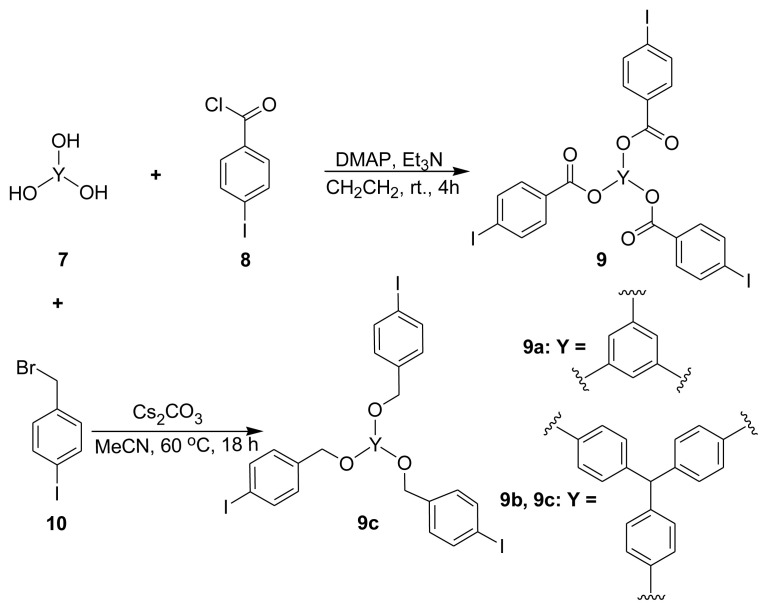
Different synthetic routes of trifunctional aromatic iodides.

**Scheme 4:**
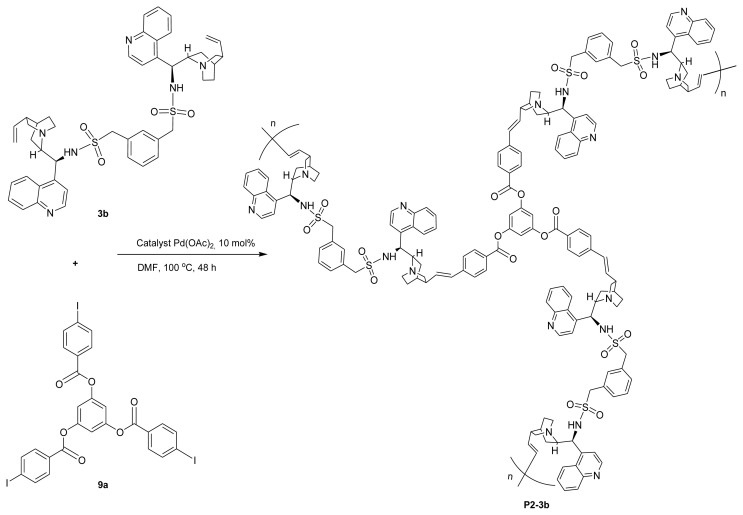
Synthesis of chiral HBP **P2-3b**.

Novel chiral HBPs with cinchona-based sulfonamide and ester dimers were designed by accumulating bifunctional dimers and trifunctional aromatic halides (**9**). The two C3-vinyl groups in the structure of cinchona dimers make it possible to carry out the polymerization process with aromatic iodides using a two-component approach, and the Mizoroki–Heck coupling reaction is the most effective reaction among the numerous reactions that can proceed to a C-C bond with a vinylic double bond [54,55]. In order to produce the polymers, we therefore used the Mizoroki–Heck reaction between aromatic triiodides and divinylic compounds. Trifunctional aromatic iodide compounds **9a** and **9b** were respectively prepared from trihydroxybenzene and tris-phenol with iodobenzoylchloride **8** at room temperature (Scheme 3) [58,59]. Tris-phenol and iodobenzylbromide **10** were used to produce another class of trifunctional compounds (**9c**) with three iodophenyl groups (Scheme 3) [58]. Repeated Mizoroki–Heck reactions take place in the presence of catalyst Pd(OAc)_2_ when triiodo aromatic compounds **9** are combined with cinchona dimers **3** or **6**, and the resulting chiral HBPs (Scheme 4) are produced with high yield (up to 93%; Table 1, entry 6). One reaction route is shown in Scheme 4.

The reaction mixture was precipitated in ether after polymerization and then washed with ether and water to yield the polymer powder. The desired polymers of entries 1–5 in Table 1 were prepared by Mizoroki–Heck polymerization using cinchona alkaloid-based sulfonamide dimers **3** and entries 8 and 9 resulted from cinchona ester dimers **6** with triiodide **9a**, while entries 6 and 7 were procured from different types of trifunctional aromatic iodides (**9b** and **9c**) with sulfonamide dimers **3b**. The HBPs that we obtained were soluble in DMF and DMSO, except **P6-3b** and **P7-3b**, which dissolved minimally. All polymers were slightly dissolved in other prevalently used organic solvents, such as dichloromethane, methanol, diethyl ether, ethyl acetate, THF, and hexane, as well as acetone. The outcomes of the Mizoroki–Heck polymerization of the aromatic triiodides and cinchona dimers are shown in Table 1. In all cases, chiral HBPs with higher molecular weight of over 10,000 were found. However, we could not obtain the molecular weight for polymers **P6-3b** and **P7-3b** due to their poor solubility in DMF.

### 2.2 Catalytic activity of cinchona alkaloid-derived dimers and hyperbranched polymers

**Scheme 5:**
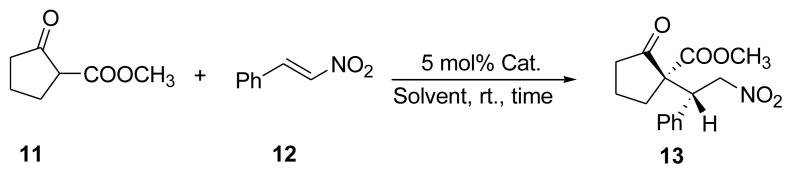
Asymmetric Michael addition of β-ketoesters (**11**) with *trans*-β-nitrostyrene (**12**).

We selected the asymmetric Michael addition of methyl 2-oxocyclopentanecarboxylate **11** to *trans*-β-nitrostyrene **12** as the model reaction (Scheme 5) to examine the catalytic function of the cinchona-based chiral HBPs. Initially, we examined dimeric low-molecular-weight catalysts in the enantioselective Michael addition reaction in CH_2_Cl_2_ at room temperature and the reaction proceeded smoothly, generating excellent enantioselectivity of up to 99% with good yields (up to 96%), excluding **6a**, which demonstrated only 44% ee (Table 2, entry 6). Table 2 provides a summary of the results of the asymmetric Michael reaction of **11** and **12** using low-molecular-weight dimeric catalysts. We were encouraged by those results to use the corresponding sulfonamide polymers as catalysts while applying the same procedure. The HBPs of the respective dimers were then synthesized as polymeric organocatalysts and employed for the same reaction. In the first instance, we investigated HBP catalyst **P1-3a**.

Although it was insoluble in CH_2_Cl_2_ and gave a heterogeneous mixture, the asymmetric Michael addition of *trans*-nitrostyrene **12** and methyl 2-oxocyclopentanecarboxylate **11** progressed without any cumbersome steps at room temperature to provide the corresponding asymmetric product with up to 99% ee at 96% yield. However, a longer reaction time was needed due to the heterogeneous system for polymeric catalysts. The results were similar compared to those for a previously reported cinchona-based sulfonamide main-chain type linear polymer [59]. In this case, half catalyst loading (5 mol.%) was required compared to the linear polymers.

Shorter reaction time was needed when **P2-3b** with a more flexible structure than **P1-3a** was used as the catalyst. Chiral product **13** was obtained with nearly perfect enantioselectivity of the major diastereomer (over 99%) within 24 h (Table 3, entry 2). Though it had better enantioselectivity compared to the corresponding dimer, its diastereoselectivity was somewhat diminished. Acceptable performance was achieved with chiral HBPs in the particular asymmetric reaction, which might be attributed to creating an appropriate microenvironment in the chiral polymer network. Other polymers also demonstrated good enantioselectivity (94% to 99%), excluding the results obtained using **P8-6a** (Table 3, entry 8). It was derived from quinine ester dimer **6a** with a C6’ methoxyl group, which gave lower enantioselectivity for the selected model Michael reaction due to the lack of acidic protons. Poor enantioselectivity was also displayed by dimeric catalyst **6a**. The enantioselective Michael addition reaction proceeded under the same conditions when chiral HBP **P9-6b** with C6’-OH was used as a catalyst, yielding **13** with significantly better enantioselectivity (99% ee; Table 3, entry 9). Compared to the results obtained using corresponding dimer catalyst **6b**, the **P9-6b** catalyst required more time because of the heterogeneous conditions. Using trifunctional aromatic compounds **9b** and **9c** instead of **9a**, lower enantioselectivity and yield were obtained with longer reaction times for HBPs **P6-3b** and **P7-3b** (Table 3, entries 6 and 7) compared to **P2-3b** (Table 3, entry 2). We investigated the influence of the solvents on the catalytic activity by using HBP **P2-3b**. The results of the Michael addition reaction for **P2-3b** are presented in Table 4 for diverse solvents. The reactions were highly enantioselective, achieving values above 95% ee for all selected solvents with good yields. However, in the case of ethyl acetate, only 27% yield was obtained with 97% ee (Table 4, entry 4). Acetonitrile, THF, and acetone gave somewhat lower yields (Table 4, entries 2, 6, and 7) compared to dichloromethane but maintained good enantioselectivity. The most effective solvent for this model Michael reaction was CH_2_Cl_2_, with over 99% ee and 84% yield, as determined after investigating the impact of the solvent (Table 4, entry 1).

**Scheme 6:**
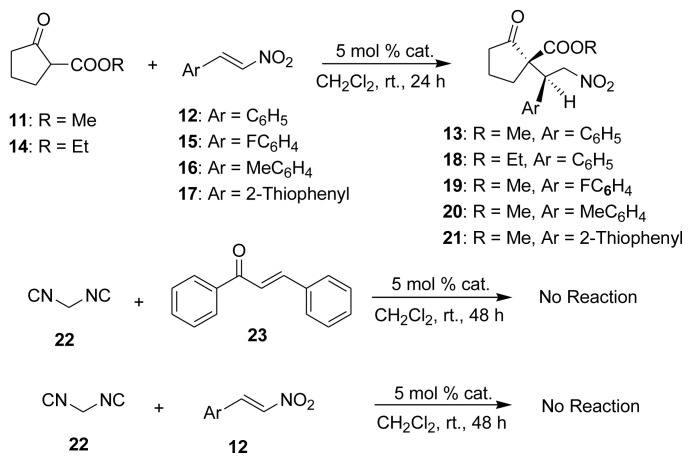
Michael addition reaction of various Michael donors and acceptors using polymer **P2-3b** as catalyst.

We subsequently applied chiral HBP **P2-3b** to monitor the asymmetric Michael addition reaction by changing the Michael acceptor substituents as well as the Michael donors (Scheme 6), and the results are summarized in Table 5. Higher enantioselectivity was observed upon using methyl 2-oxocyclopentanecarboxylate **11** and ethyl 2-oxocyclopentanecarboxylate **14** as Michael donors for all reactions (Table 5, entries 1, 3, and 4).

4-Fluoro-*trans*-nitrostyrene **15** and methyl 2-oxocyclopentanecarboxylate **11** interacted with **P2-3b** to produce Michael adducts **19** with just 72% ee. However, chiral catalyst **P2-3b** was ineffective in catalyzing the reaction between malononitrile **22** with chalcone **23** and *trans*-β-nitrostyrene **12** to respectively give a chiral product at room temperature. The polymeric catalysts utilized in the asymmetric reaction were easily separated and recovered from the reaction mixture by normal filtration since the chiral HBPs were insoluble in organic solvents frequently used to give suspensions. The recovered HBPs were applied in the same asymmetric reaction multiple times. To confirm the authenticity of chiral HBP **P2-3b** used as a model catalyst in asymmetric reactions in dichloromethane at room temperature, this polymer was reused in up to five cycles to monitor the catalytic activity. The yield in entry 3 is higher compared to entry 2 due to the increase of the reaction time from 24 to 30 h. Other recyclability results are summarized in Table 6. Although catalyst **P2-3b** maintained its original enantioselectivity and diastereoselectivity upon being reused, the yield decreased to some extent.

## 3. Experimental

### 3.1. Synthesis of cinchona-derived sulfonamide and ester dimers

#### 3.1.1. Synthesis of compound 3b

Cinchonidine amine **1** (1099.0 mg, 3.7456 mmol; 2 equiv. or double amount of **2**), α,α′-m-xylene sulfonyl chloride **2** (545.0 mg, 1.7977 mmol), triethyl amine (522 μL, 3.7456 mmol), and a magnetic stirring bar were added to a 20-mL volumetric flask. Subsequently, 10.0 mL of dry CH_2_Cl_2_ was added to the mixture and it was kept at room temperature while being stirred. Reaction progress was observed by TLC. The crude compound was purified using silica gel (100–200 mesh) column chromatography with a CH_2_Cl_2_:MeOH = 9:1 eluent after 24 h, yielding target component **3b** in 48% yield as a white solid, mp: 151–153 °C. ${\left[\alpha \right]}_{D}^{26.4}=-7.53$ (*c* 0.19 g/dL in DMF). ^1^H NMR (400 MHz, CDCl_3_, 25 °C) δ 8.95–8.92 (m, 2H), 8.23–8.28 (m, 2H), 8.10–8.12 (m, 2H), 7.68–7.45 (m, 2H), 7.50–7.63 (m, 4H), 7.32 (d, *J* = 4.8, 1H), 7.02 (s, 1H), 6.83–6.92 (m, 2H), 6.59–6.92 (m, 2H), 6.59 (d, *J* = 11.2, 1H), 5.54–5.22 (m, 2H), 4.85–4.99 (m, 4H), 4.58 (d, *J* = 8.8, 1H), 3.58–3.77 (m, 4H), 3.14–3.24 (m, 4H), 2.86–3.02 (m, 2H), 2.68–2.77 (m, 4H), 2.28 (br, 2H), 1.57–1.69 (m, 6H), 1.25–1.31 (m, 2H), 0.74–0.92 (m, 2H) ppm. ^13^C NMR (100 MHz, CDCl_3_, 25 °C) δ 150.3, 148.5, 145.9, 141.2, 132.3, 130.8, 130.4, 129.7, 128.9, 127.4, 124.9, 122.8, 120.0, 114.8, 60.7, 59.8, 55.5, 52.7, 40.4, 39.5, 27.6, 25.5 ppm. IR (KBr) *v* 3213, 2938, 2865, 1708, 1590, 1509, 1455, 1424, 1319, 1222, 1149, 1128, 988, 764 cm^−1^. HRMS (ESI) calcd for C_46_H_52_N_6_O_4_S_2_ [M+Na]^+^: 817.02, found: 817.3606.

Other cinchona-derived sulfonamide and ester dimers (**3c**, **3d**, and **3e**) were prepared from different cinchona derivatives and sulfonyl chloride using the same process as reported in the Supporting Information.

#### 3.1.2. Synthesis of trifunctional aromatic triiodides

##### 3.1.2.1. Synthesis of compound 9b

CH_2_Cl_2_ (50 mL) was used in mixing 4,4’,4”-trihydroxyphenylmethane **7** (1.461 g, 5.0 mmol), 4-iodobenzoylchloride **8** (4.132 g, 15.5 mmol), Et_3_N (2.2 mL, 15.5 mmol), and DMAP (0.20 g). At room temperature, the resulting reaction mixture was stirred constantly for 4 h. The layers were then separated after the addition of water. Additional CH_2_Cl_2_ was used to extract the aqueous phase, and the mixed organic layer was washed with brine, 10% aq. HCl solution, and 5% aq. NaOH solution before being dried over anhydrous MgSO_4_. The crude product was obtained after filtration and solvent removal, and the chemical was then refined using silica gel column chromatography with Hex:EtOAc = 9:1 to produce white solid **9b** with 48% yield, mp: 104–107 °C. ^1^H NMR (CDCl_3_, 400 MHz, 25 °C) δ 7.86–7.91 (m, 12H), 7.15–7.21 (m, 12H), 5.63 (s, 1H) ppm. ^13^C NMR (100 MHz, CDCl_3_, 25 °C) δ 164.6, 149.2, 141.0, 137.9, 131.4, 130.4, 128.9, 121.5, 101.6, 55.0 ppm.

##### 3.1.2.2. Synthesis of compound 9c

In a 30-mL flask, 15.0 mL of CH_3_CN was employed to dissolve 4,4’,4”-trihydroxyphenylmethane 7 (292.34 mg, 1.0 mmol) and 4-iodobenzylbromide **10** (979.5 mg, 3.3 mmol). Cesium carbonate (Cs_2_CO_3_, 1075.2 mg, 3.3 mmol) was then added to the mixture. Under an Ar environment, the mixture was stirred at 60 °C for 18 h. After that, 60 mL of CH_2_Cl_2_ was combined with the reaction mixture. A yellow solid product was formed and separated by filtering and evaporating the organic solution under reduced pressure after it had been cleaned with water (2/30) and brine (2/30). The organic solution was also dried over anhydrous magnesium sulfate. Compound **9c** was obtained with 31% yield as a white solid after the crude product was refined using silica gel (100–200 mesh) column chromatography with Hex:DCM = 1:1. R_f_: 0.42 (DCM/Hex = 5.0/5.0). Other experimental data are provided in the Supporting Information.

### 3.2. Synthesis of HBPs by Mizoroki–Heck polymerization reaction

For the synthesis of polymer **P1-3a**, compounds **3a** (100.0 mg, 0.12674 mmol) and **9a** (104.0 mg, 0.12674 mmol) were combined in a 30-mL flask with triethyl amine (double the amount; 35 μL, 0.2535 mmol). Palladium acetate (10 mol.%) and DMF solvent (3 mL) were added, and the mixture was stirred at 100 °C for 48 h. NMR was used to observe the course of the process of the reaction. The solvent was then evaporated and washed with a suitable solvent, diethyl ether, and finally water. The desired polymeric compounds were then dried again in a vacuum oven to produce the small compound **P1-3a** as a brown solid in 79% of the cases. ${\left[\alpha \right]}_{D}^{24.4}=+39.40$ (*c* 0.05 g/dL in DMF). ^1^H NMR (400 MHz, DMSO-*d*_6_, 25 °C) δ 8.68, 7.27–8.22 (aromatic H), 6.37–6.63 (vinylic H), 5.10, 0.61–2.92 (quinuclidine H) ppm. IR (KBr) *v* 3178, 3067, 2942, 2865, 1733, 1652, 1604, 1509, 1458, 1327, 1257, 1177, 1069, 1004, 758, 683 cm^−1^. *M*_n_ (SEC) = 8.0 × 10^3^, *M*_w_/*M*_n_ = 1.65.

Using the same procedure described in the Supporting Information, additional optically active HBPs were synthesized from various sulfonamide and ester dimers derived from cinchona. Table 1 summarizes the relevant results.

### 3.3. General procedure for the asymmetric Michael addition reaction of β-ketoesters to nitroolefins using chiral sulfonamide polymers

*Trans*-nitrostyrene **12** (82.05 mg, 0.55 mmol) and methyl 2-oxocyclopentanecarboxylate **11** (63μL, 0.50 mmol) were taken in a reaction vessel with 2.5 mL of solvent. HBP catalyst was then poured into the mixture (5 mol.%). The reaction mixture was then stirred for a predetermined amount of time at room temperature. A rotary evaporator was used to evaporate the solvent once all **11** had been consumed as determined by TLC. To remove the utilized polymeric catalyst from the reaction mixture, the solution containing the asymmetric compound was collected by pipette after being washed with ether. In order to obtain the desired asymmetric compound, the solution was concentrated in vacuo and the compound was purified using column chromatography on silica gel (100–200 mesh) with hexane:EtOAc = 6.0:1.0 as the eluent to afford the title asymmetric compound as a colorless oil. ^1^H NMR (400 MHz, 25 °C, CDCl_3_) δ 7.29–7.23 (m, 5H), 5.14 (dd, *J* = 13.8 Hz, 3.8 Hz, 1H), 5.00 (dd, *J* = 13.8 Hz, 10.7 Hz, 1H), 4.08 (dd, *J* = 10.8 Hz, 3.8 Hz, 1H), 3.74 (s, 3H), 2.38–2.33 (m, 2H), 2.04–1.84.

The outcomes of further asymmetric Michael additions were achieved in similar ways, as shown in Tables 2–6 as well as in Scheme 5.

## 4. Conclusion

In this study, we successfully developed novel chiral HPBs using the Mizoroki–Heck polymerization method. These HPBs have a primary chain repeating unit made of a sulfonamide and ester structure based on cinchona. For chiral polymerization, two components were employed as the approach. Despite the fact that these chiral polymers are insoluble in commonly used organic solvents, they function as superb catalysts in the asymmetric Michael addition of ketoesters to nitroolefins, resulting in up to 99% enantioselectivity and good yield. Chiral HBP **P2-3b** shows an excellent level of enantioselectivity (>99% ee) with good yield as a low-molecular-weight catalyst. Their insoluble property allows these polymers to better satisfy “green chemistry” requirements and be used several times without losing enantioselectivity. These HBPs based on sulfonamide and the ester dimer of cinchona alkaloid were successfully applied in enantioselective synthesis.

## Supporting Information

### [Materials and General Considerations]

All solvents and reagents were brought from Sigma-Aldrich, Wako Pure Chemical Industries, Ltd., or Tokyo Chemical Industry (TCI) Co., Ltd. at the maximum available cleanness and were used as received. Pre-coated silica gel plates (Merck 5554, 60F254) was used for Thin-layer chromatography (TLC) to monitor various types of reactions progression. Column chromatography was conducted by using a silica gel column (Wakogel C-200, 100–200 mesh). Yanaco micro melting apparatus was used to record melting point and the average values of the analysed samples were taken. NMR spectra were recorded on JEOL JNM-ECS400 spectrometers and JEOL JNM-ECX500 spectrometers in CDCl3 or DMSO-d6 at room temperature operating at 400 MHz (1H), 500 MHz (1H) and 100 MHz (13C{1H}). For 1H NMR Tetramethylsilane (TMS) was used as an internal standard and chemical shifts were reported in parts-per-million (ppm). CDCl3 was used as standard for 13C NMR and the J values were reported in hertz. JEOL JIR-7000 Fourier transform (FT)-IR spectrometer was use to record IR spectra and reported in reciprocal centimeters (cm−1). High-resolution mass spectrometry (HRMS) electrospray ionization (ESI) spectra were recorded using Bruker micro TOF-Q II HRMS/MS instrument. High-performance liquid chromatography (HPLC) was run with a Jasco HPLC system constructed of a DG-980-50 three-line degasser, a HPLC pump (PU-980), a Jasco UV-975 UV detector for peak detection, and a column oven CO-2065 equipped with a chiral column (Chiralpak OD-H, Daicel) with hexane/2-propanol as the eluent at a flow rate of 1.0 mL/min at room temperature. Size-exclusion chromatography (SEC) was performed using a Tosoh HLC 8020 instrument with UV (254 nm) or refractive index detection. As a carrier solvent dimethylformamide (DMF) was used at a flow rate of 1.0 mL min-1 at 40 °C and two polystyrene gel columns of 10-μm bead size were used. The number average molecular weight (Mn) and molecular weight distribution (Mw/Mn) values were determined by using a calibration curve compared with polystyrene standards. The optical rotation was obtained by using a JASCO DIP-149 digital polarimeter using a 10-cm thermostatted microcell.

#### Synthesis of cinchona derived sulfonamide and ester dimers

##### Synthesis of compound 3b

Cinchonidine amine 1 (1099.0 mg, 3.7456 mmol; 2 equiv or little excess), α,α’-m-xylene sulfonyl chloride 2 (545.0 mg, 1.7977 mmol), triethyl amine (522 μL, 3.7456 mmol) and magnetic stir bar taken in a 20 mL volumetric flask. Then dry CH_2_Cl_2_ 10.0 mL added to the mixture and kept it at room temperature with stirring. The reaction progress was observe by TLC. After 24 hours CH_2_Cl_2_ was removed by rotary evaporator and then the crude compound was purified by silica gel (100–200 mesh) column chromatography using CH_2_Cl_2_: MeOH = 9:1 as an eluent to give the desired compound 3b in 48% yield as white solid. mp: 151–153 °C. ${\left[\alpha \right]}_{D}^{26.4}=-7.53$ (*c* 0.19 g/dL in DMF). ^1^NMR (400 MHz, CDCl_3_, 25 °C) δ 8.95–8.92 (m, 2H), 8.23–8.28 (m, 2H), 8.10–8.12 (m, 2H), 7.68–7.45 (m, 2H), 7.50–7.63 (m, 4H), 7.32 (d, J=4.8, 1H), 7.02 (s, 1H), 6.83–6.92 (m, 2H), 6.59–6.92 (m, 2H), 6.59 (d, J=11.2, 1H), 5.54–5.22 (m, 2H), 4.85–4.99 (m, 4H), 4.58 (d, J=8.8, 1H), 3.58–3.77 (m, 4H), 3.14–3.24 (m, 4H), 2.86–3.02 (m, 2H), 2.68–2.77 (m, 4H), 2.28 (br, 2H), 1.57–1.69 (m, 6H), 1.25–1.31 (m, 2H), 0.74–0.92 (m, 2H) ppm. ^13^C NMR (100 MHz, CDCl_3_, 25 °C) δ 150.3, 148.5, 145.9, 141.2, 132.3, 130.8, 130.4, 129.7, 128.9, 127.4, 124.9, 122.8, 120.0, 114.8, 60.7, 59.8, 55.5, 52.7, 40.4, 39.5, 27.6, 25.5 ppm. IR (KBr) *v* 3213, 2938, 2865, 1708, 1590, 1509, 1455, 1424, 1319, 1222, 1149, 1128, 988, 764 cm^−1^. HRMS (ESI) calcd for C_46_H_52_N_6_O_4_S_2_ [M+Na]^+^ : 817.02 found: 817.3606.

###### Supplementary Data

Figure S1 ^1^H NMR of dimer 3b in CDCl_3_

Figure S2 ^13^C NMR of dimer 3b in CDCl_3_

Figure S3 IR spectra of 3b

Figure S4 ^1^H NMR of dimer 3c in CDCl_3_

Figure S5 ^13^C NMR of dimer 3c in CDCl_3_

Figure S6 IR spectra of 3c

Figure S7 ^1^H NMR of dimer 3d in CDCl_3_

Figure S8 ^13^C NMR of dimer 3d in CDCl_3_

Figure S9 IR spectra of 3d

Figure S10 ^1^H NMR of dimer 3e in DMSO-*d*_6_

Figure S11 ^13^C NMR of dimer 3e in DMSO-*d*_6_

Figure S12 IR spectra of 3e

Figure S13 ^1^H NMR of dimer 9b in CDCl_3_

Figure S14 ^13^C NMR of dimer 9b in CDCl_3_

Figure S15 ^1^H NMR of dimer 9c in CDCl_3_

Figure S16 ^1^H NMR of polymer P1-3a in DMSO-*d*_6_

Figure S17 IR spectra of polymer P1-3a

Figure S18 ^1^H NMR of polymer P2-3b in DMSO-*d*_6_

Figure S19 IR spectra of polymer P2-3b

Figure S20 ^1^H NMR of polymer P3-3c in DMSO-*d*_6_

Figure S21 IR spectra of polymer P3-3c

Figure S22 ^1^H NMR of polymer P4-3d in DMSO-*d*_6_

Figure S23 IR spectra of polymer P4-3d

Figure S24 ^1^H NMR of polymer P5-3e in DMSO-*d*_6_

Figure S25 IR spectra of polymer P5-3e

Figure S26 ^1^H NMR of polymer P6-3b in DMSO-*d*_6_

Figure S27 IR spectra of polymer P6-3b

Figure S28 ^1^H NMR of polymer P7-3b in DMSO-*d*_6_

Figure S29 IR spectra of polymer P7-3b

Figure S30 ^1^H NMR of polymer P8-6a in DMSO-*d*_6_

Figure S31 IR spectra of polymer P8-6a

Figure S32 ^1^H NMR of polymer P9-6b in DMSO-*d*_6_

Figure S33 IR spectra of polymer P9-6b

Figure S34 HPLC chromatogram of asymmetric compound, 13
Table 2, entry 1
99% *ee*

Figure S35 HPLC chromatogram of asymmetric compound, 13
Table 2, entry 2
99% *ee*

Figure S36 HPLC chromatogram of asymmetric compound, 13
Table 2, entry 3
98% *ee*

Figure S37 HPLC chromatogram of asymmetric compound, 13
Table 2, entry 4
99% *ee*

Figure S38 HPLC chromatogram of asymmetric compound, 13
Table 2, entry 5
97% *ee*

Figure S39 HPLC chromatogram of asymmetric compound, 13
Table 2, entry 6
44% *ee*

HPLC data of the products obtained from Enantioselective Michael Addition of Methyl 2-oxocyclopentanecarboxylate, 11 to *trans-*β-Nitrostyrene, 12

Figure S40 HPLC chromatogram of asymmetric compound, 13
Table 2, entry 7
99% *ee*

Figure S41 HPLC chromatogram of asymmetric compound, 13
Table 3, entry 1
99% *ee*

Figure S42 HPLC chromatogram of asymmetric compound, 13
Table 3, entry 2
>99% *ee*

Figure S43 HPLC chromatogram of asymmetric compound, 13
Table 3, entry 3
98% *ee*

Figure S44 HPLC chromatogram of asymmetric compound, 13
Table 3, entry 4
98% *ee*

Figure S45 HPLC chromatogram of asymmetric compound, 13
Table 3, entry 5
99% *ee*

Figure S46 HPLC chromatogram of asymmetric compound, 13
Table 3, entry 6
94% *ee*

Figure S47 HPLC chromatogram of asymmetric compound, 13
Table 3, entry 7
96% *ee*

Figure S48 HPLC chromatogram of asymmetric compound, 13
Table 3, entry 8
64% *ee*

Figure S49 HPLC chromatogram of asymmetric compound, 13
Table 3, entry 9
>99% *ee*

Figure S50 HPLC chromatogram of asymmetric compound, 13
Table 4, entry 1
>99% *ee*

Figure S51 HPLC chromatogram of asymmetric compound, 13
Table 4, entry 2
98% *ee*

Figure S52 HPLC chromatogram of asymmetric compound, 13
Table 4, entry 3
95% *ee*

Figure S53 HPLC chromatogram of asymmetric compound, 13
Table 4, entry 4
97% *ee*

Figure S54 HPLC chromatogram of asymmetric compound, 13
Table 4, entry 5
97% *ee*

Figure S55 HPLC chromatogram of asymmetric compound, 13
Table 4, entry 6
96% *ee*

Figure S56 HPLC chromatogram of asymmetric compound, 13
Table 4, entry 7
98% *ee*

Figure S57 HPLC chromatogram of asymmetric compound, 18
Table 5, entry 1
92% *ee*

Figure S58 HPLC chromatogram of asymmetric compound, 19
Table 5, entry 2
73% *ee*

Figure S59 HPLC chromatogram of asymmetric compound, 20
Table 5, entry 3
>99% *ee*

Figure S60 HPLC chromatogram of asymmetric compound, 21
Table 5, entry 4
99% *ee*

Figure S61 HPLC chromatogram of asymmetric compound, 13
Table 6, entry 1, fresh
>99% *ee*

Figure S62 HPLC chromatogram of asymmetric compound, 13
Table 6, entry 2, cycle 1
97% *ee*

Figure S63 HPLC chromatogram of asymmetric compound, 13
Table 6, entry 3, cycle 2
99% *ee*

Figure S64 HPLC chromatogram of asymmetric compound, 13
Table 6, entry 4, cycle 3
98% *ee*

Figure S65 HPLC chromatogram of asymmetric compound, 13
Table 6, entry 5, cycle 4
99% *ee*

## Figures and Tables

**Table 1:** Synthesis of chiral hyperbranched polymers of different cinchona dimers and trifunctional aromatic iodides by applying Mizoroki–Heck polymerization.

$$\text{Dimer}+\text{Triiodide}\overset{\text{Pd}{\left(\text{OAc}\right)}_{2},10\;\text{mol}\text{.}\;\%}{\underset{\text{DMF},\;100\;{}^{\circ}\text{C},48\;\text{h}}{⟶}}\text{Hyperbranched}\;\text{polymer}$$							
Entry	Dimer	Iodides	Chiral HBP	Yield [%]	*M*_n_ a	*M*_w_ a	*M*_w_* /M*_n_ a
1	**3a**	**9a**	**P1-3a**	79	8000	13,000	1.65
2	**3b**	**9a**	**P2-3b**	81	10,000	19,000	1.97
3	**3c**	**9a**	**P3-3c**	70	24,000	63,000	2.63
4	**3d**	**9a**	**P4-3d**	86	23,000	61,000	2.72
5	**3e**	**9a**	**P5-3e**	55	16,000	23,000	1.43
6^b^	**3b**	**9b**	**P6-3b**	93	-	-	-
7^b^	**3b**	**9c**	**P7-3b**	88	-	-	-
8	**6a**	**9a**	**P8-6a**	73	15,000	25,000	1.67
9	**6b**	**9a**	**P9-6b**	77	18,000	27,000	1.52

a Determined by GPC with a flow rate of 1.0 mL per minute at 40 °C and DMF as the solvent (polystyrene standard).

b Not soluble in DMF.

**Table 2:** Asymmetric Michael addition^a^ of β-ketoesters (**11)** with *trans*-β-nitrostyrene (**12)** using various dimers.

Entry	Catalysts	Reaction time [h]	Yield^b^ [%]	dr^c^ [%]	ee^c^ [%]
1	**3a**	24	93	7.6:1	99
2	**3b**	24	96	15:1	99
3	**3c**	28	79	7.9:1	98
4	**3d**	42	62	4.7:1	99
5	**3e**	3	92	10:1	97
6	**6a**	32	72	0.6:1	44
7	**6b**	20	94	5.3:1	99

a At room temperature, asymmetric reactions involving **11** (0.50 mmol), **12** (0.55 mmol), and the dimeric catalyst (5 mol.%) were conducted in 2.5 mL of CH_2_Cl_2_.

b Isolated yield after purification by column chromatography.

c Enantioselectivity (ee) as assessed by HPLC (flow rate: 1.0 mL/min on chiral Cel OD-H).

**Table 3:** Asymmetric Michael addition^a^ of β-ketoesters (**11**) with *trans*-β-nitrostyrene (**12**) using different HBPs.

Entry	Catalysts	Reaction time [h]	Yield^b^ [%]	dr^c^ [%]	ee^c^ [%]
1	**P1-3a**	36	96	4.5:1	99
2	**P2-3b**	24	84	8:1	>99
3	**P3-3c**	30	86	6.4:1	98
4	**P4-3d**	36	81	6.4:1	98
5	**P5-3e**	24	75	5.5:1	99
6	**P6-3b**	36	63	10.5:1	94
7	**P7-3b**	36	67	11.3:1	96
8	**P8-6a**	24	59	1.1:1	64
9	**P9-6b**	24	73	5.9:1	>99

a At room temperature, asymmetric reactions involving **11** (0.50 mmol), **12** (0.55 mmol), and the polymeric catalyst (5 mol.%) were conducted in 2.5 mL of CH_2_Cl_2_.

b Isolated yield after purification by column chromatography.

c Enantioselectivity (ee) as assessed by HPLC (flow rate: 1.0 mL/min on chiral Cel OD-H).

**Table 4:** Asymmetric Michael addition^a^ of β-ketoesters **11** to *trans*-β-nitrostyrene **12** using hyperbranched polymeric catalyst **P2-3b** in different solvents.

Entry	Solvent	Yield^b^ [%]	dr^c^ [%]	ee^c^ [%]
1	CH_2_Cl_2_	84	8:1	>99
2	Acetone	60	6:1	98
3	MeOH	70	3.7:1	95
4	EtOAc	27	3.4:1	97
5	Hexene	81	7.9:1	97
6	THF	52	6.6:1	96
7	CH_3_CN	55	4.9:1	98

a At room temperature, asymmetric reactions involving **11** (0.50 mmol), **12** (0.55 mmol), and the polymeric catalyst (5 mol.%) were conducted in 2.5 mL of CH_2_Cl_2_.

b Isolated yield after purification by column chromatography.

c Enantioselectivity (ee) as assessed by HPLC (flow rate: 1.0 mL/min on chiral Cel OD-H).

**Table 5:** Enantioselective Michael addition^a^ reaction resulting from combinations of different donors and acceptors using polymeric catalyst **P2-3b**.

Entry	Michael donor	Michael acceptor	Product	Reaction time [h]	Yield^b^ [%]	dr^c^ [%]	ee^c^ [%]
1	**14**	**12**	**18**	42	77	14.4:1	92
2	**11**	**15**	**19**	48	87	9.3:1	73
3	**11**	**16**	**20**	46	82	1.7:1	>99
4	**11**	**17**	**21**	38	89	13:1	99

a At room temperature, asymmetric reactions involving **11** (0.50 mmol), **12** (0.55 mmol), and the polymeric catalyst (5 mol.%) were conducted in 2.5 mL of CH_2_Cl_2_.

b Isolated yield after purification by column chromatography.

c Enantioselectivity (ee) as assessed by HPLC (flow rate: 1.0 mL/min on chiral Cel OD-H).

**Table 6:** Enantioselective Michael addition^a^ of β-ketoesters **11** with *trans*-β-nitrostyrene **12** using HBP **P2-3a** to examine recyclability performance.

Entry	Cycle	Reaction time [h]	Yield^b^ [%]	dr^c^ [%]	ee^c^ [%]
1	Fresh	24	84	8:1	>99
2	1	24	77	9.8:1	97
3	2	30	85	9.4:1	99
4	3	30	81	7.8:1	98
5	4	36	67	8.6:1	99

a At room temperature, asymmetric reactions involving **11** (0.50 mmol), **12** (0.55 mmol), and the polymeric catalyst (5 mol.%) were conducted in 2.5 mL of CH_2_Cl_2_.

b Isolated yield after purification by column chromatography.

c Enantioselectivity (ee) as assessed by HPLC (flow rate: 1.0 mL/min on chiral Cel OD-H).
