# Supplementary figures and images for: Preparation of a chiral hyperbranched polymer based on cinchona alkaloids and investigation of its catalytic activity in asymmetric reactions
Source: Turk J Chem. 2024 Jan 2;48(4):512–23. doi: 10.55730/1300-0527.3677 (PMC11407360; doi:10.55730/1300-0527.3677)

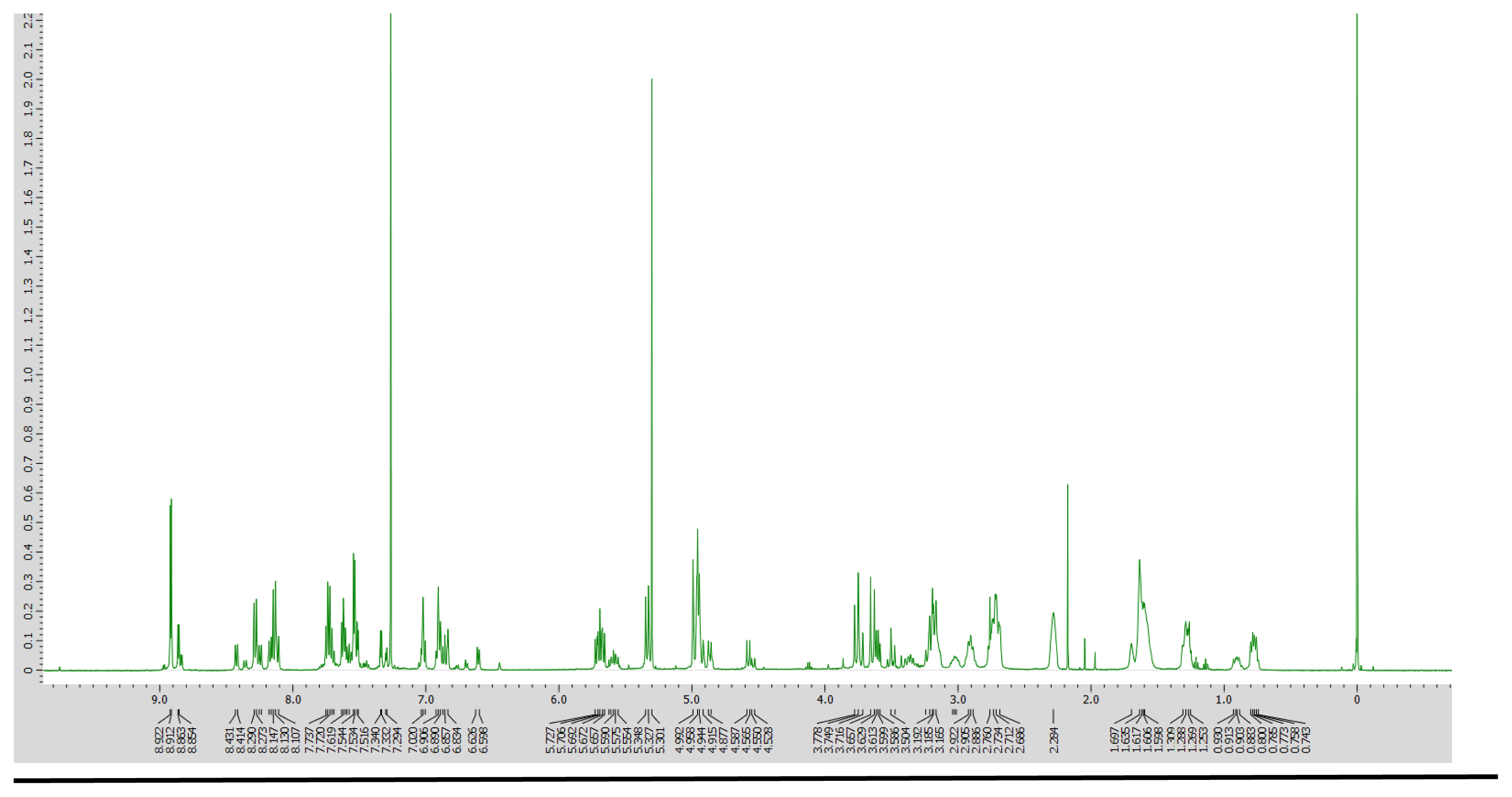

Supplement: Figure S1 — 1H NMR of dimer 3b in CDCl3 [file tjc-48-04-512s1.tif]

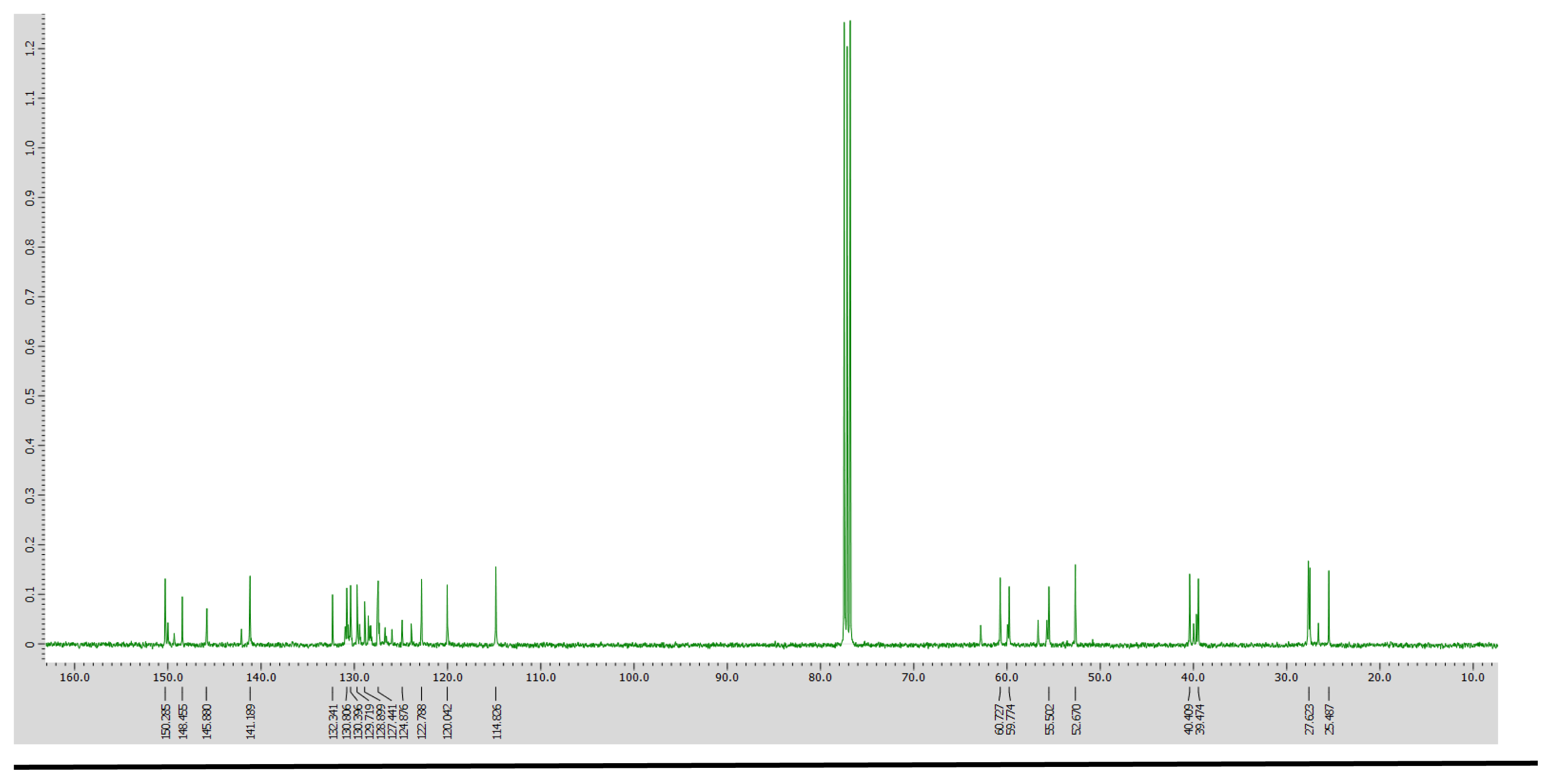

Supplement: Figure S2 — 13C NMR of dimer 3b in CDCl3 [file tjc-48-04-512s2.tif]

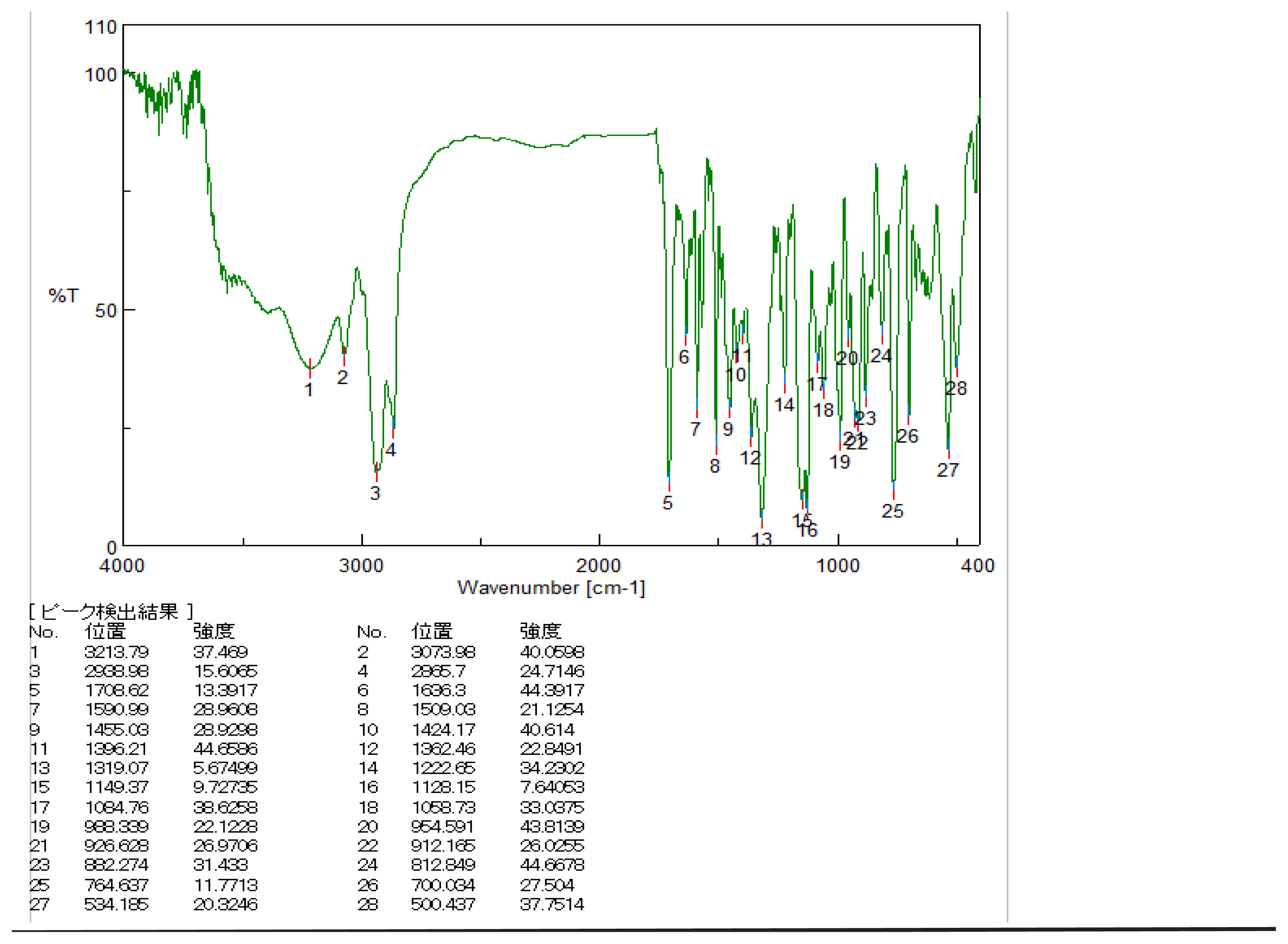

Supplement: Figure S3 — IR spectra of 3b [file tjc-48-04-512s3.tif]

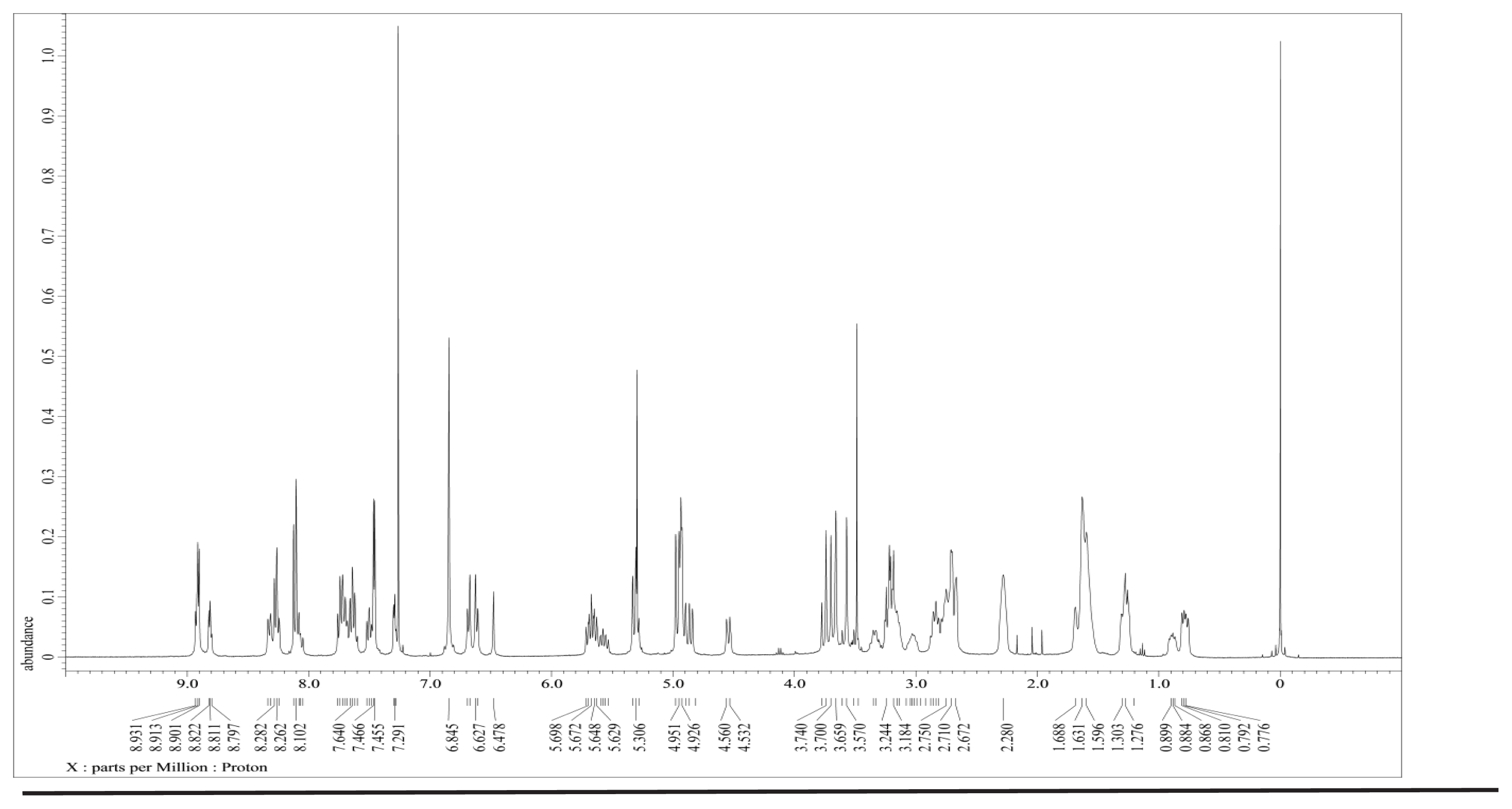

Supplement: Figure S4 — 1H NMR of dimer 3c in CDCl3 [file tjc-48-04-512s4.tif]

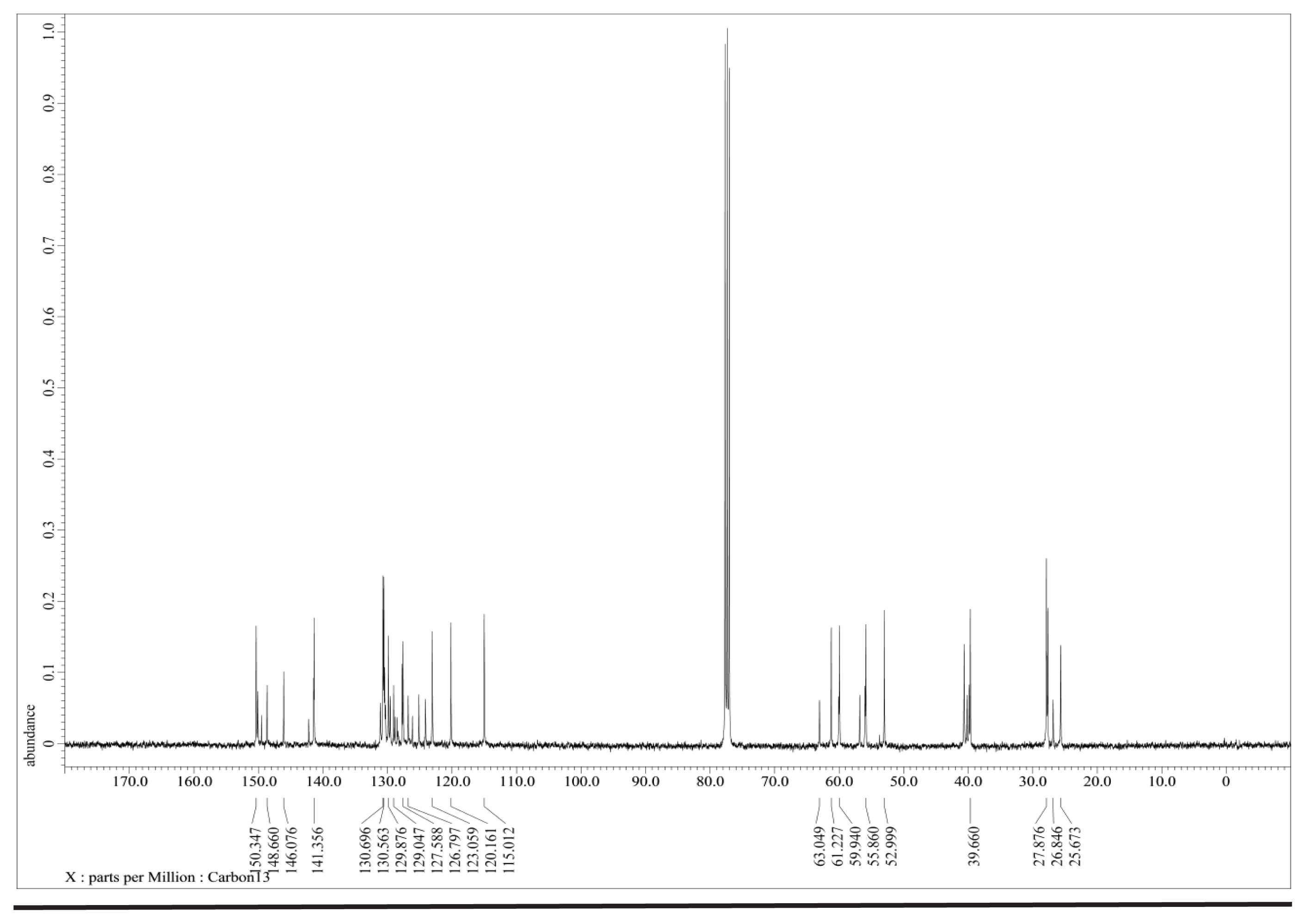

Supplement: Figure S5 — 13C NMR of dimer 3c in CDCl3 [file tjc-48-04-512s5.tif]

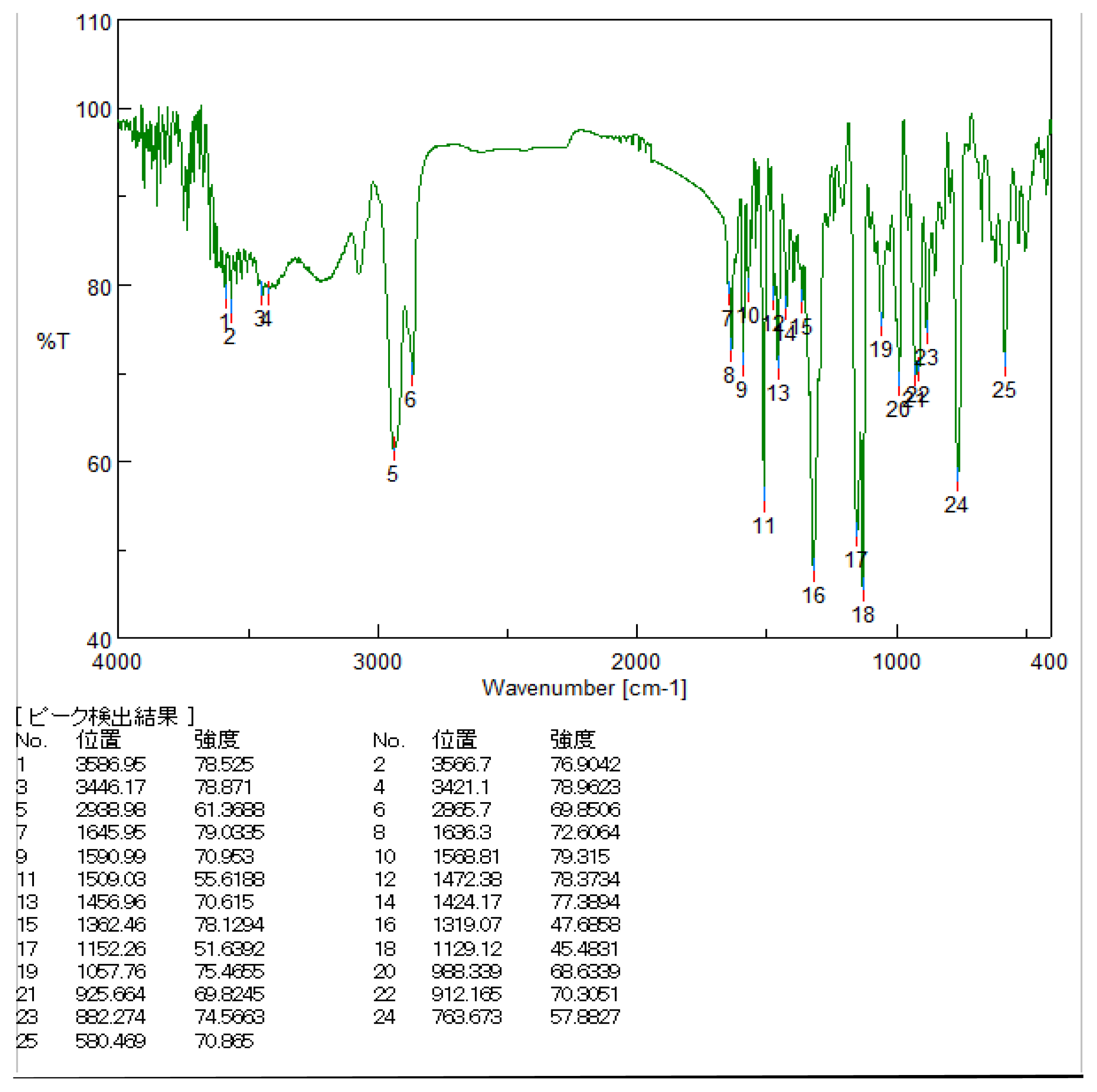

Supplement: Figure S6 — IR spectra of 3c [file tjc-48-04-512s6.tif]

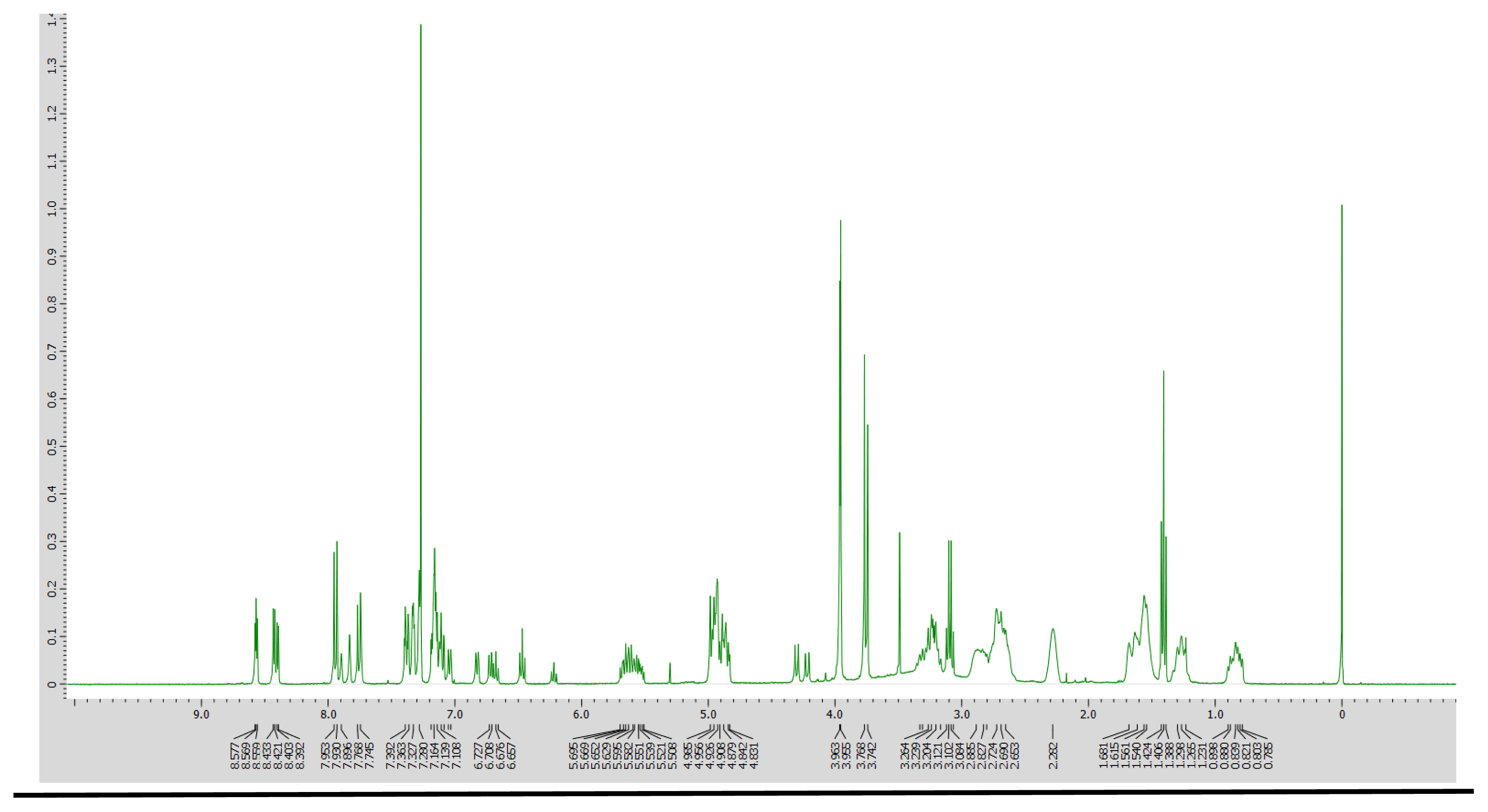

Supplement: Figure S7 — 1H NMR of dimer 3d in CDCl3 [file tjc-48-04-512s7.tif]

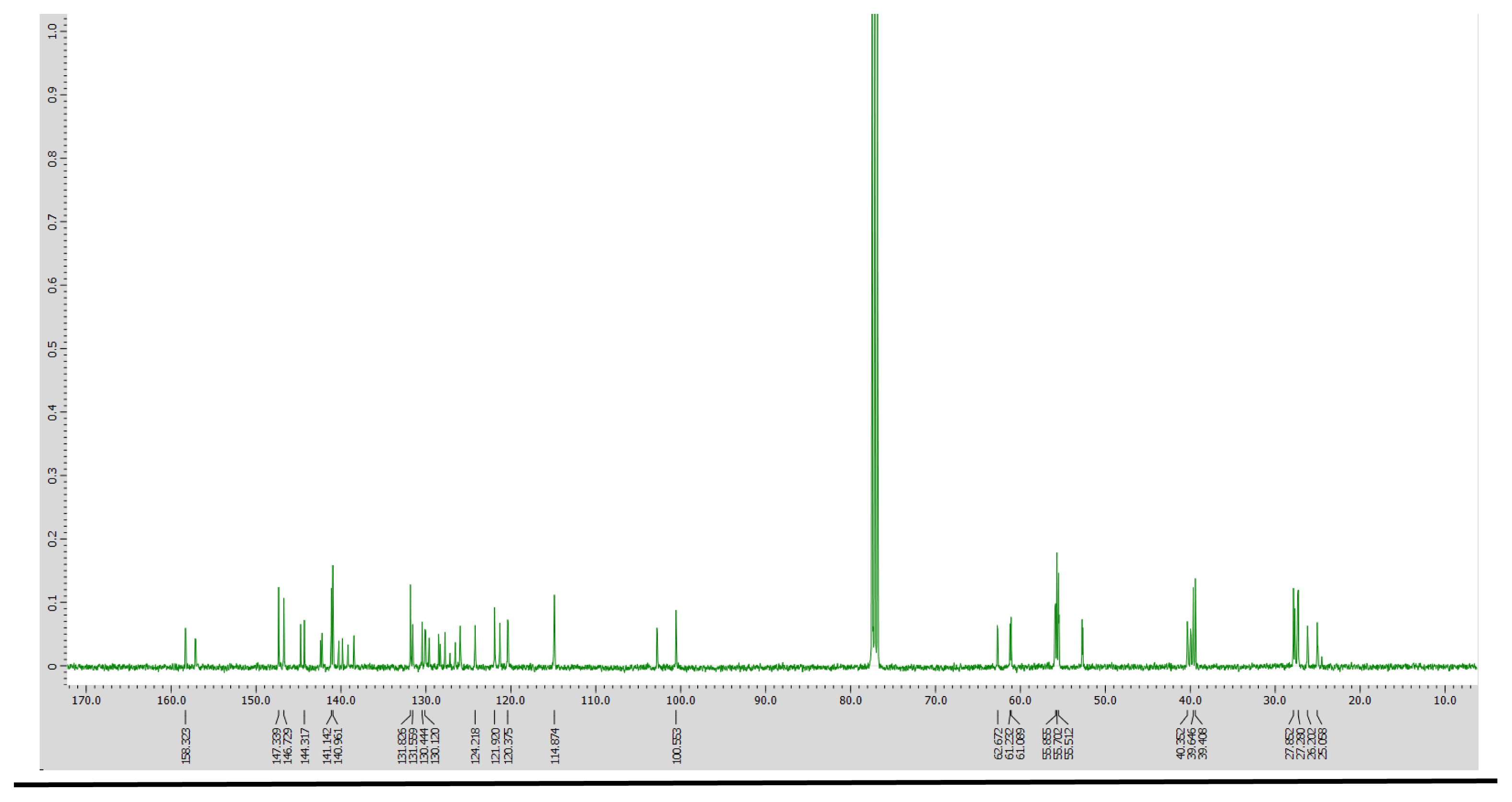

Supplement: Figure S8 — 13C NMR of dimer 3d in CDCl3 [file tjc-48-04-512s8.tif]

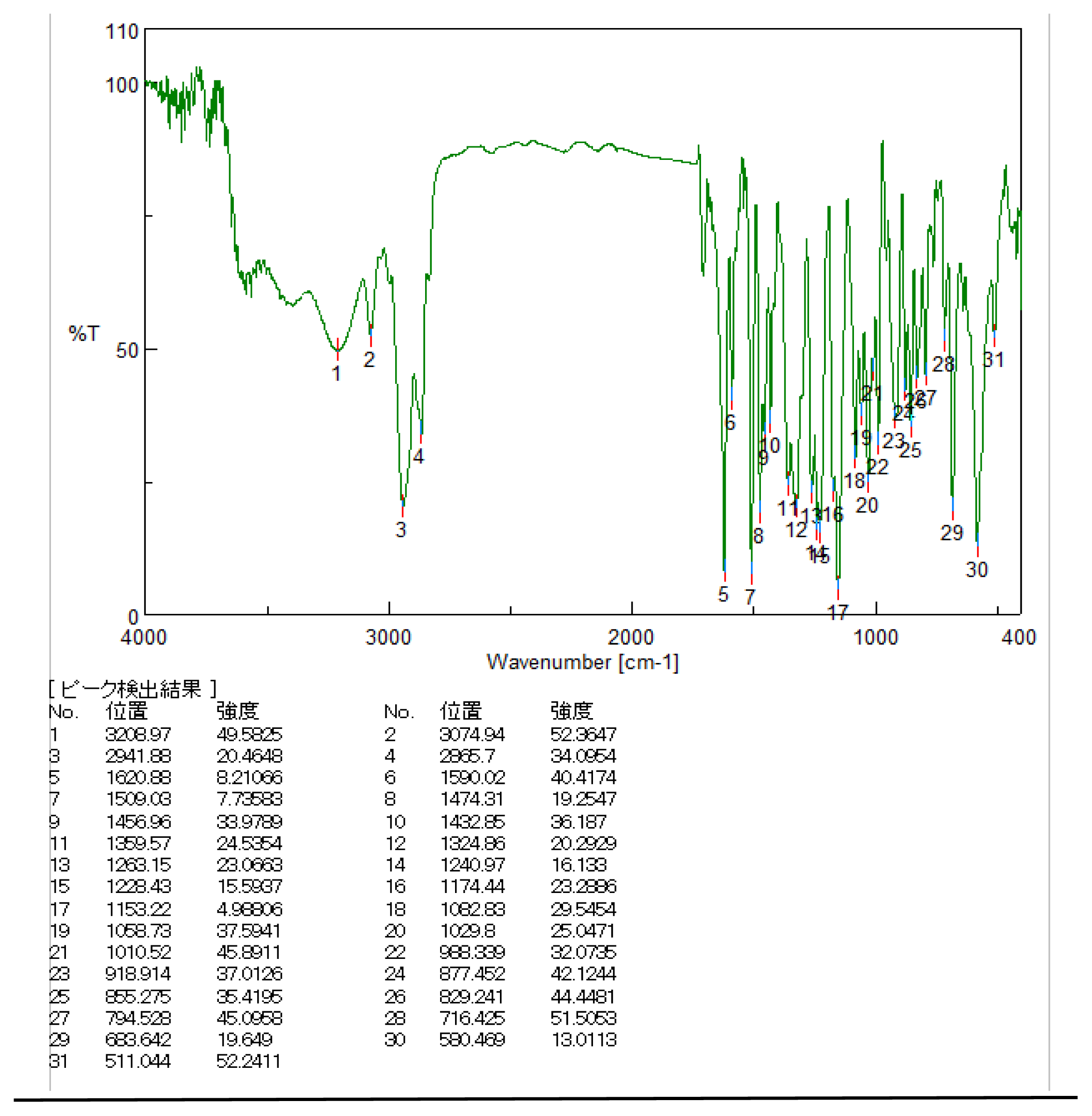

Supplement: Figure S9 — IR spectra of 3d [file tjc-48-04-512s9.tif]

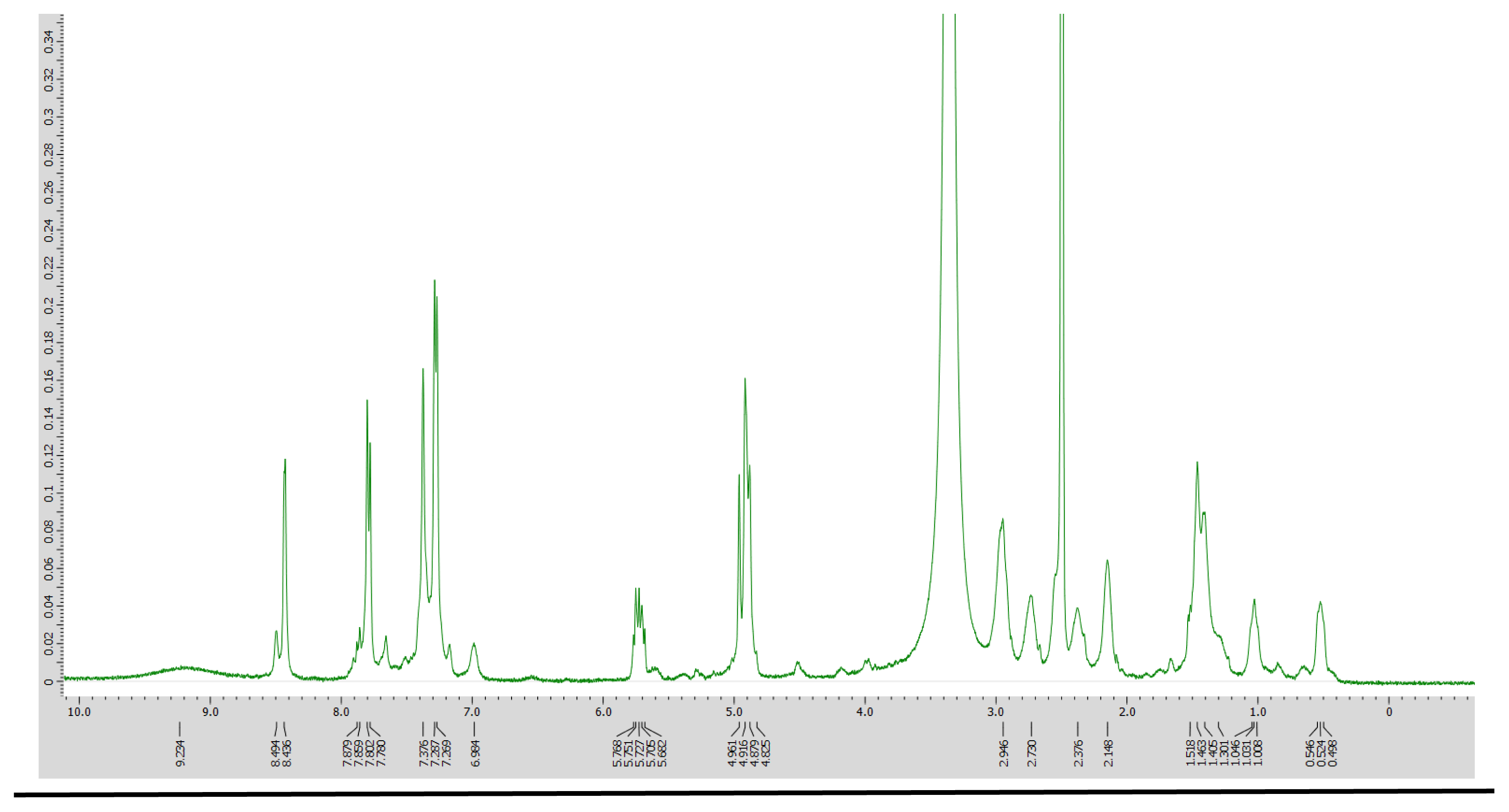

Supplement: Figure S10 — 1H NMR of dimer 3e in DMSO-d6 [file tjc-48-04-512s10.tif]

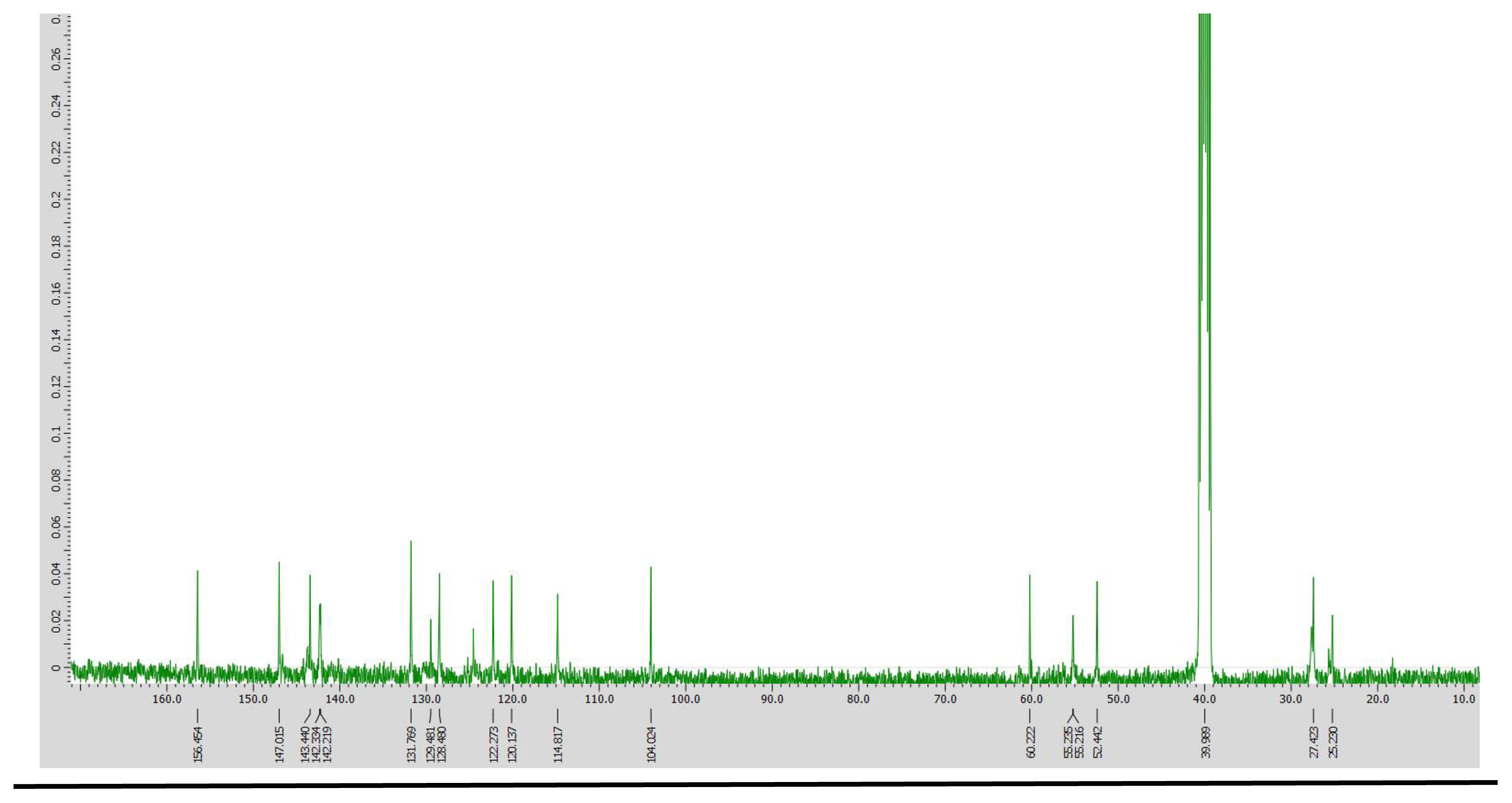

Supplement: Figure S11 — 13C NMR of dimer 3e in DMSO-d6 [file tjc-48-04-512s11.tif]

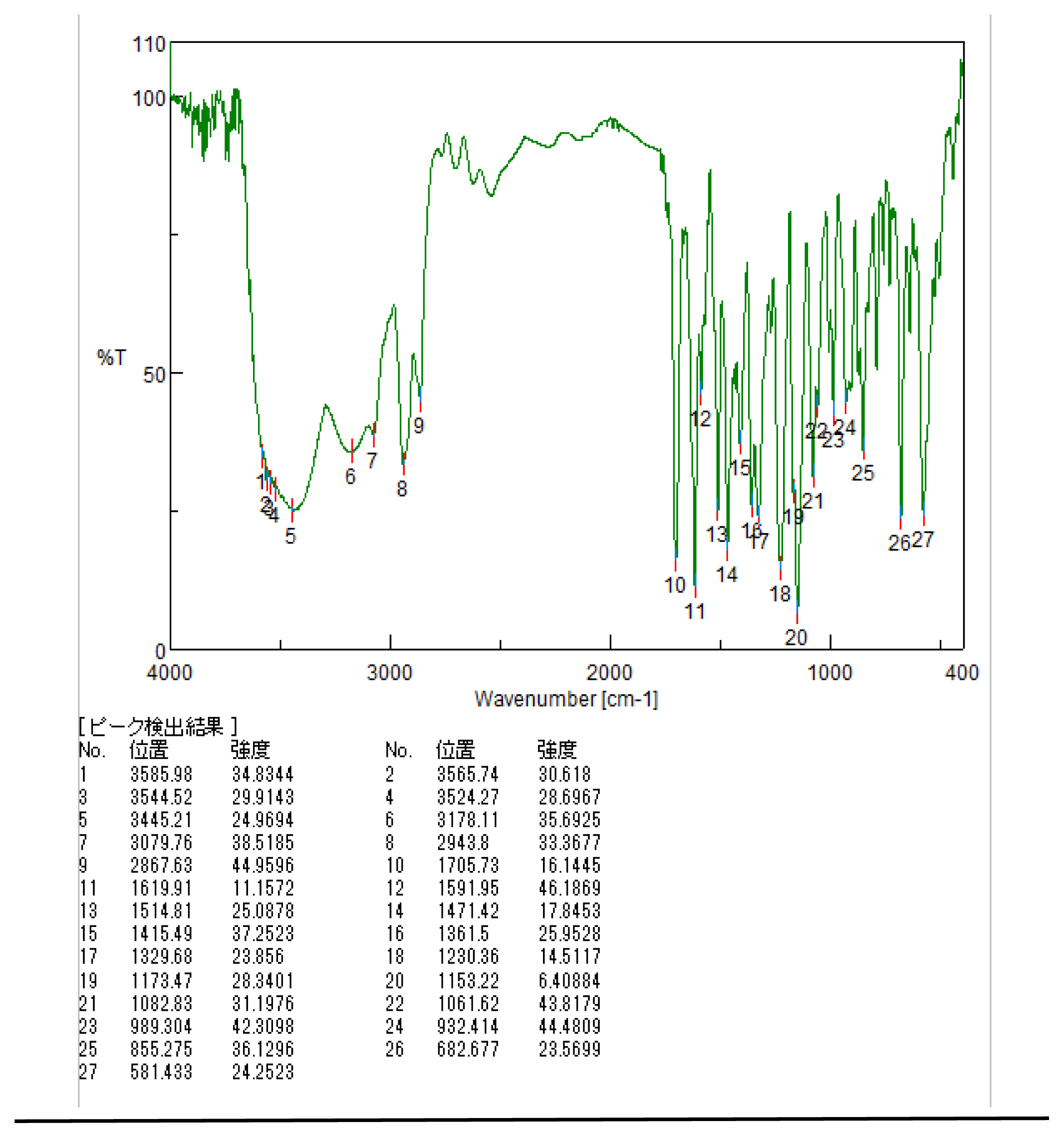

Supplement: Figure S12 — IR spectra of 3e [file tjc-48-04-512s12.tif]

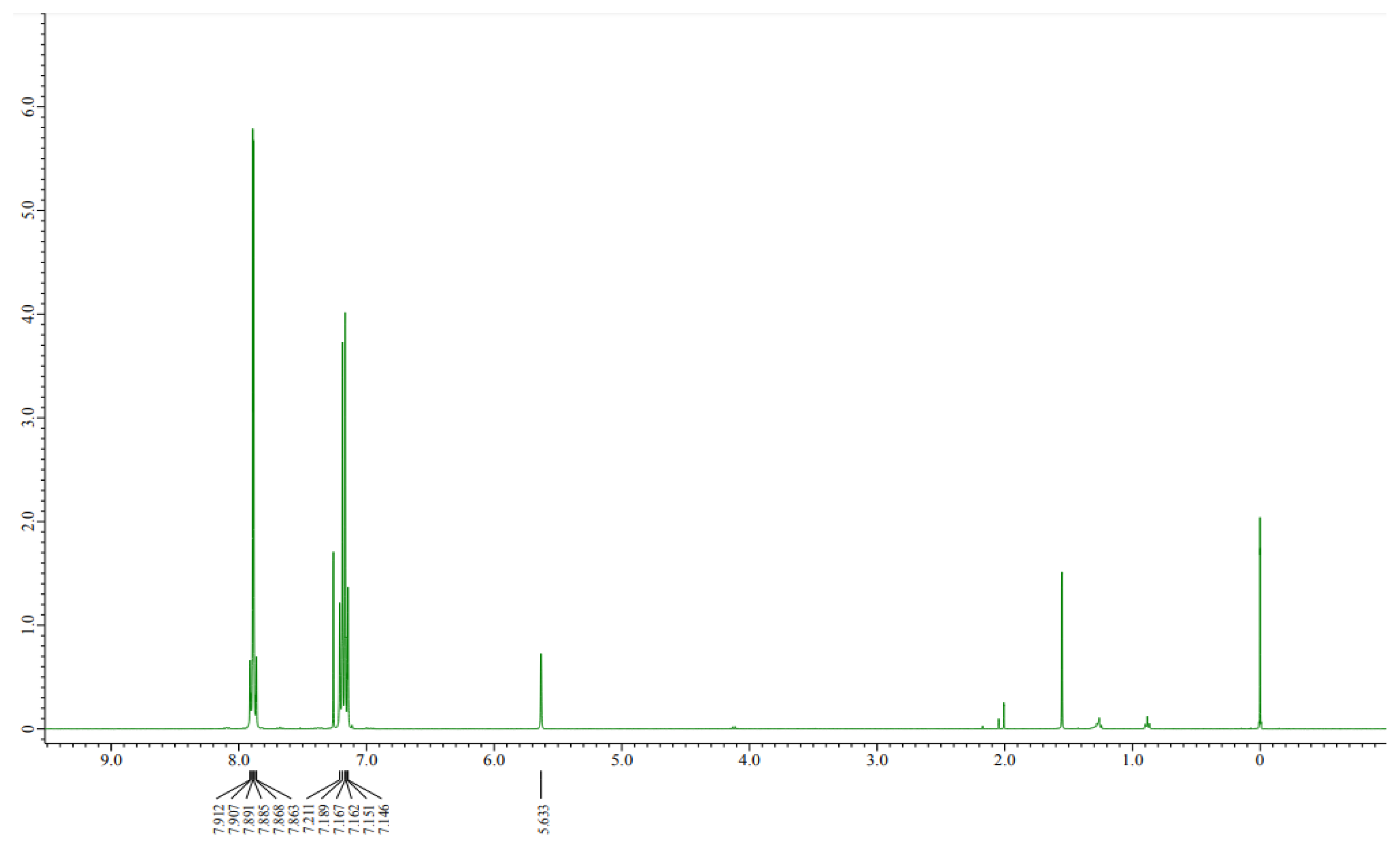

Supplement: Figure S13 — 1H NMR of dimer 9b in CDCl3 [file tjc-48-04-512s13.tif]

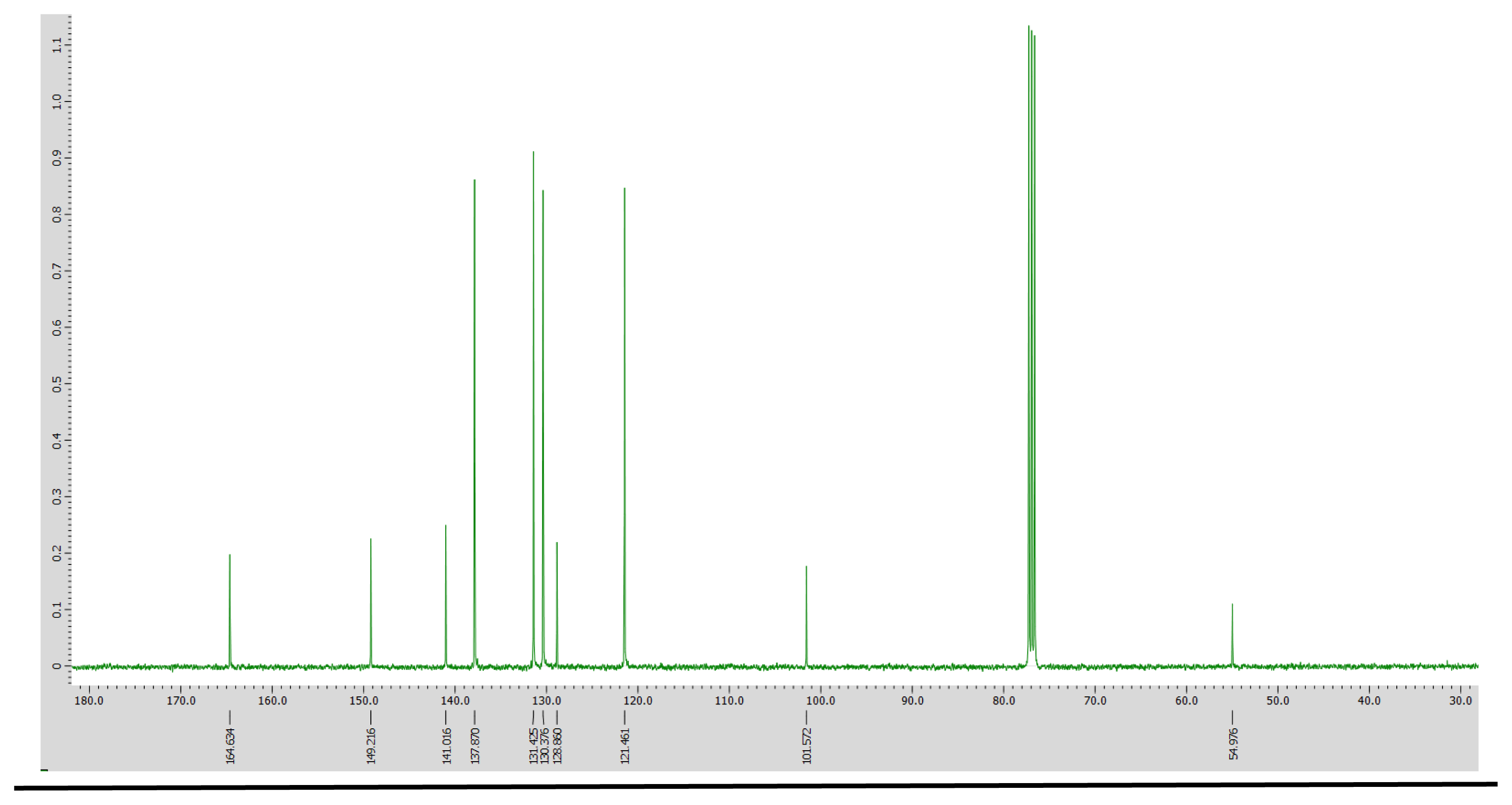

Supplement: Figure S14 — 13C NMR of dimer 9b in CDCl3 [file tjc-48-04-512s14.tif]

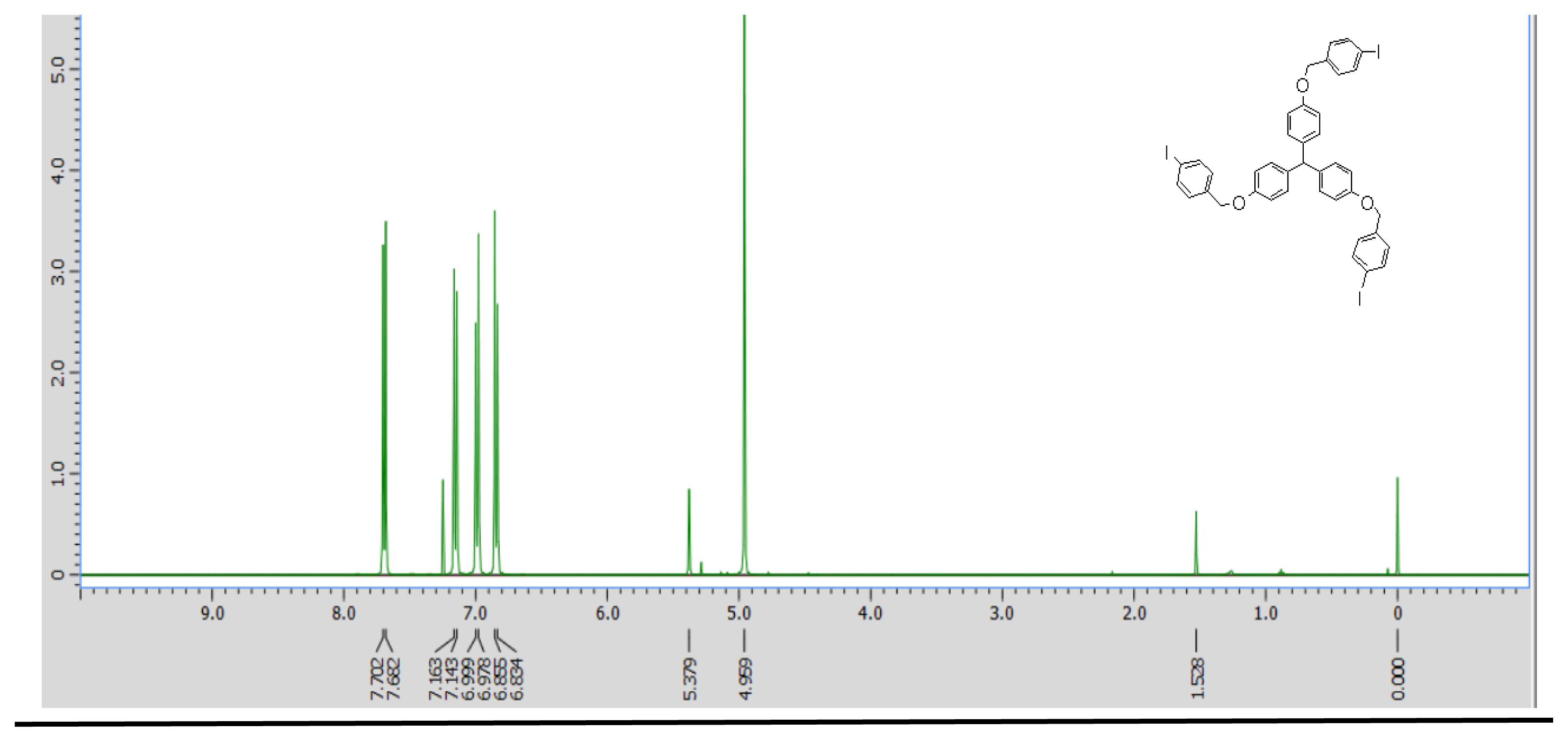

Supplement: Figure S15 — 1H NMR of dimer 9c in CDCl3 [file tjc-48-04-512s15.tif]

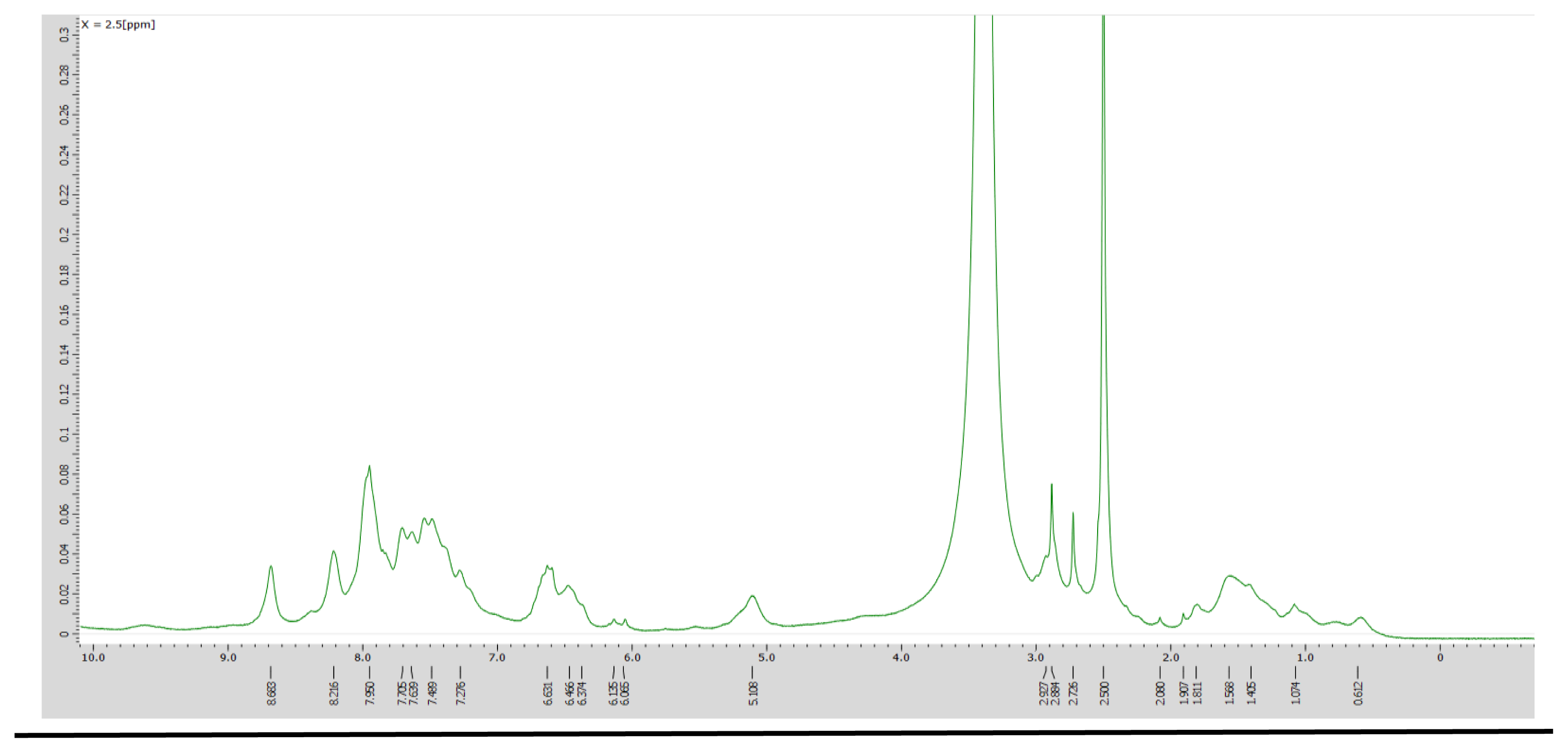

Supplement: Figure S16 — 1H NMR of polymer P1-3a in DMSO-d6 [file tjc-48-04-512s16.tif]

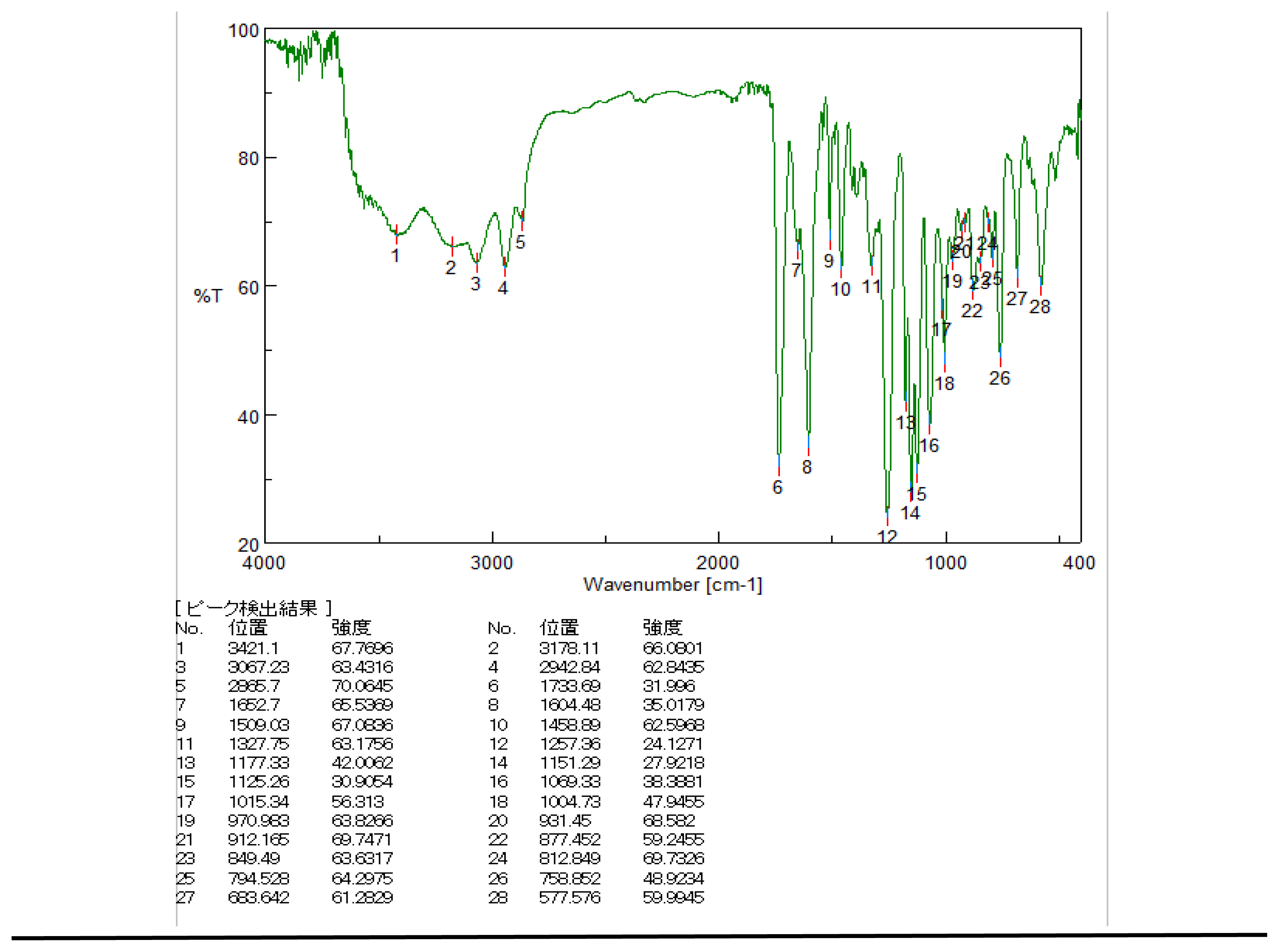

Supplement: Figure S17 — IR spectra of polymer P1-3a [file tjc-48-04-512s17.tif]

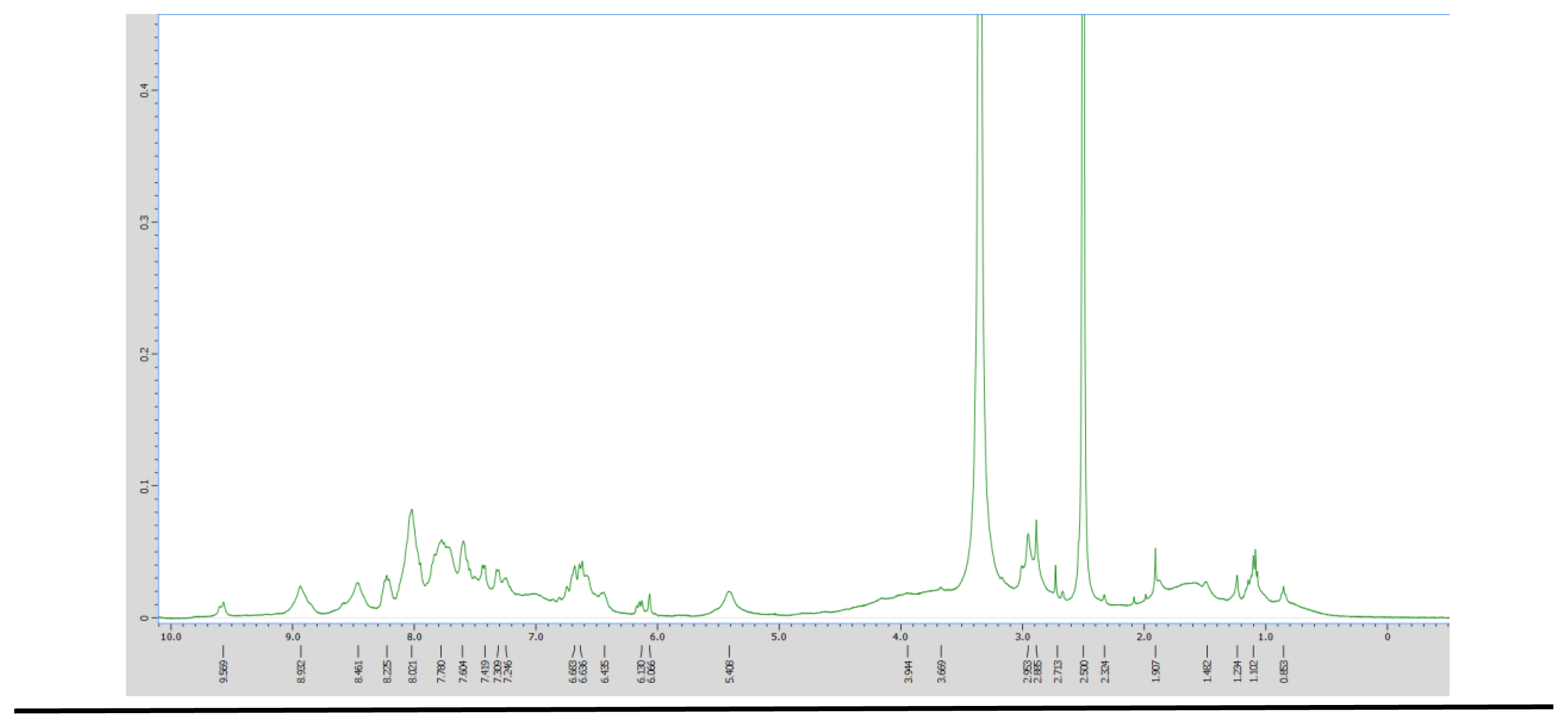

Supplement: Figure S18 — 1H NMR of polymer P2-3b in DMSO-d6 [file tjc-48-04-512s18.tif]

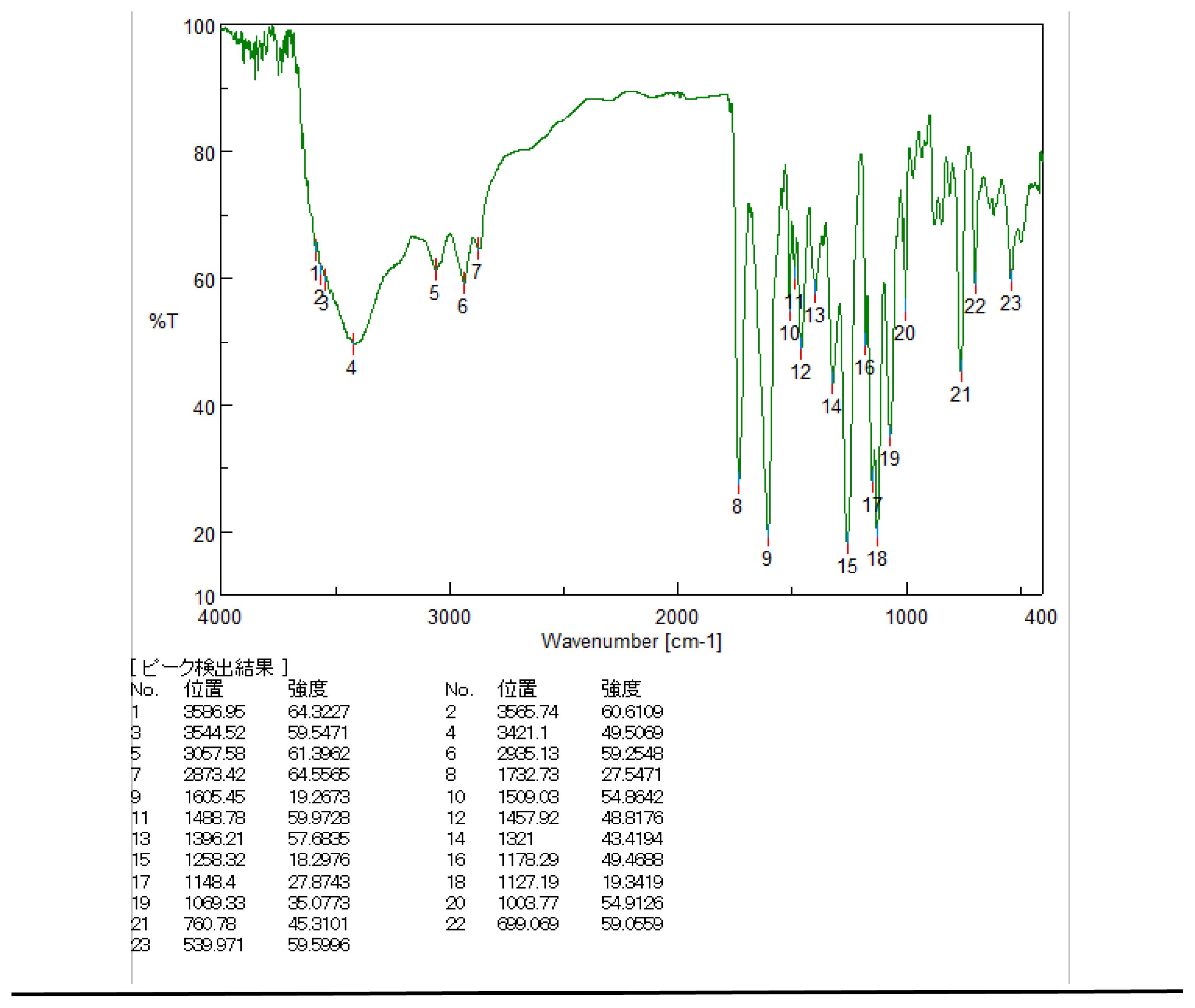

Supplement: Figure S19 — IR spectra of polymer P2-3b [file tjc-48-04-512s19.tif]

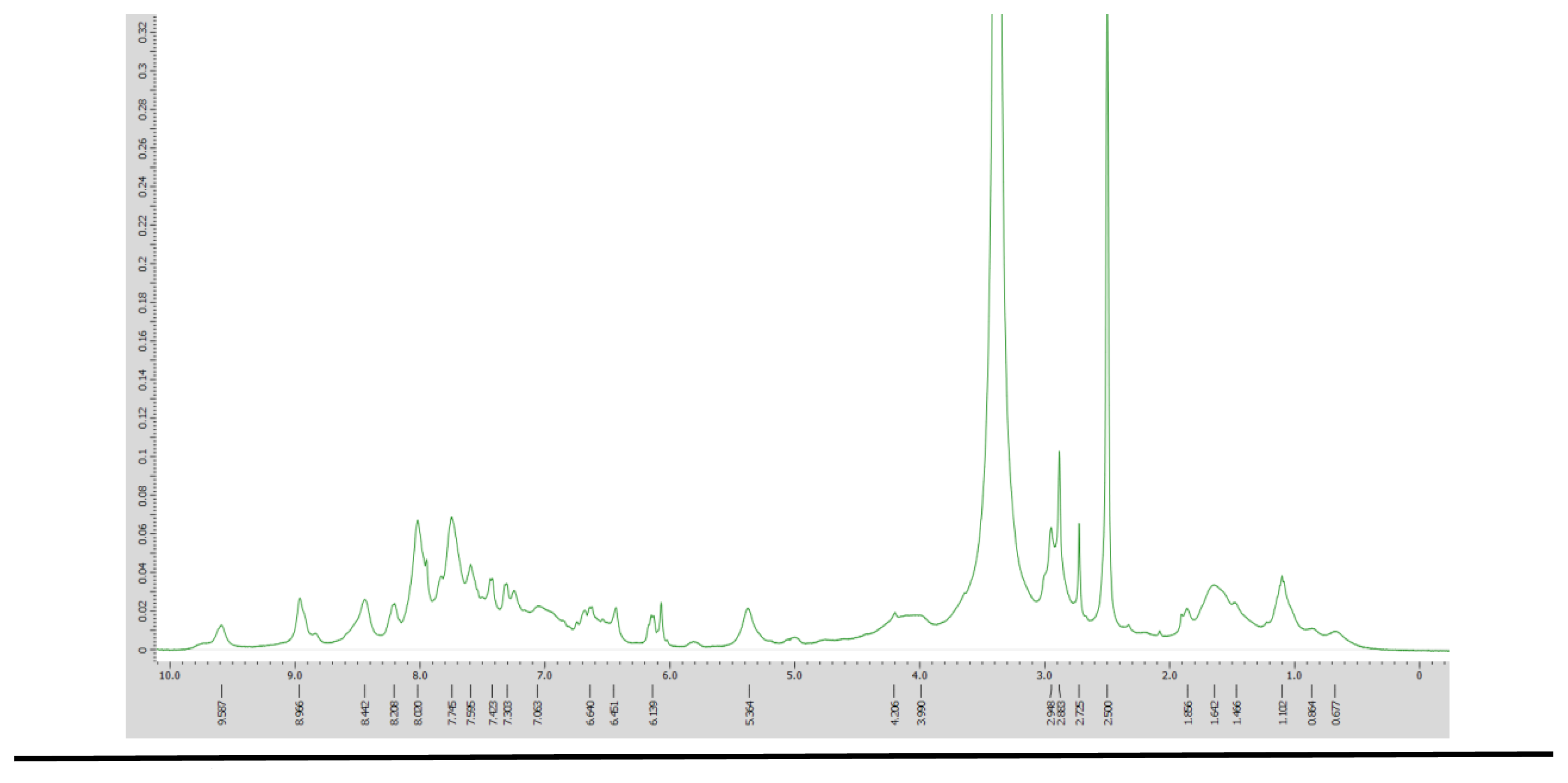

Supplement: Figure S20 — 1H NMR of polymer P3-3c in DMSO-d6 [file tjc-48-04-512s20.tif]

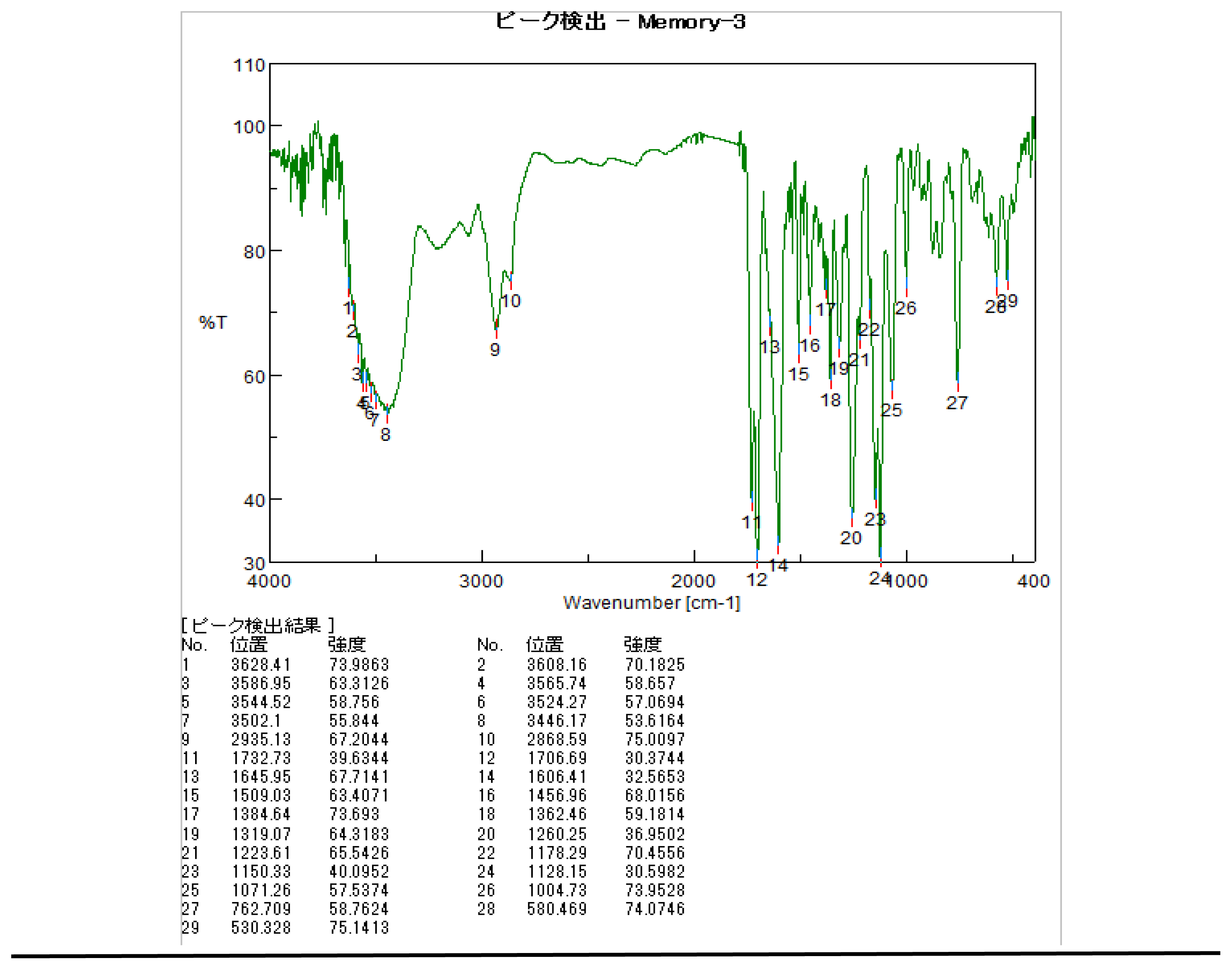

Supplement: Figure S21 — IR spectra of polymer P3-3c [file tjc-48-04-512s21.tif]

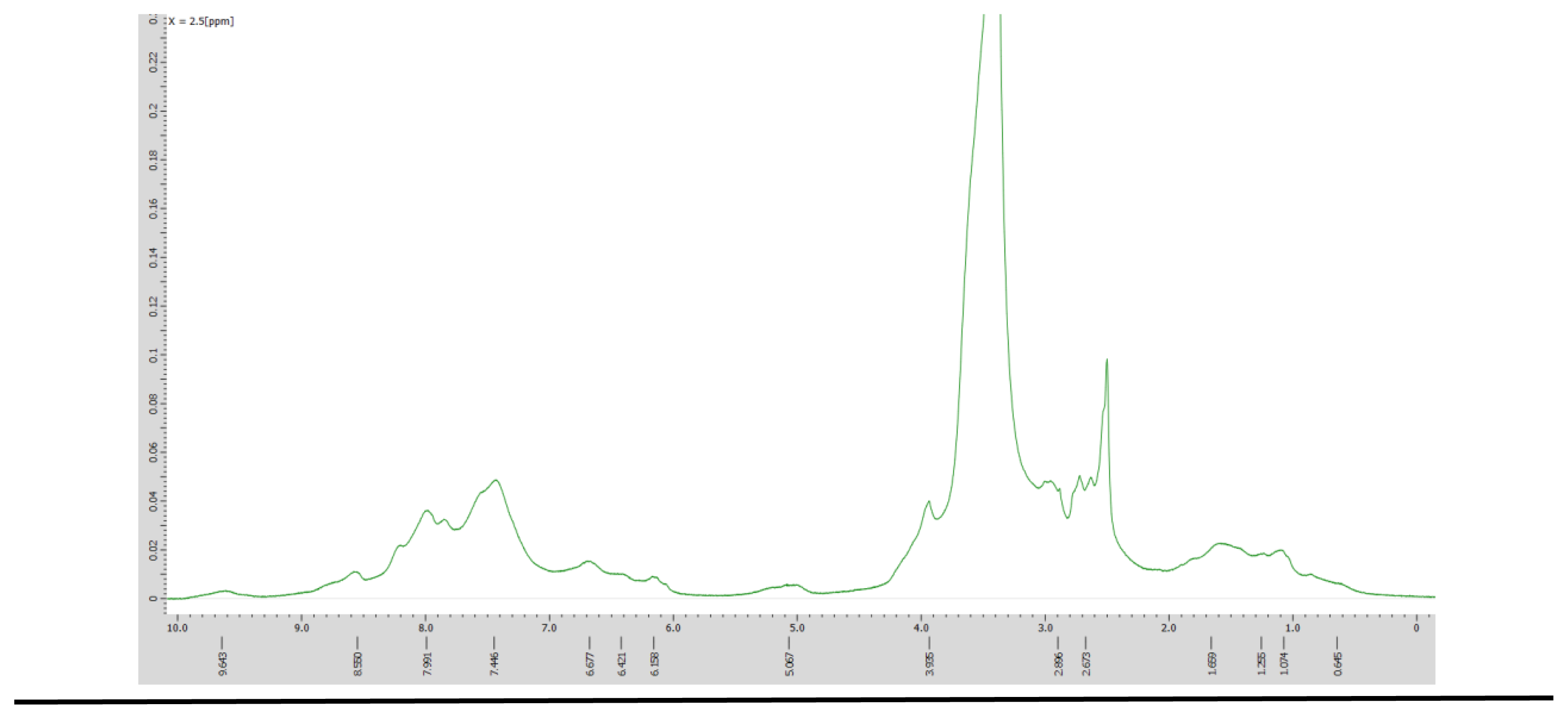

Supplement: Figure S22 — 1H NMR of polymer P4-3d in DMSO-d6 [file tjc-48-04-512s22.tif]

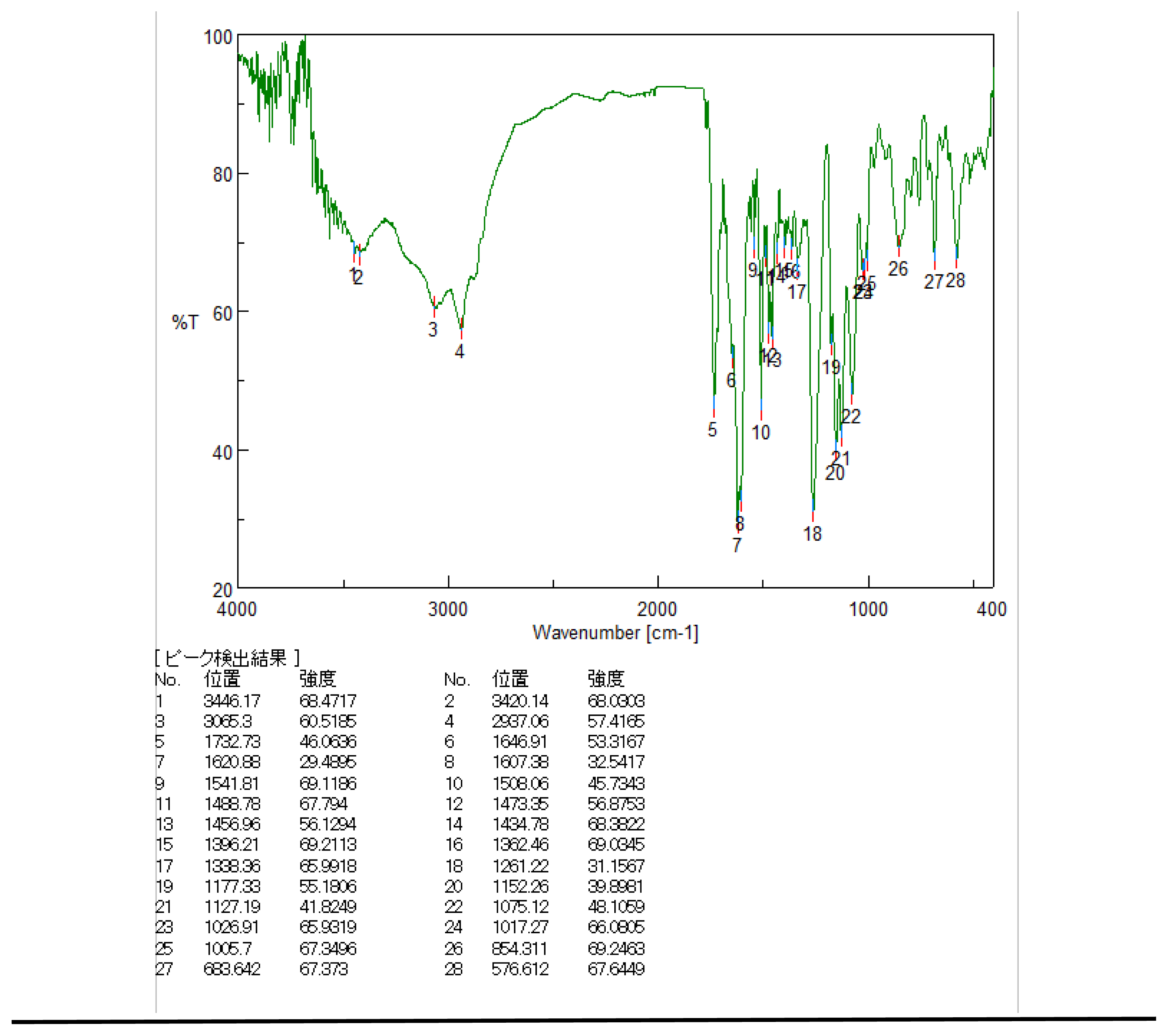

Supplement: Figure S23 — IR spectra of polymer P4-3d [file tjc-48-04-512s23.tif]

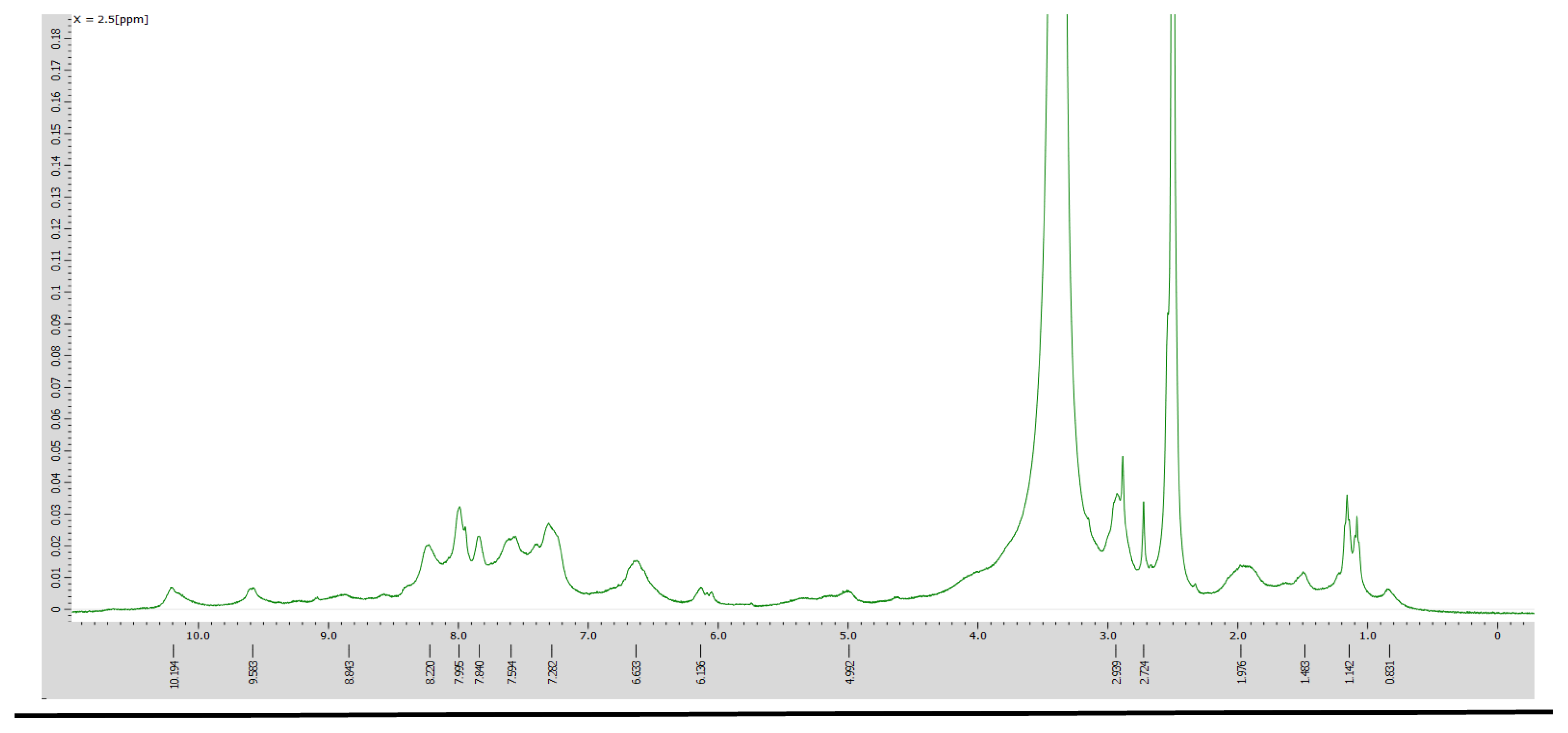

Supplement: Figure S24 — 1H NMR of polymer P5-3e in DMSO-d6 [file tjc-48-04-512s24.tif]

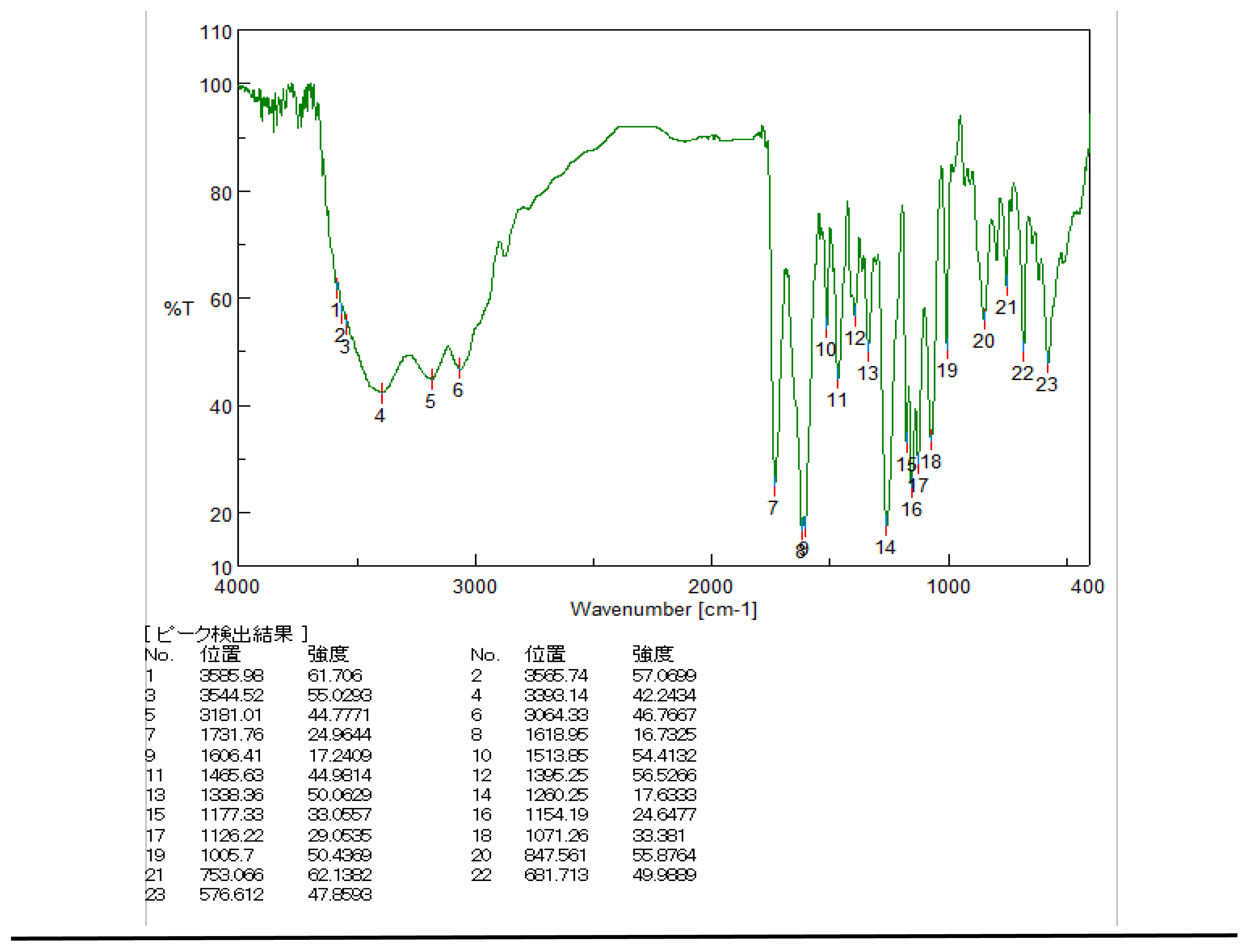

Supplement: Figure S25 — IR spectra of polymer P5-3e [file tjc-48-04-512s25.tif]

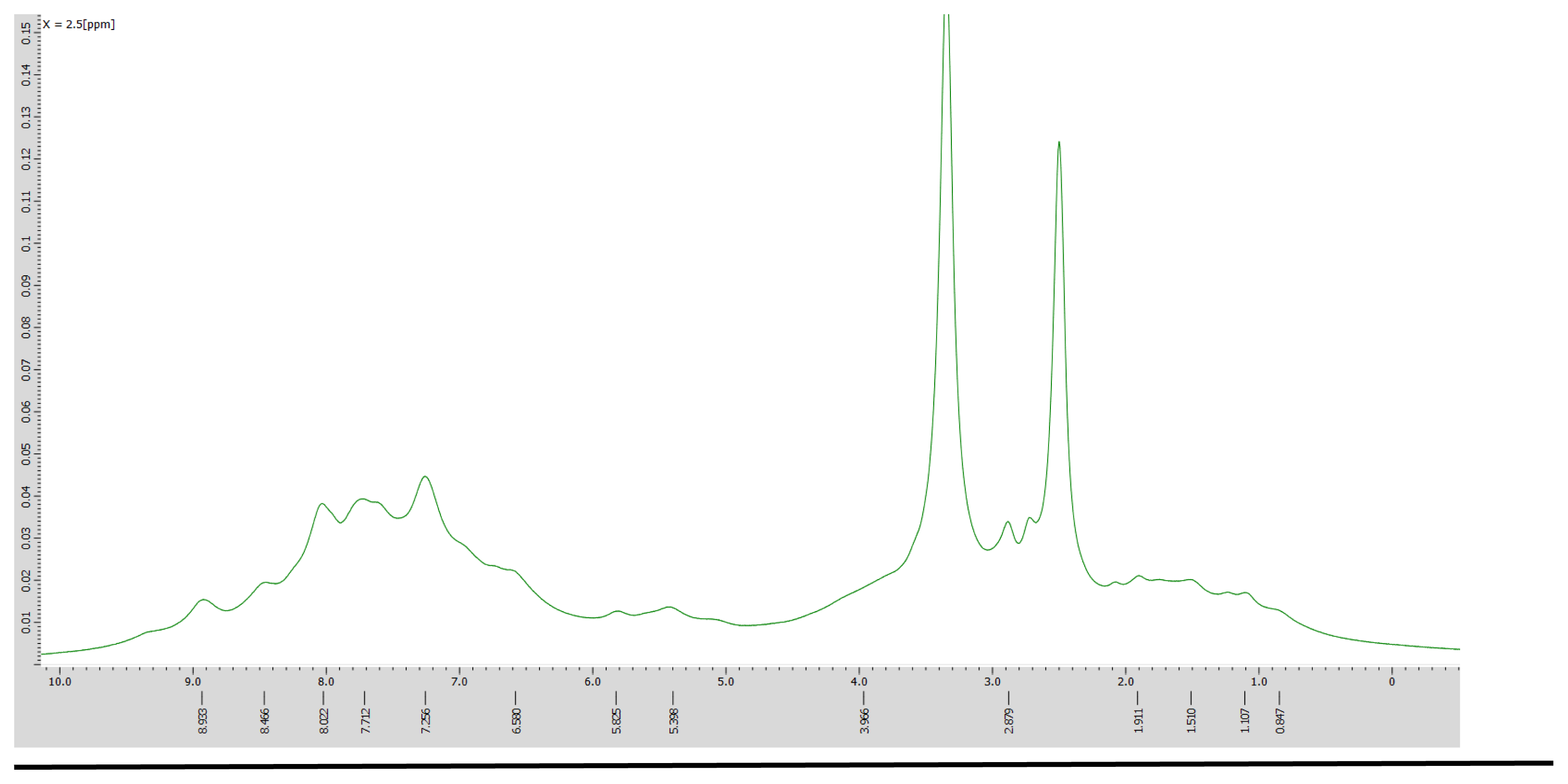

Supplement: Figure S26 — 1H NMR of polymer P6-3b in DMSO-d6 [file tjc-48-04-512s26.tif]

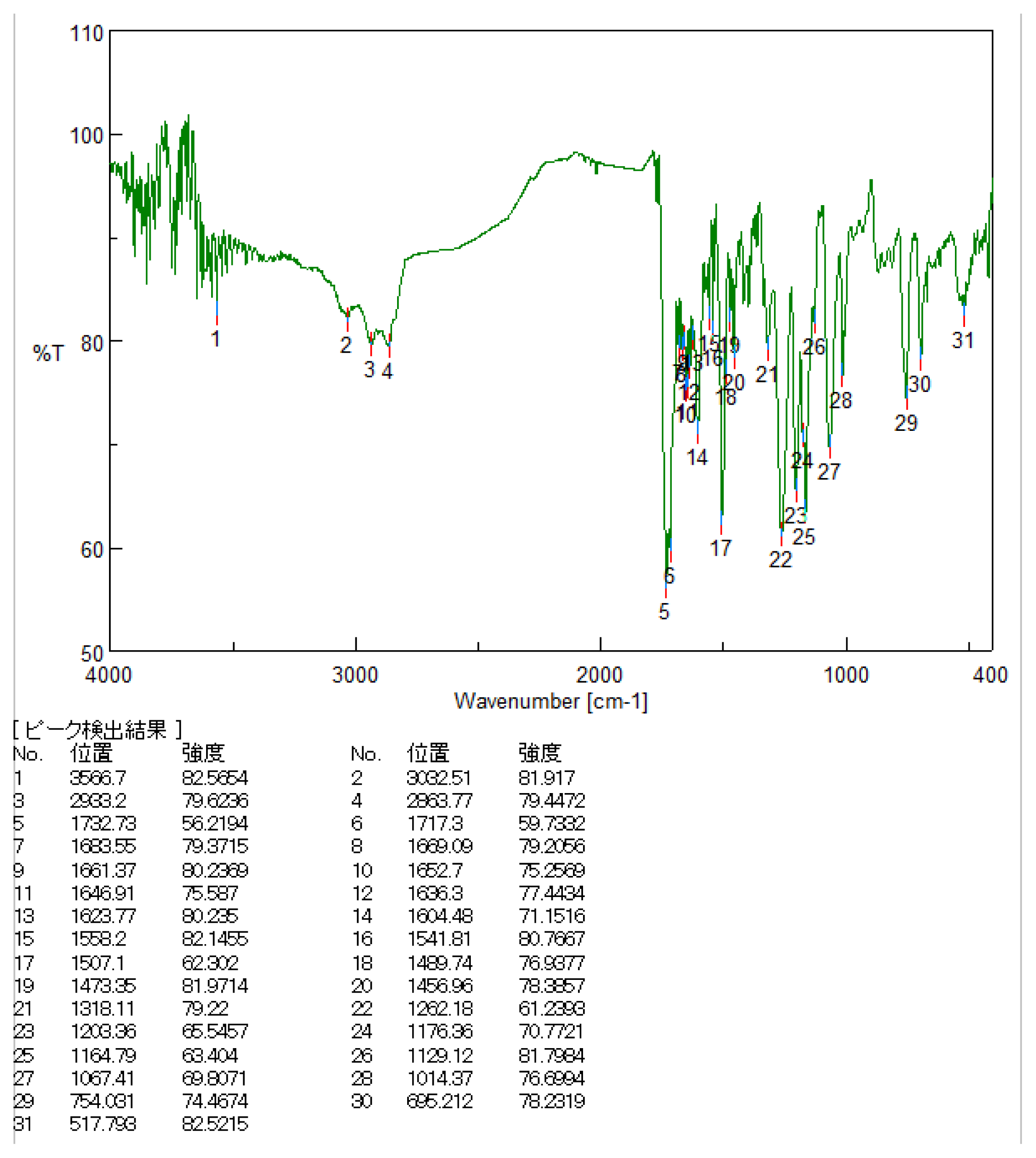

Supplement: Figure S27 — IR spectra of polymer P6-3b [file tjc-48-04-512s27.tif]

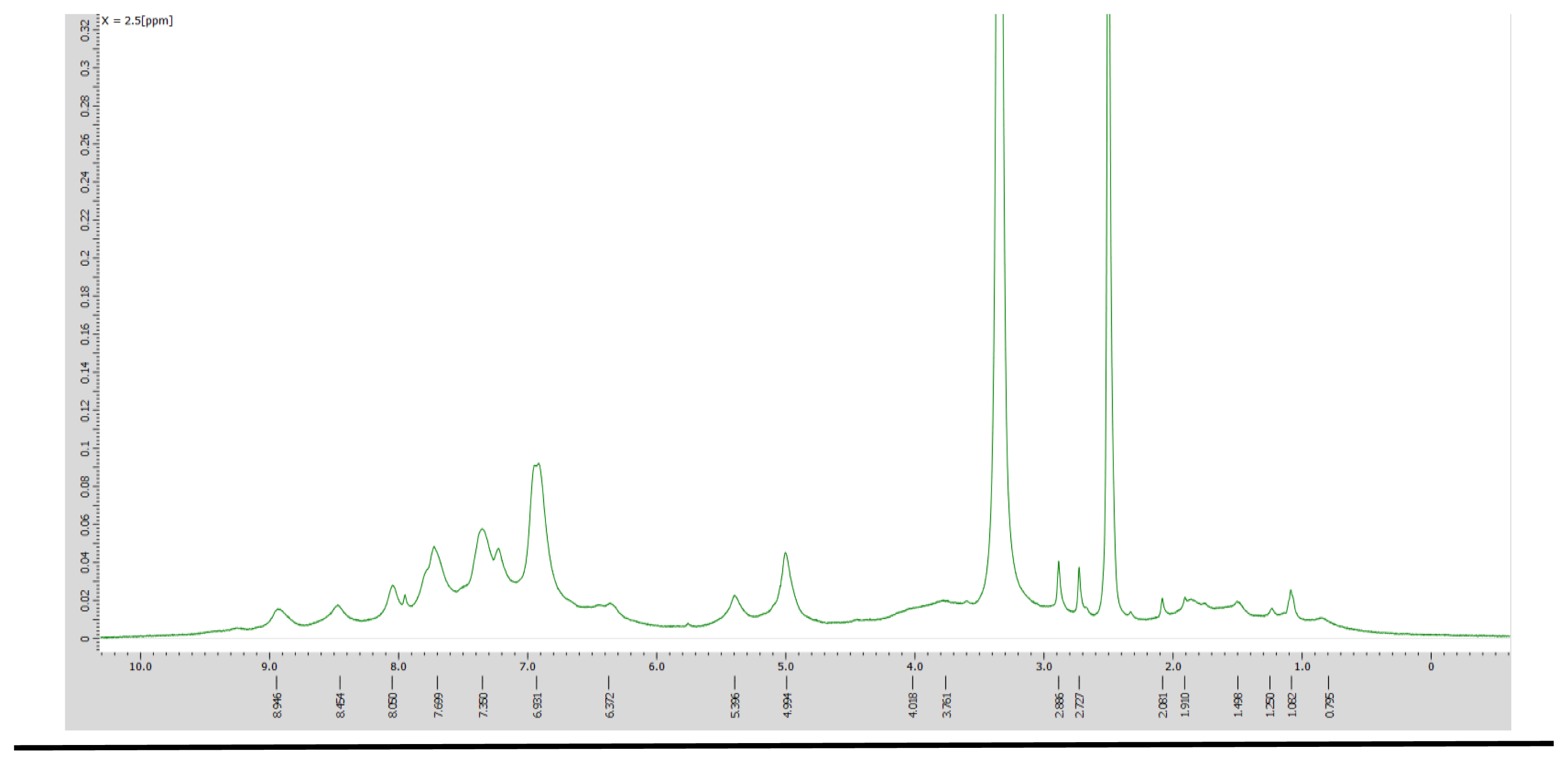

Supplement: Figure S28 — 1H NMR of polymer P7-3b in DMSO-d6 [file tjc-48-04-512s28.tif]

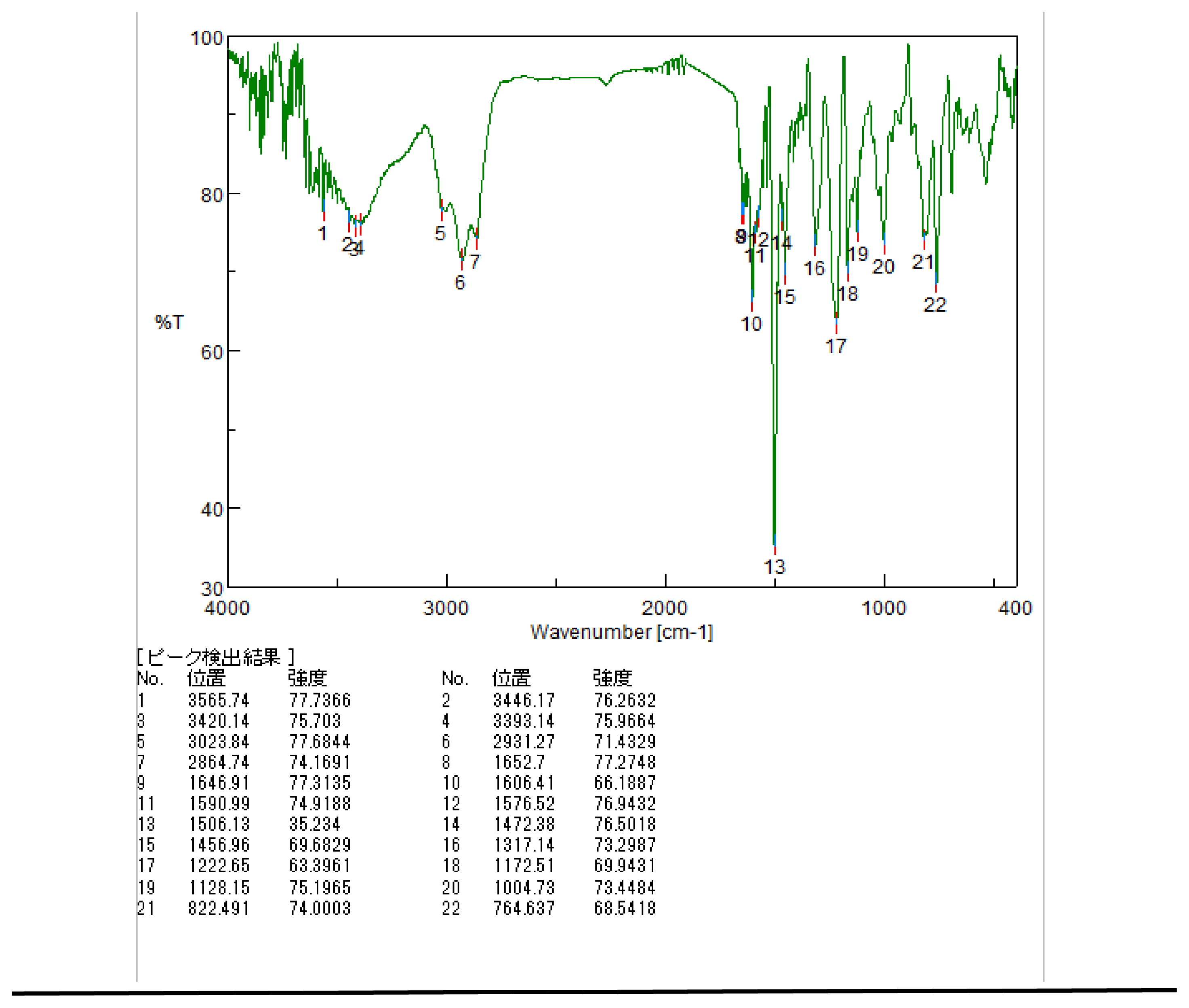

Supplement: Figure S29 — IR spectra of polymer P7-3b [file tjc-48-04-512s29.tif]

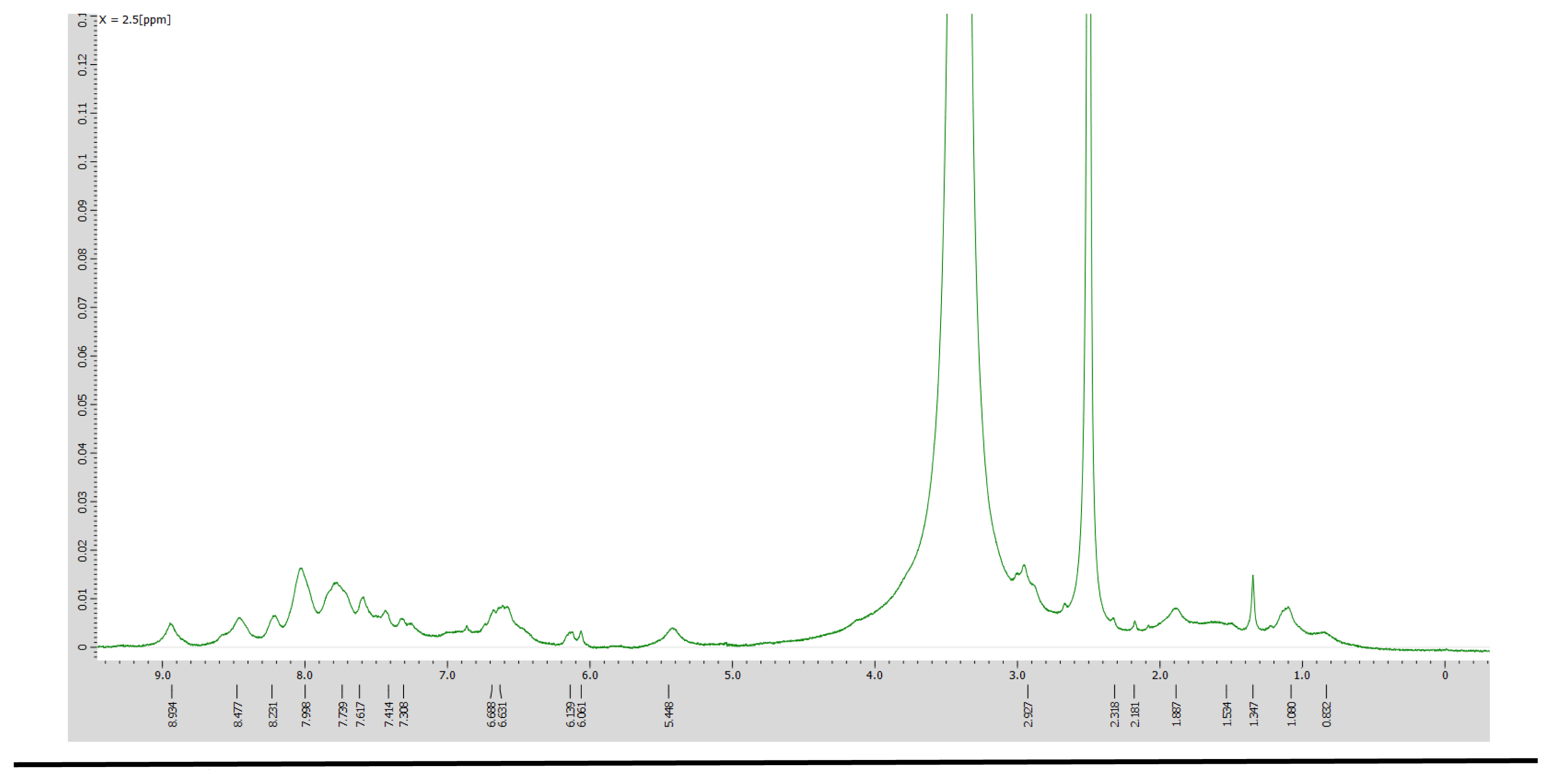

Supplement: Figure S30 — 1H NMR of polymer P8-6a in DMSO-d6 [file tjc-48-04-512s30.tif]

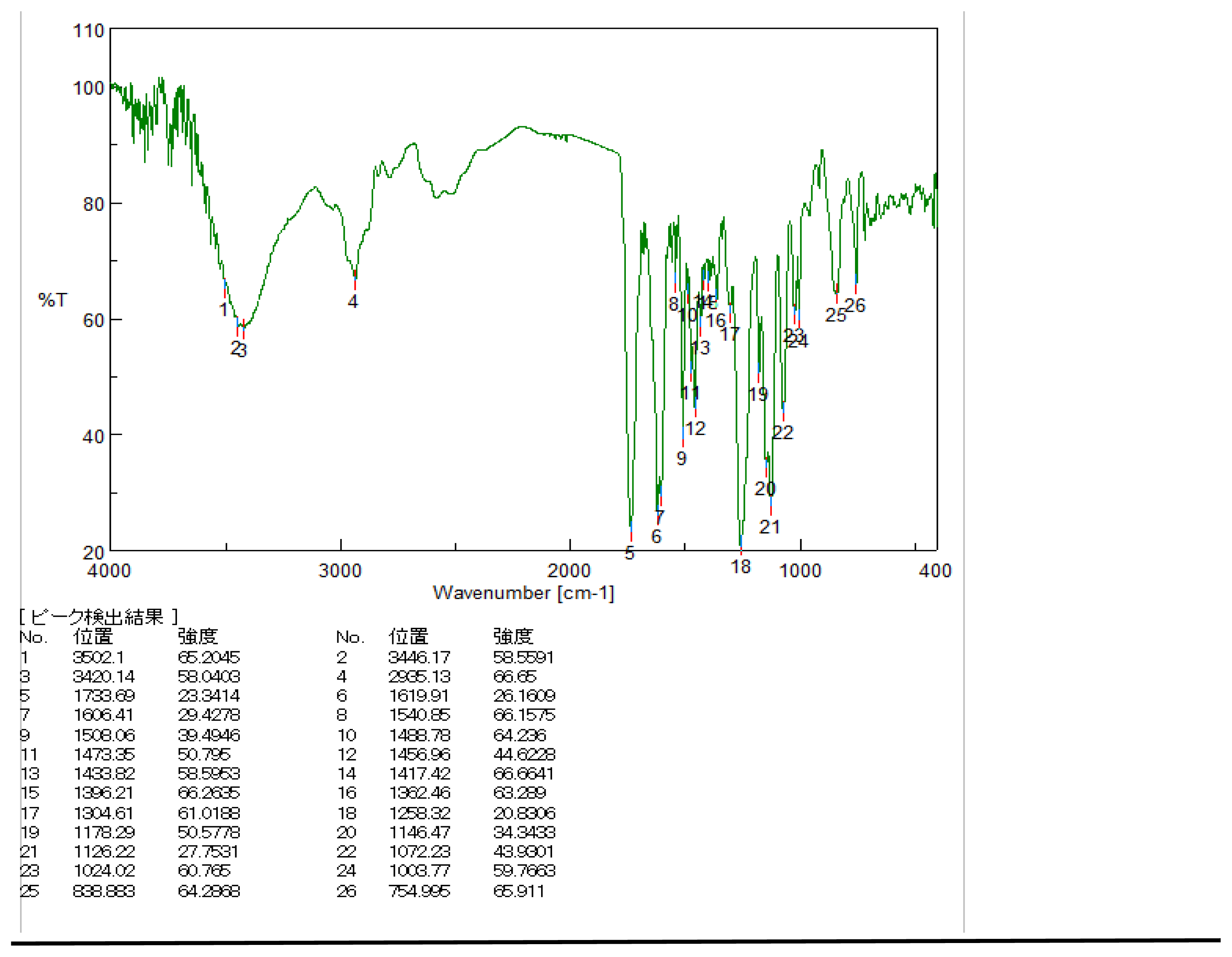

Supplement: Figure S31 — IR spectra of polymer P8-6a [file tjc-48-04-512s31.tif]

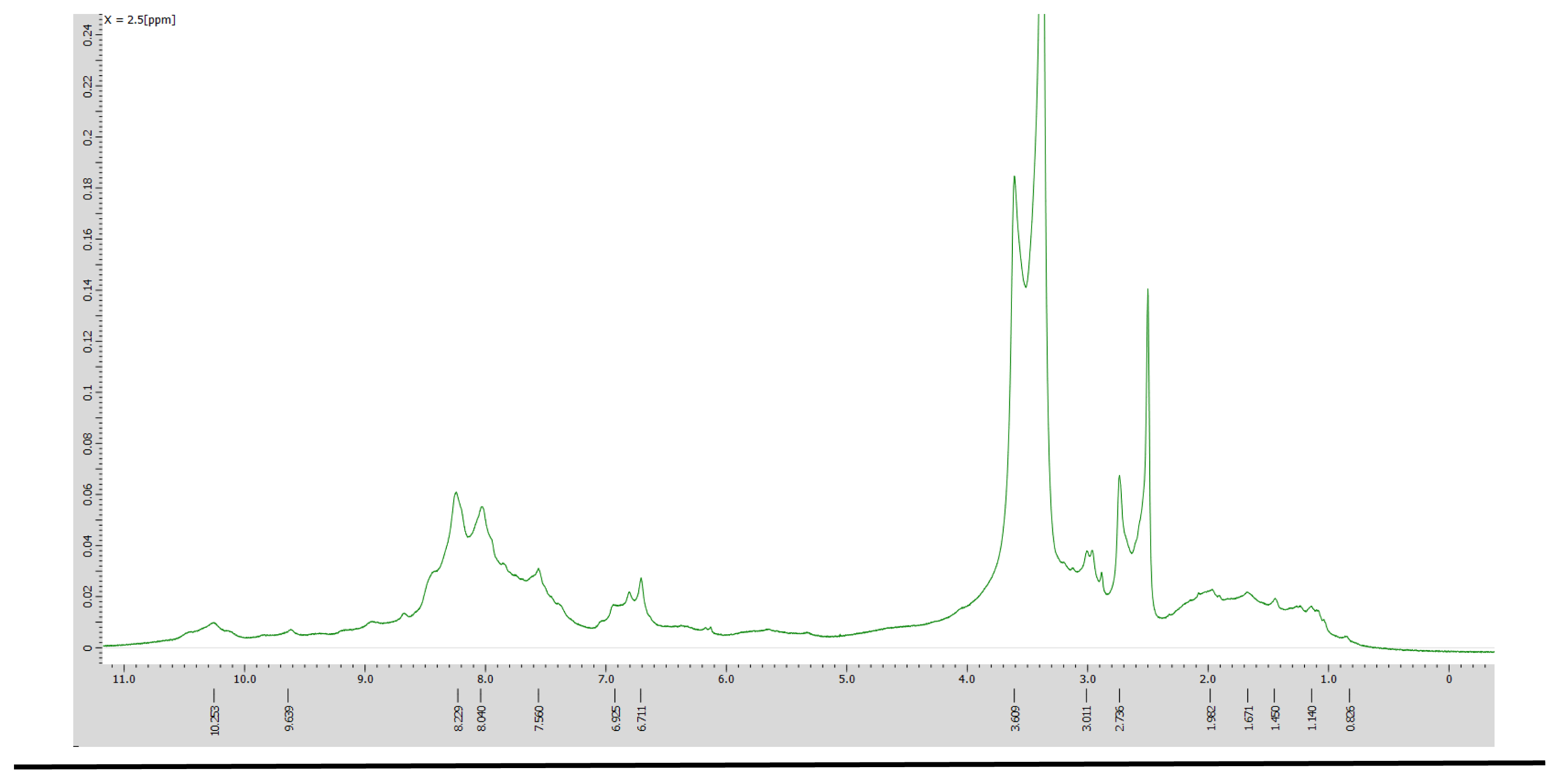

Supplement: Figure S32 — 1H NMR of polymer P9-6b in DMSO-d6 [file tjc-48-04-512s32.tif]

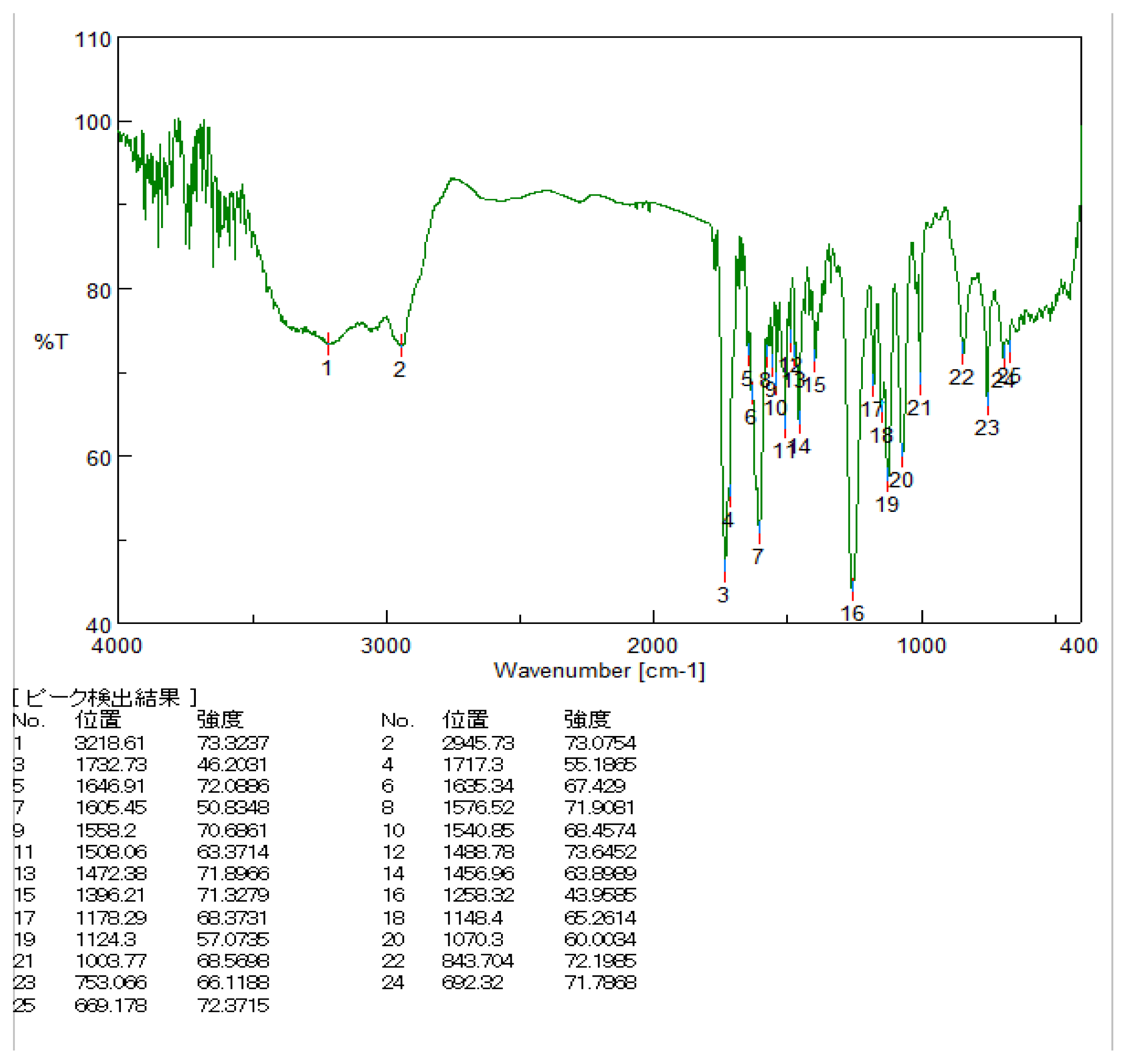

Supplement: Figure S33 — IR spectra of polymer P9-6b [file tjc-48-04-512s33.tif]

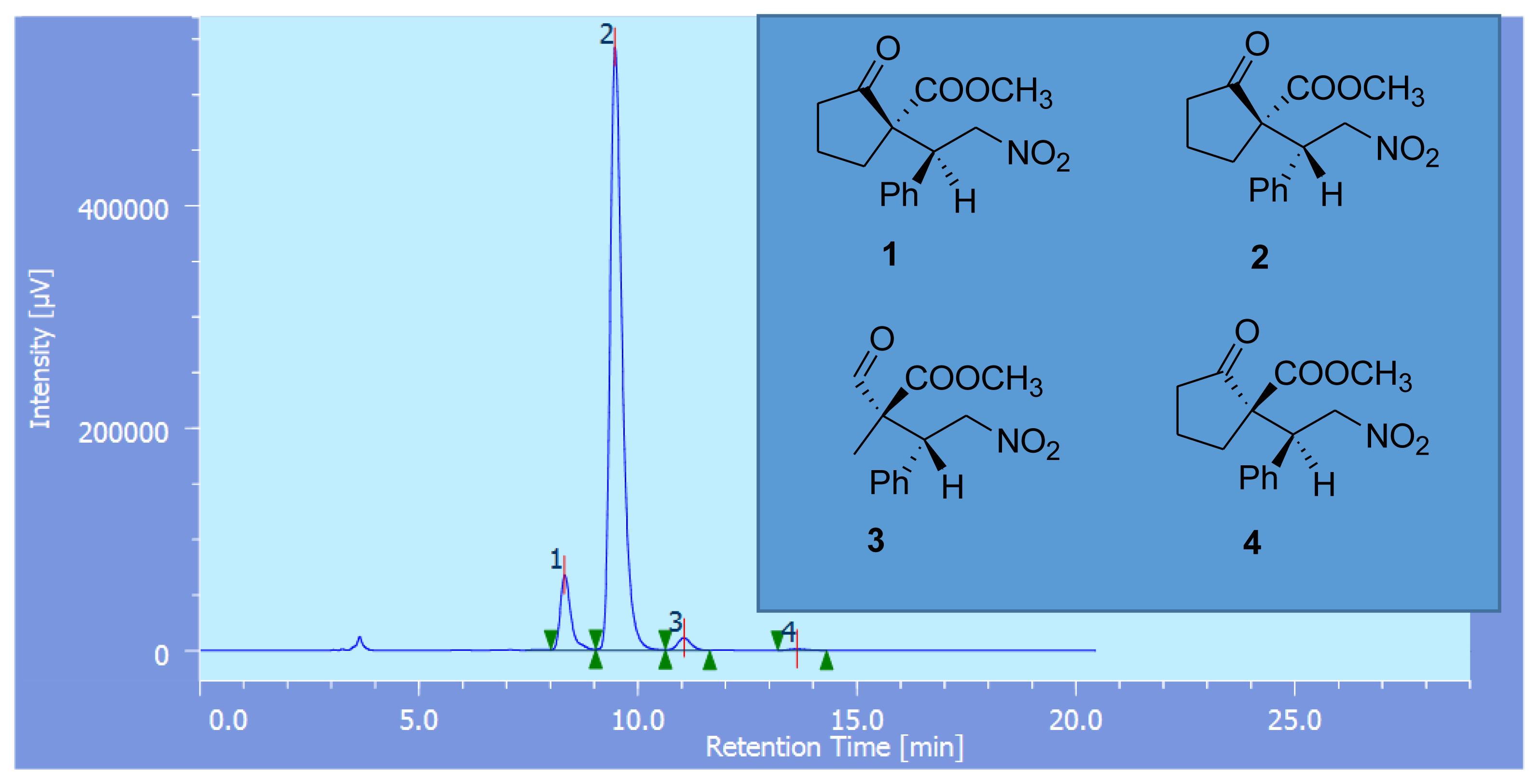

Supplement: Figure S34 — HPLC chromatogram of asymmetric compound, 13 Table 2, entry 1 99% ee [file tjc-48-04-512s34.tif]

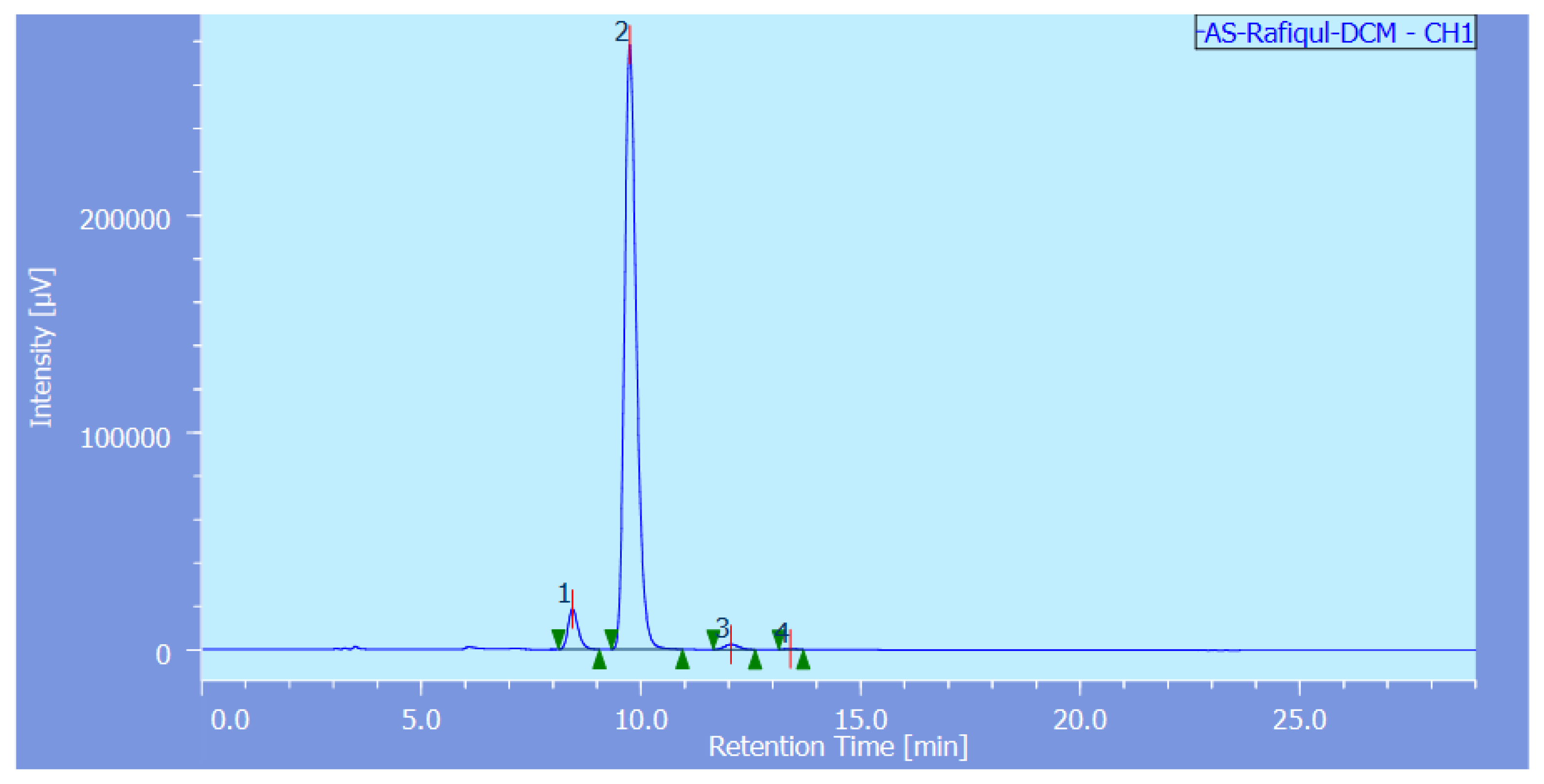

Supplement: Figure S35 — HPLC chromatogram of asymmetric compound, 13 Table 2, entry 2 99% ee [file tjc-48-04-512s35.tif]

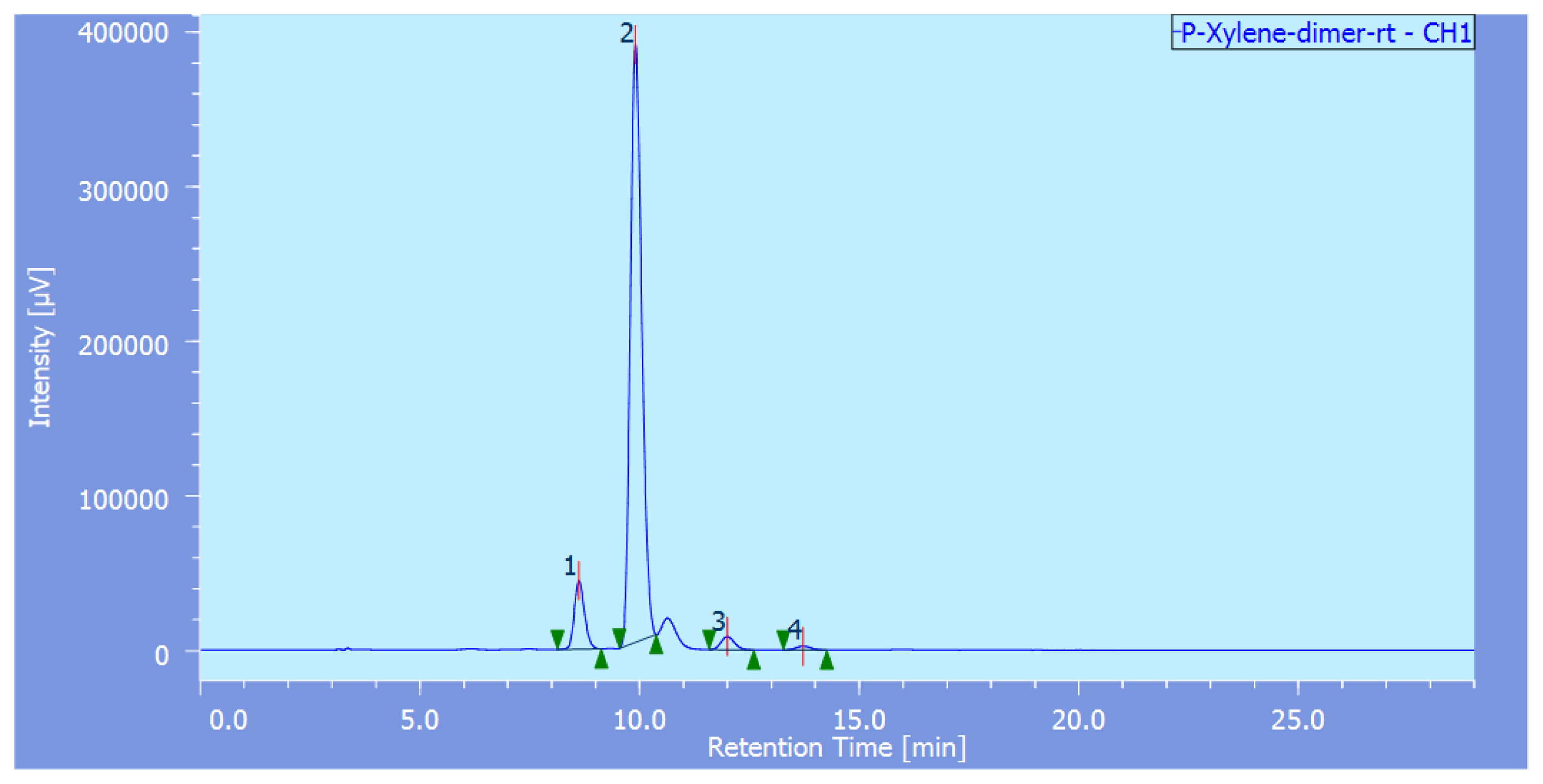

Supplement: Figure S36 — HPLC chromatogram of asymmetric compound, 13 Table 2, entry 3 98% ee [file tjc-48-04-512s36.tif]

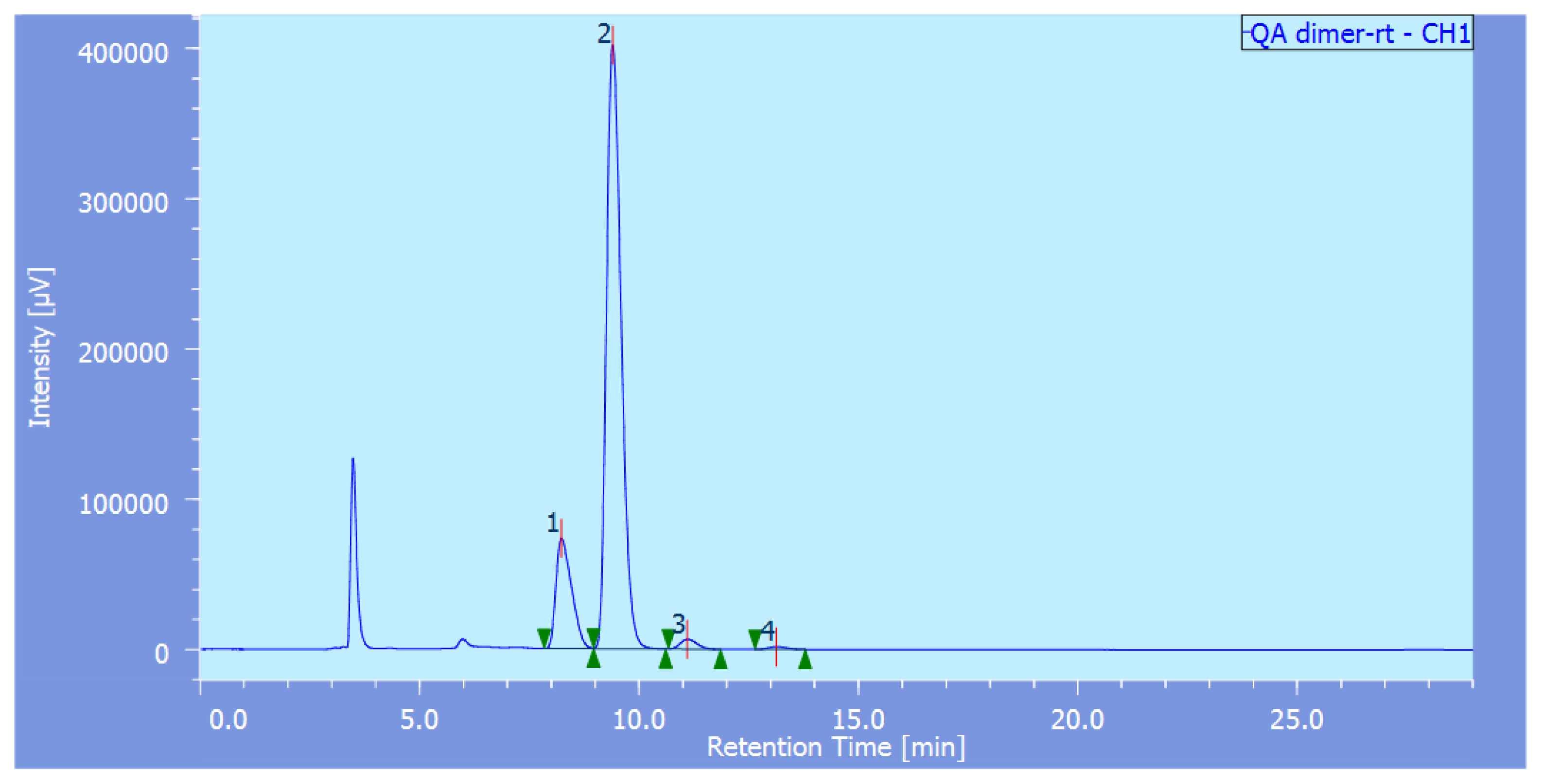

Supplement: Figure S37 — HPLC chromatogram of asymmetric compound, 13 Table 2, entry 4 99% ee [file tjc-48-04-512s37.tif]

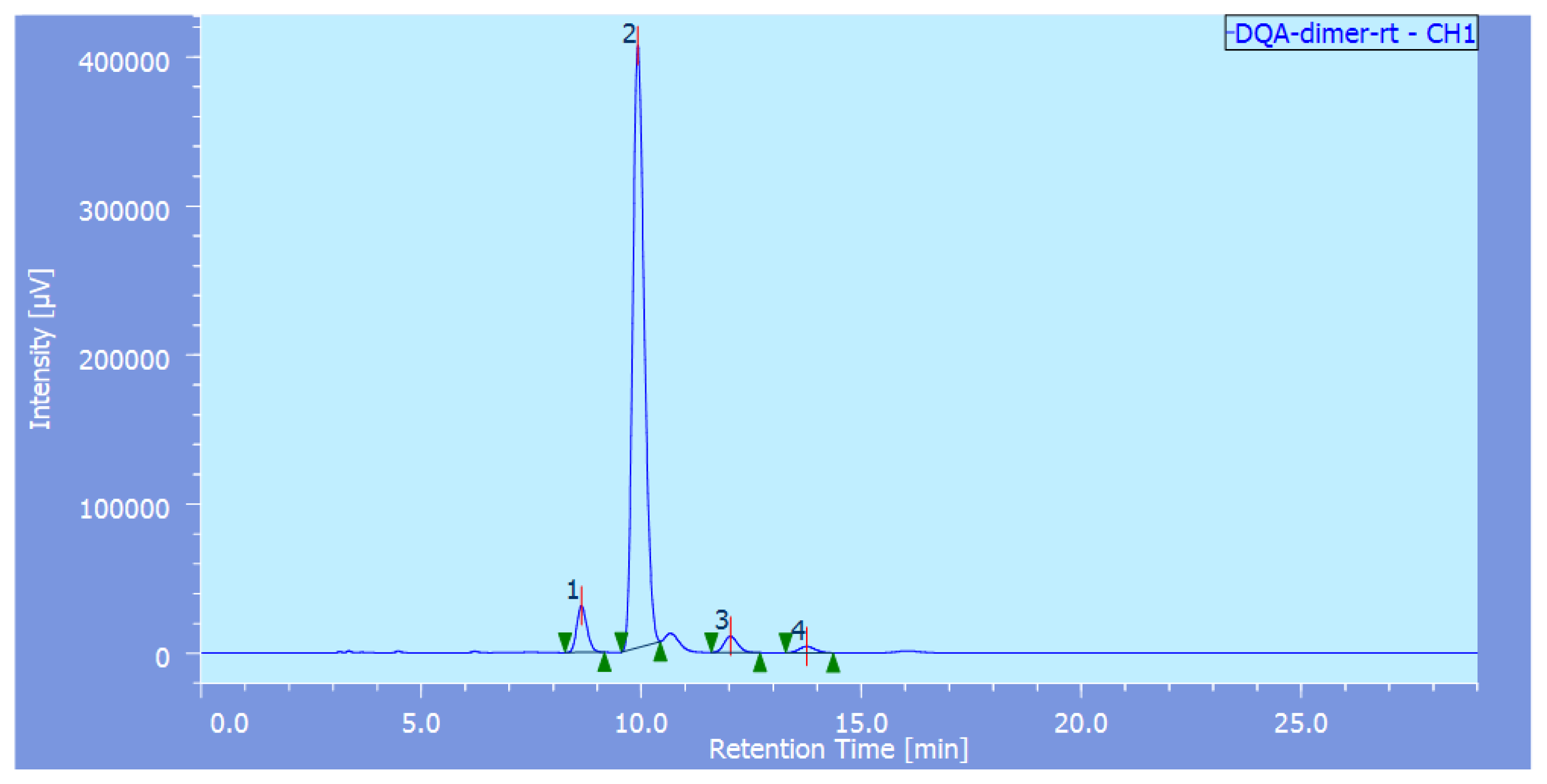

Supplement: Figure S38 — HPLC chromatogram of asymmetric compound, 13 Table 2, entry 5 97% ee [file tjc-48-04-512s38.tif]

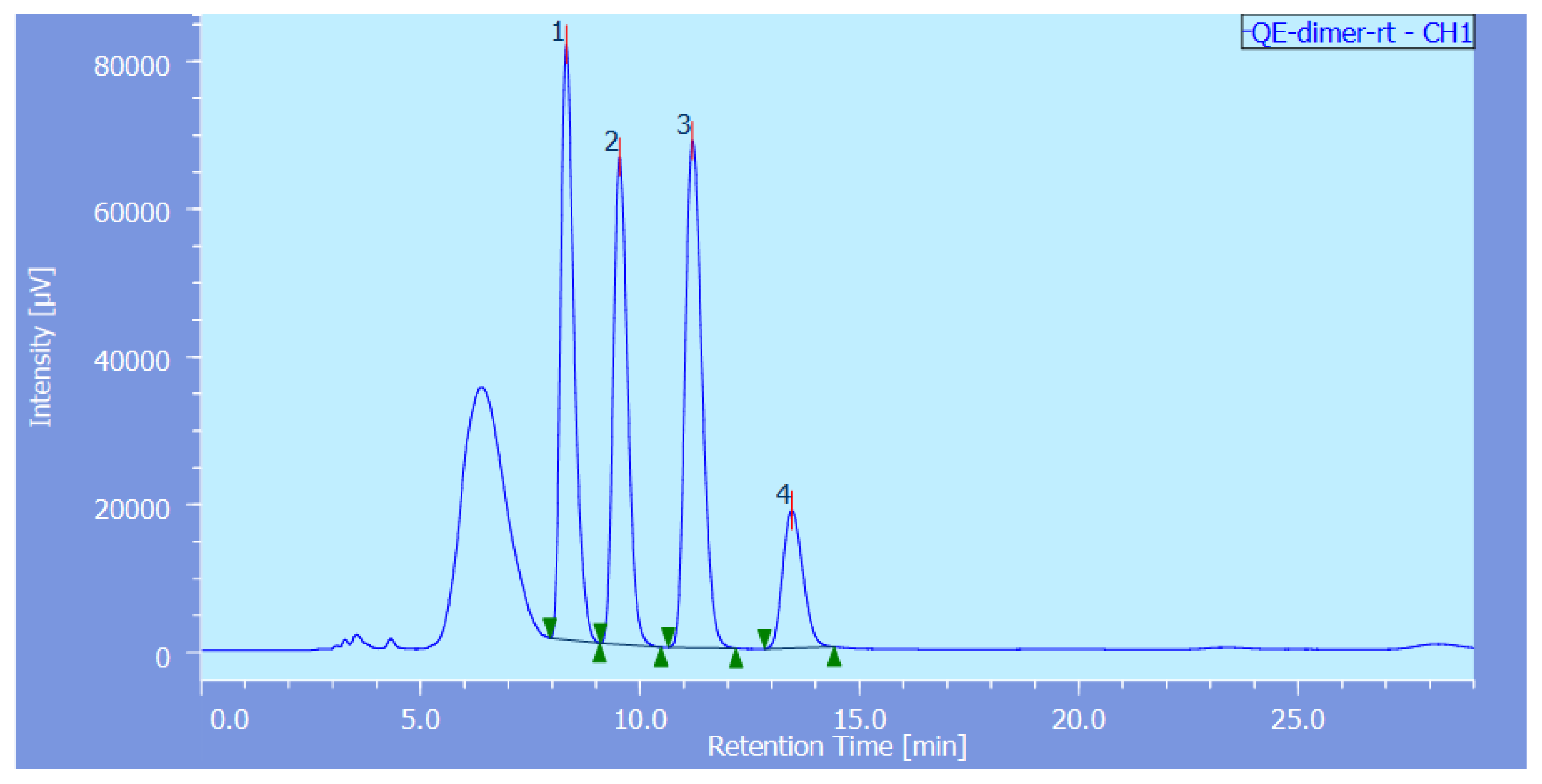

Supplement: Figure S39 — HPLC chromatogram of asymmetric compound, 13 Table 2, entry 6 44% ee [file tjc-48-04-512s39.tif]

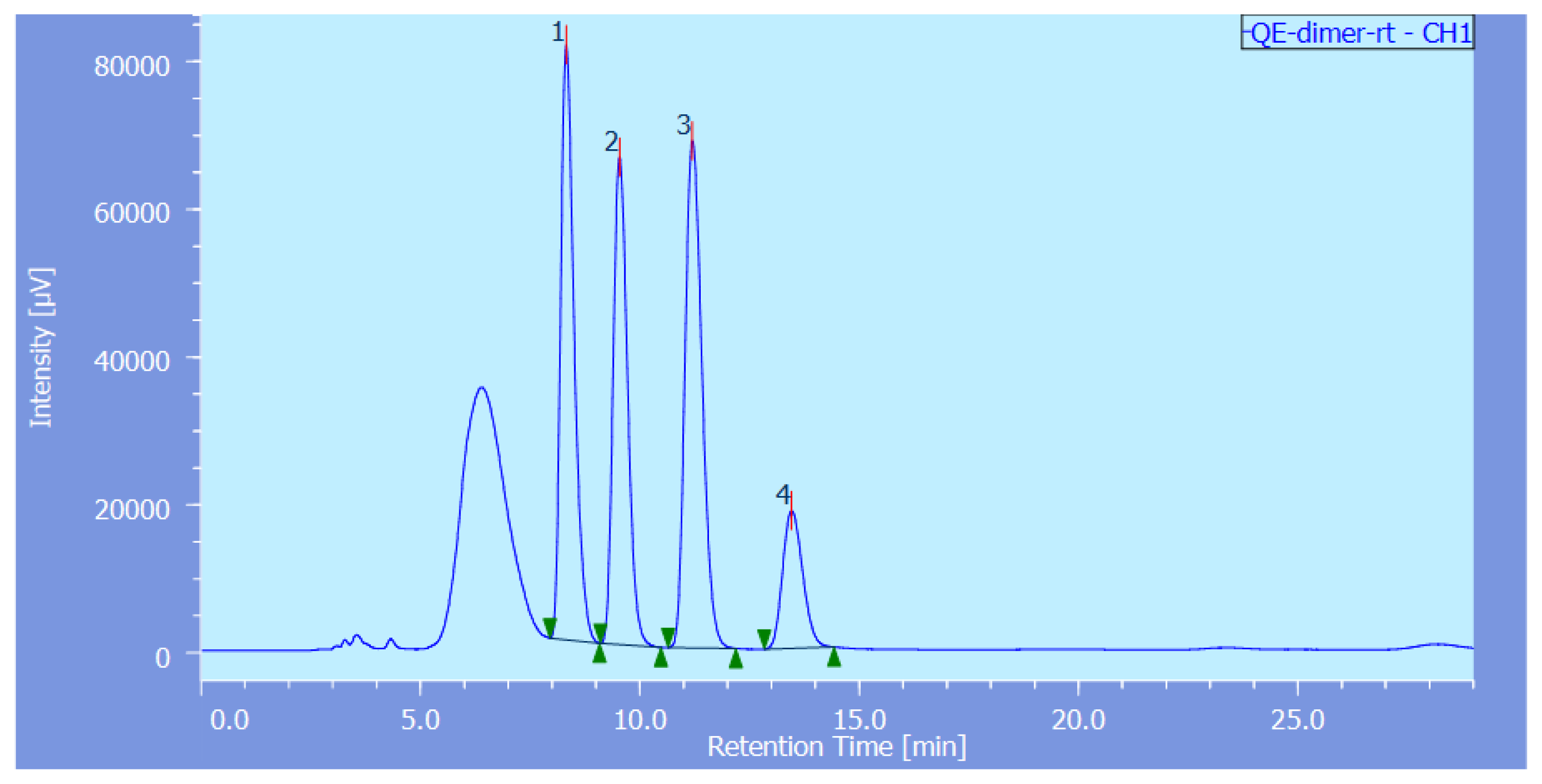

Supplement: Figure S40 — HPLC chromatogram of asymmetric compound, 13 Table 2, entry 7 99% ee [file tjc-48-04-512s40.tif]

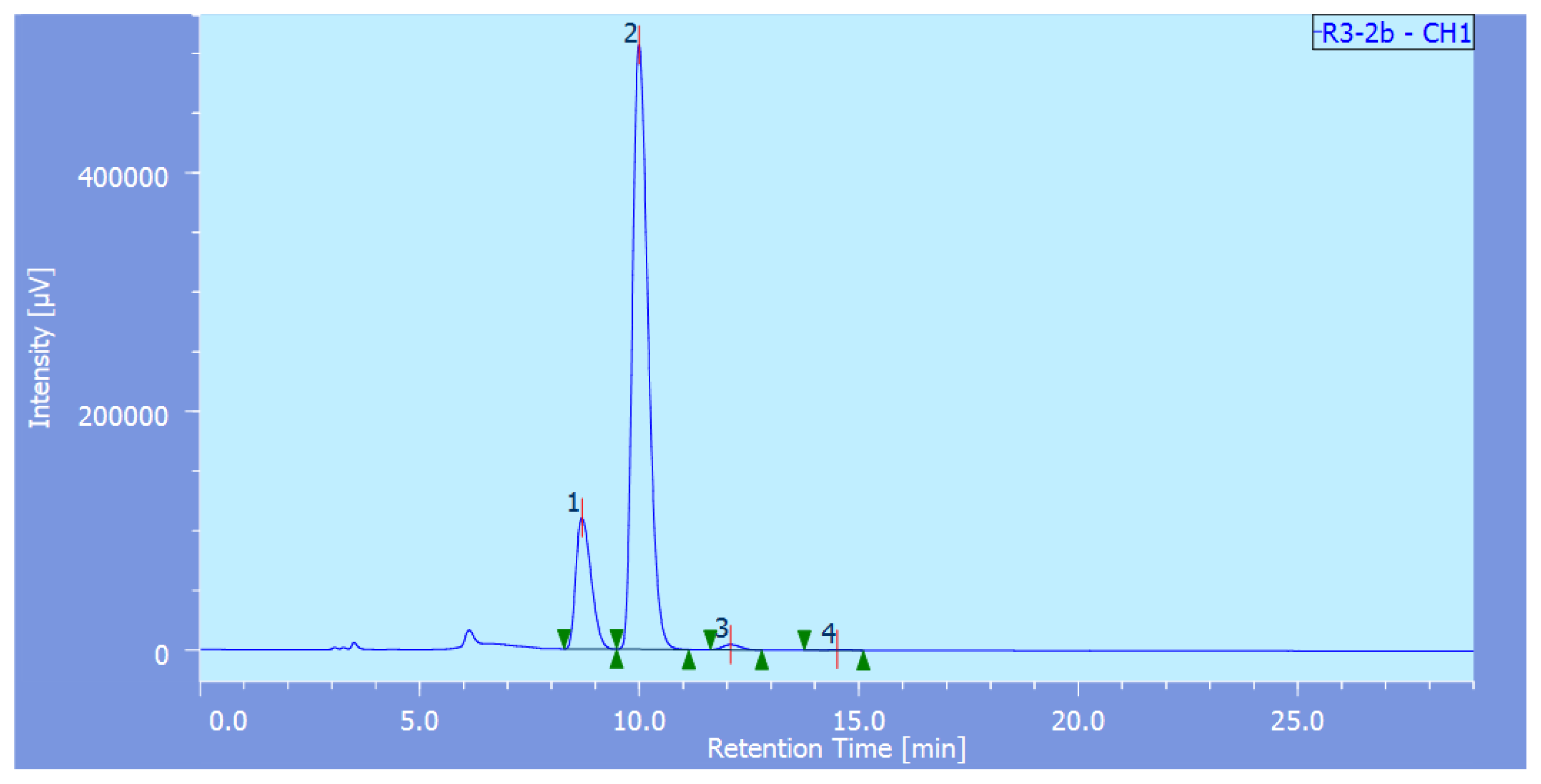

Supplement: Figure S41 — HPLC chromatogram of asymmetric compound, 13 Table 3, entry 1 99% ee [file tjc-48-04-512s41.tif]

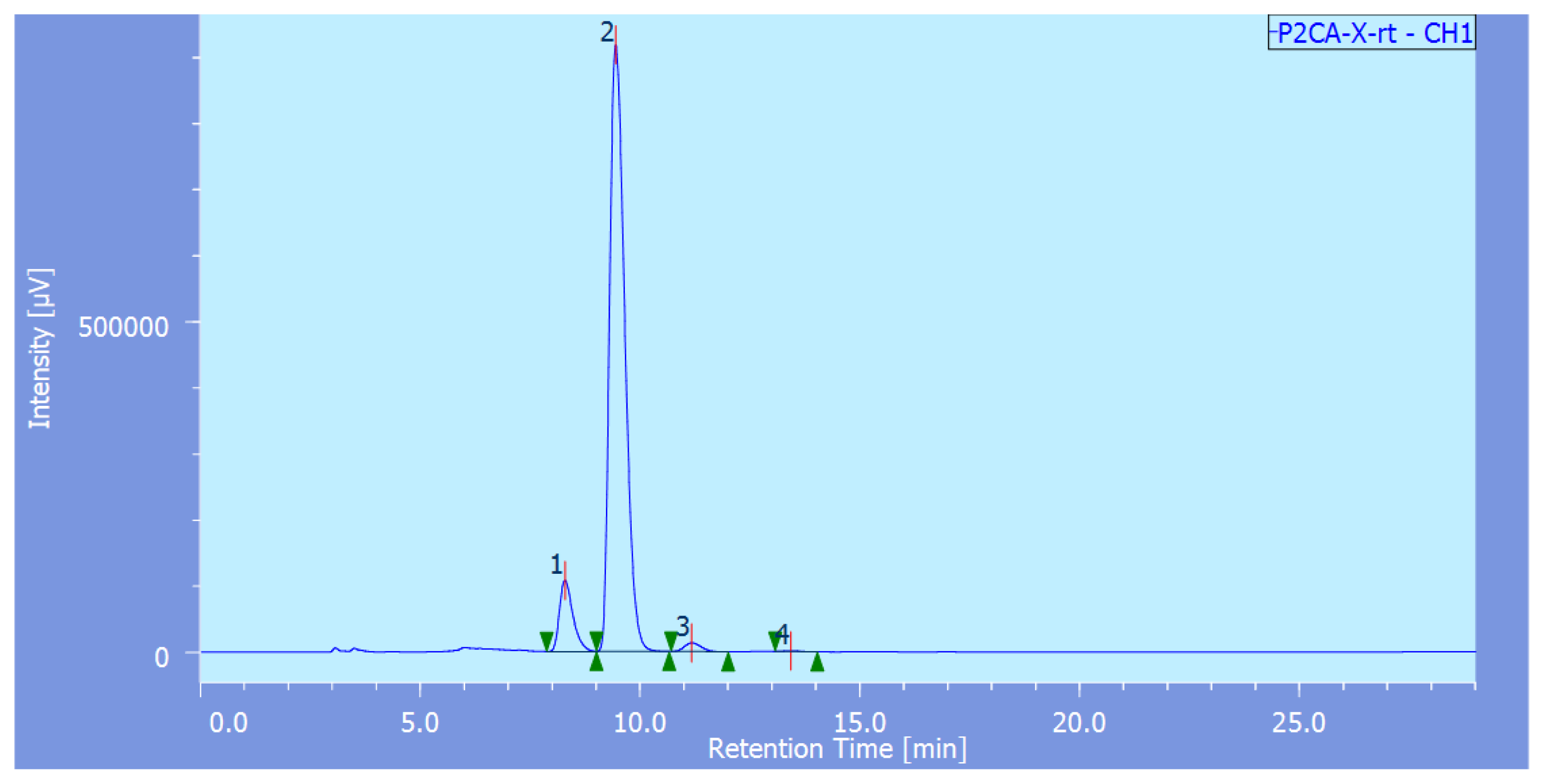

Supplement: Figure S42 — HPLC chromatogram of asymmetric compound, 13 Table 3, entry 2 >99% ee [file tjc-48-04-512s42.tif]

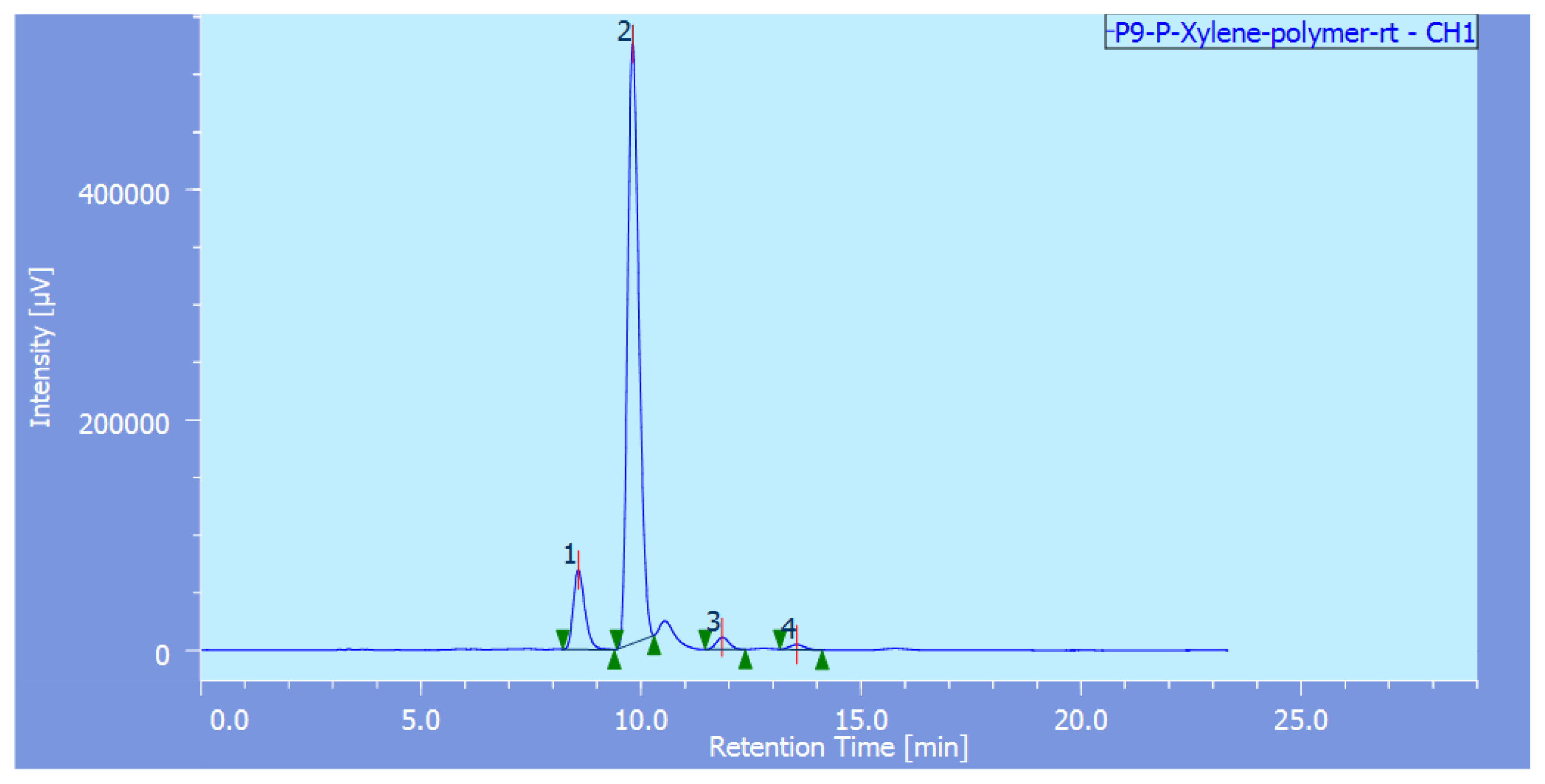

Supplement: Figure S43 — HPLC chromatogram of asymmetric compound, 13 Table 3, entry 3 98% ee [file tjc-48-04-512s43.tif]

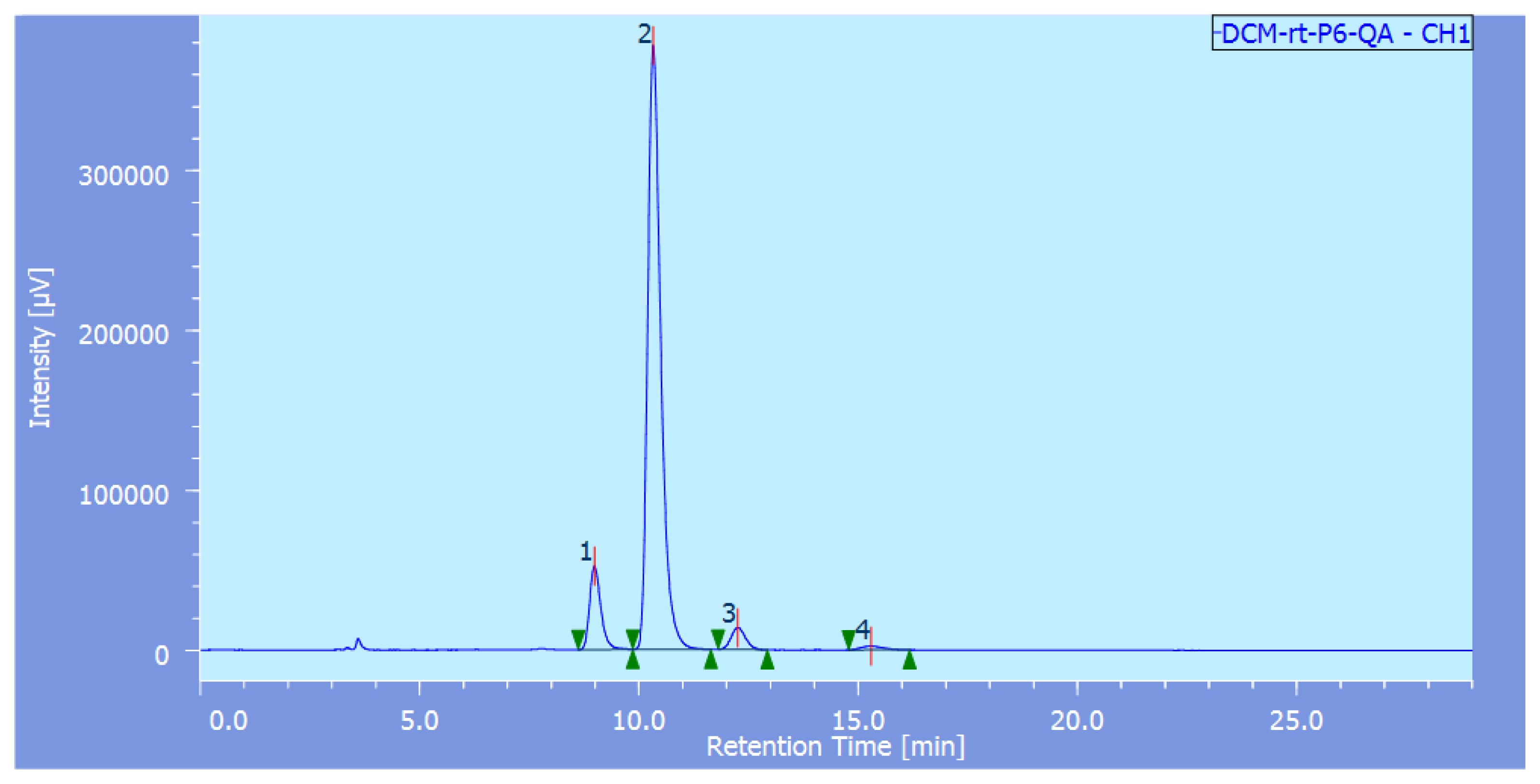

Supplement: Figure S44 — HPLC chromatogram of asymmetric compound, 13 Table 3, entry 4 98% ee [file tjc-48-04-512s44.tif]

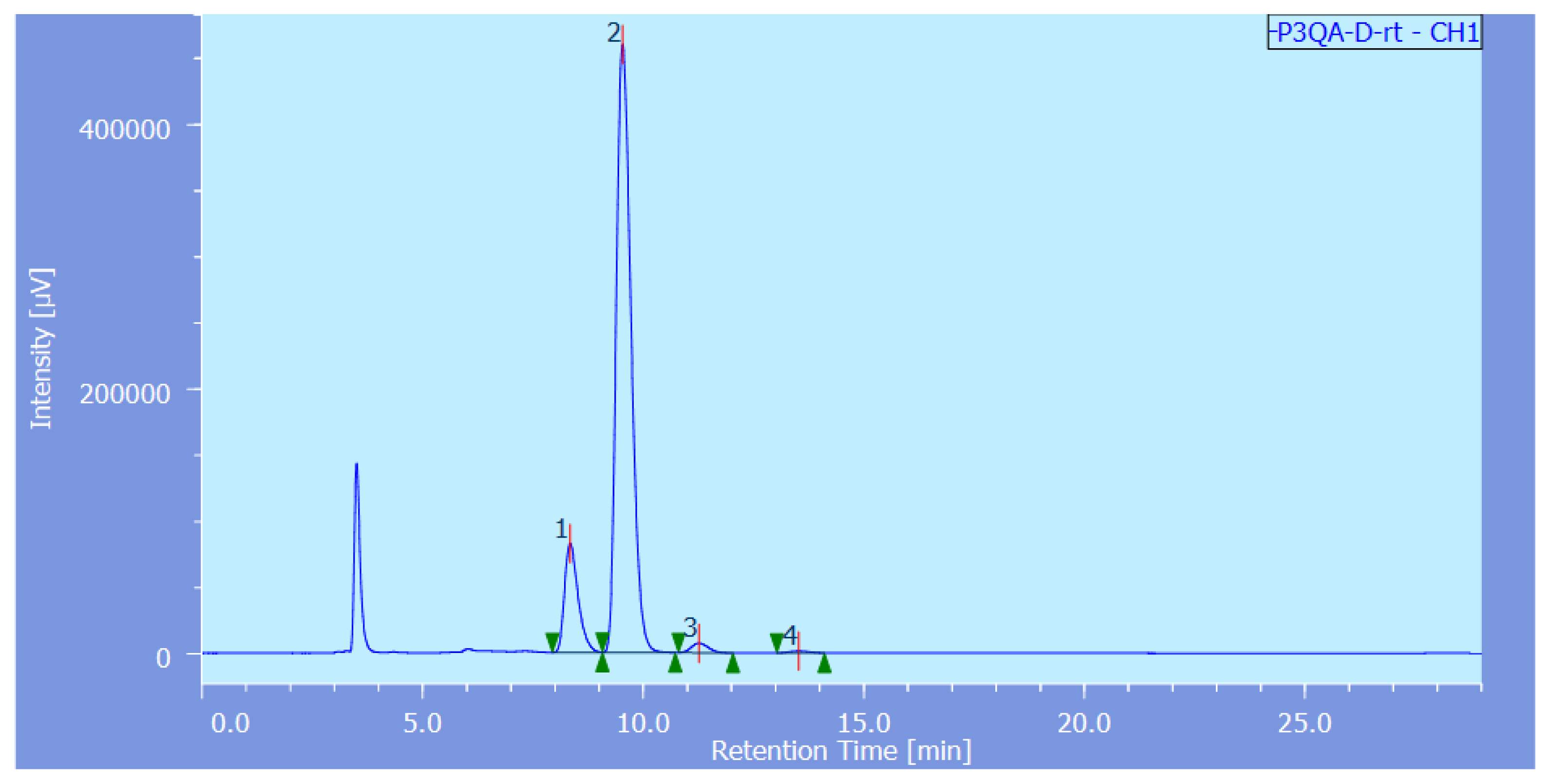

Supplement: Figure S45 — HPLC chromatogram of asymmetric compound, 13 Table 3, entry 5 99% ee [file tjc-48-04-512s45.tif]

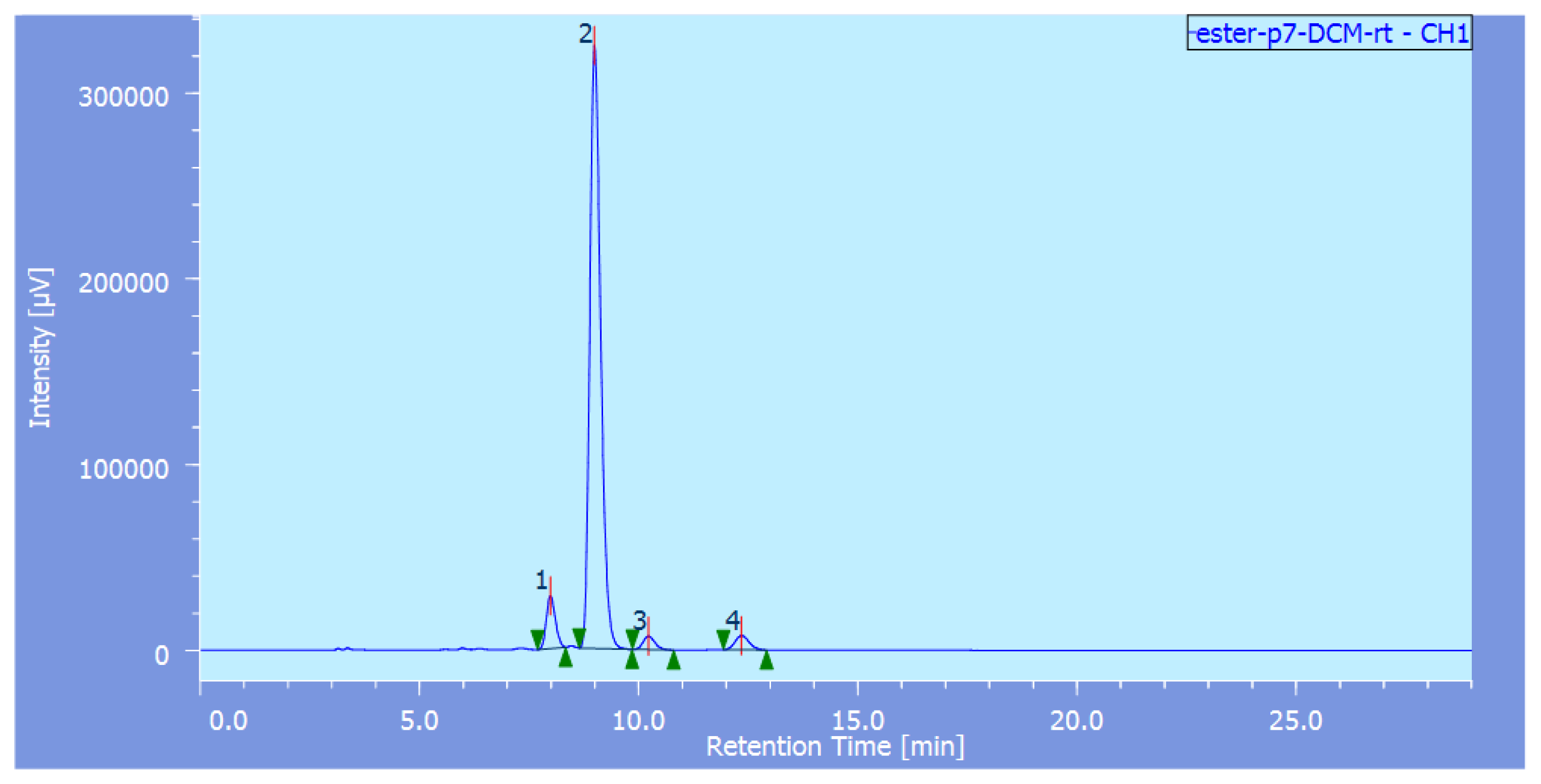

Supplement: Figure S46 — HPLC chromatogram of asymmetric compound, 13 Table 3, entry 6 94% ee [file tjc-48-04-512s46.tif]

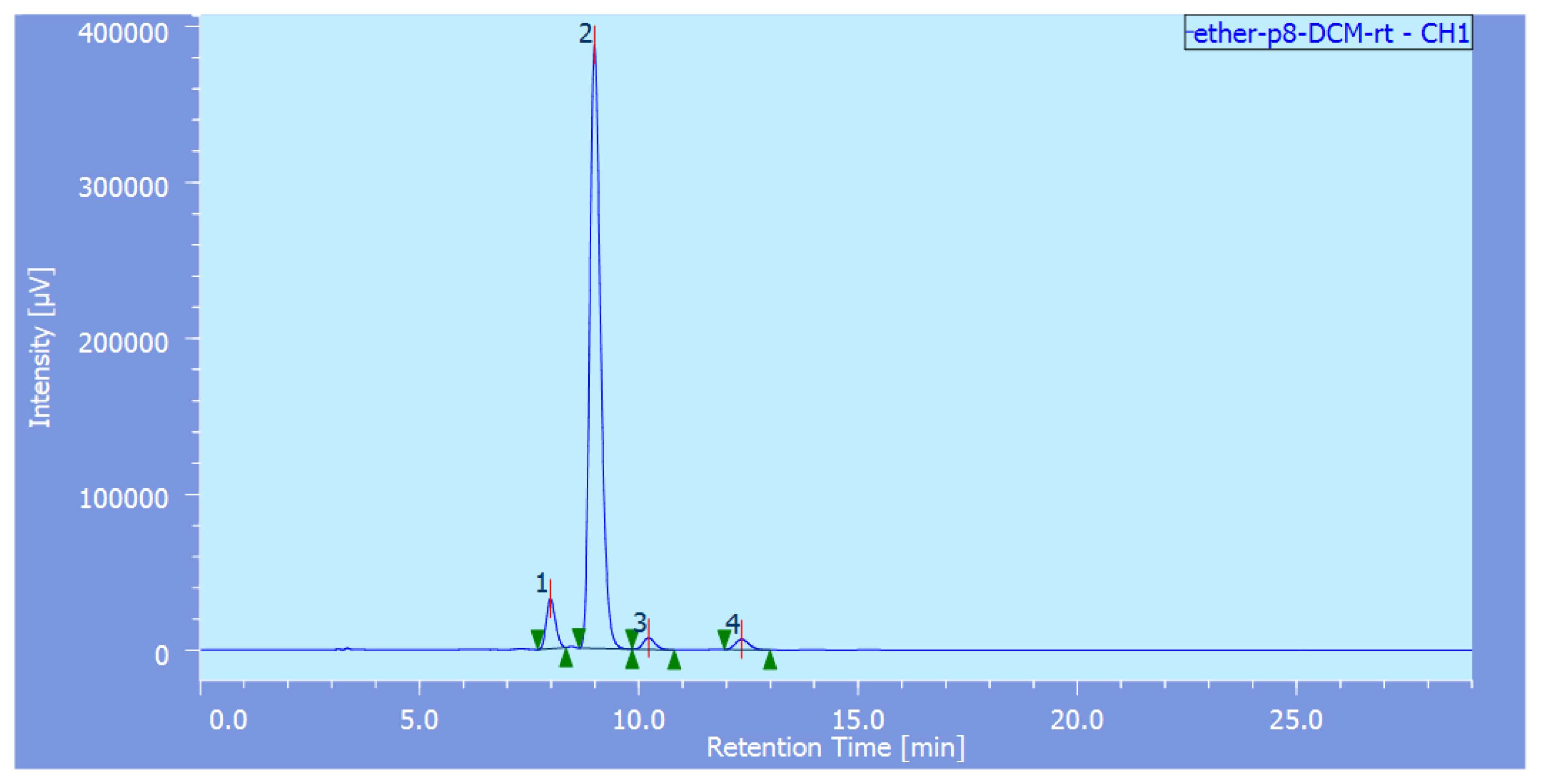

Supplement: Figure S47 — HPLC chromatogram of asymmetric compound, 13 Table 3, entry 7 96% ee [file tjc-48-04-512s47.tif]

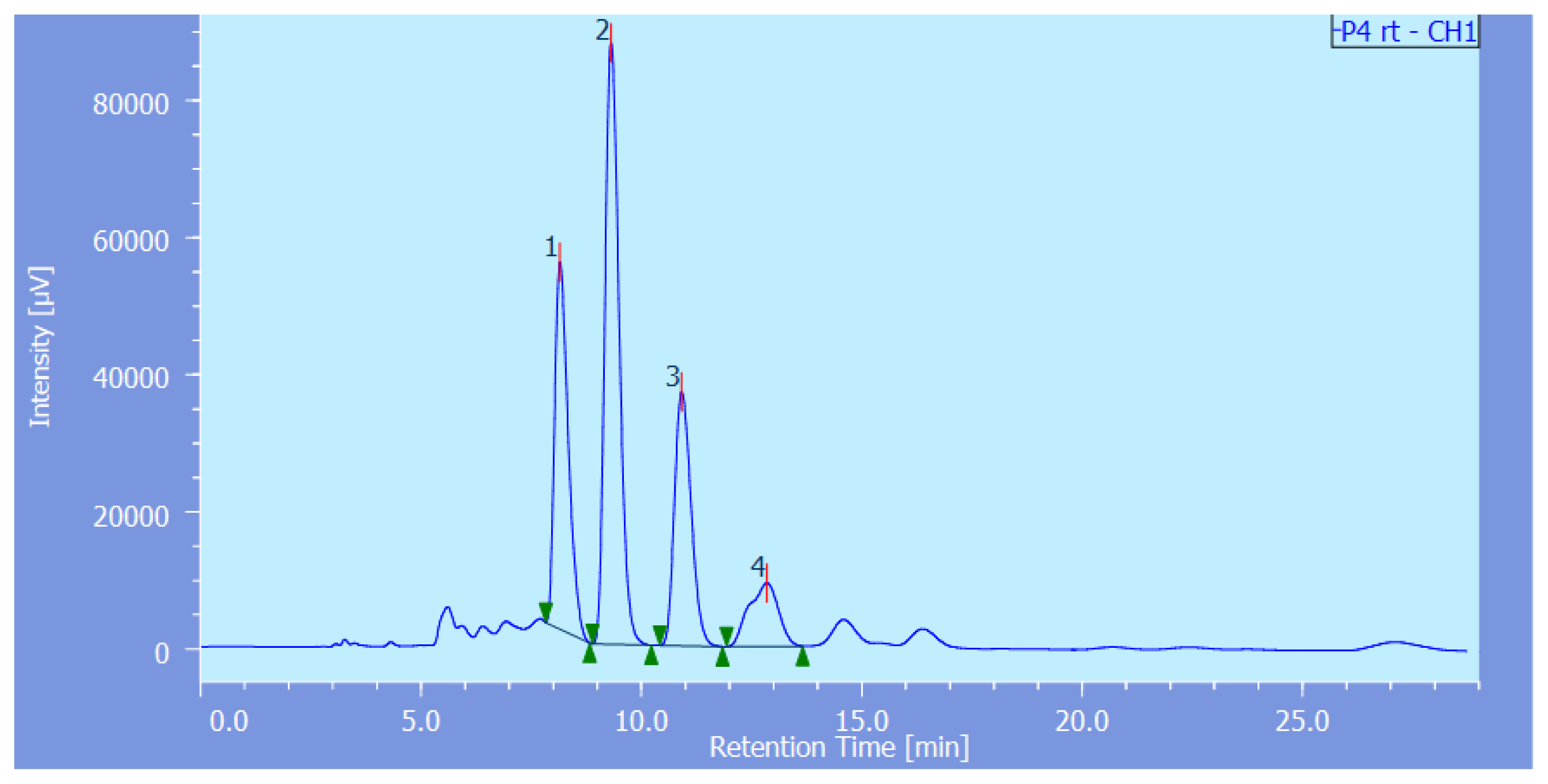

Supplement: Figure S48 — HPLC chromatogram of asymmetric compound, 13 Table 3, entry 8 64% ee [file tjc-48-04-512s48.tif]

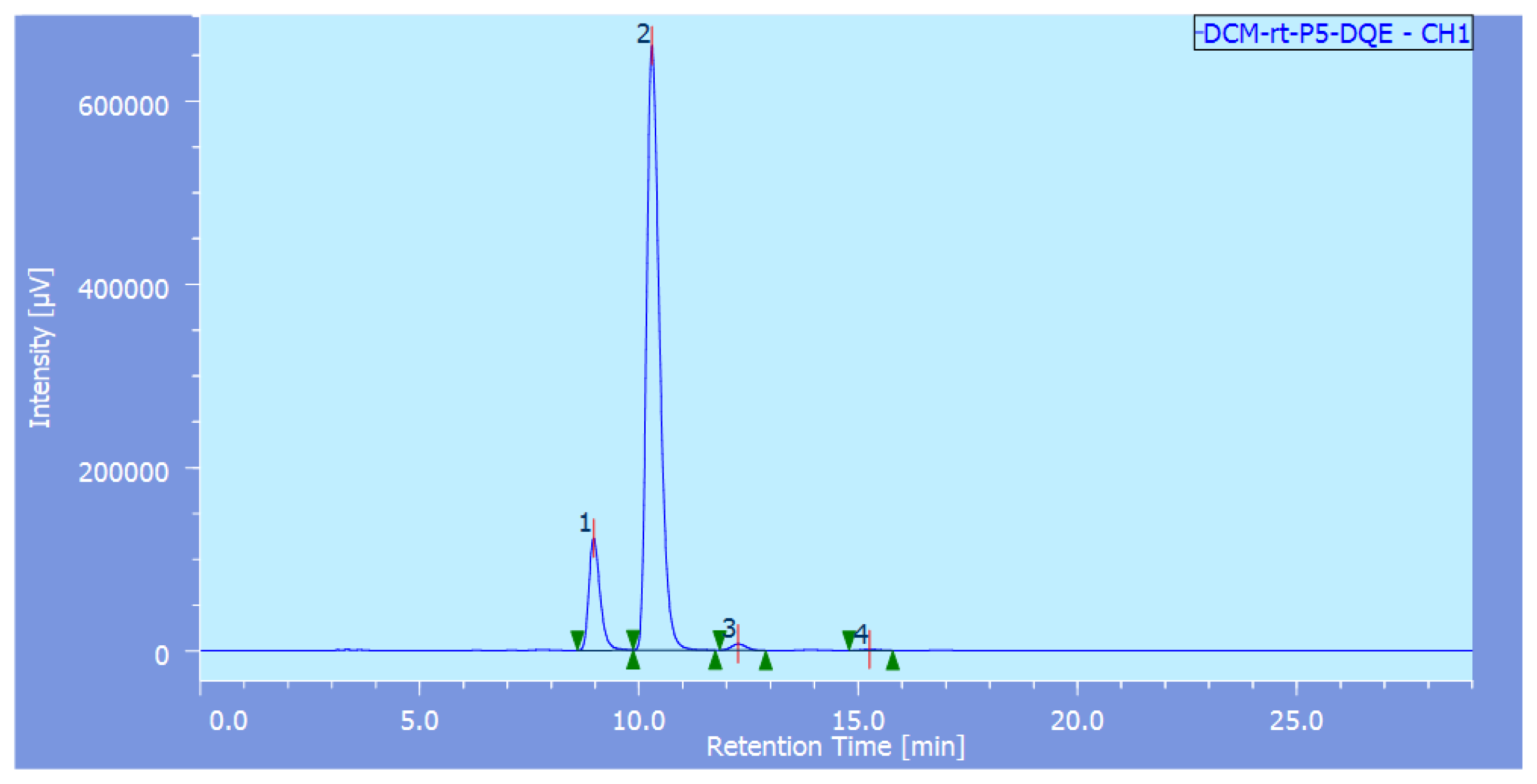

Supplement: Figure S49 — HPLC chromatogram of asymmetric compound, 13 Table 3, entry 9 >99% ee [file tjc-48-04-512s49.tif]

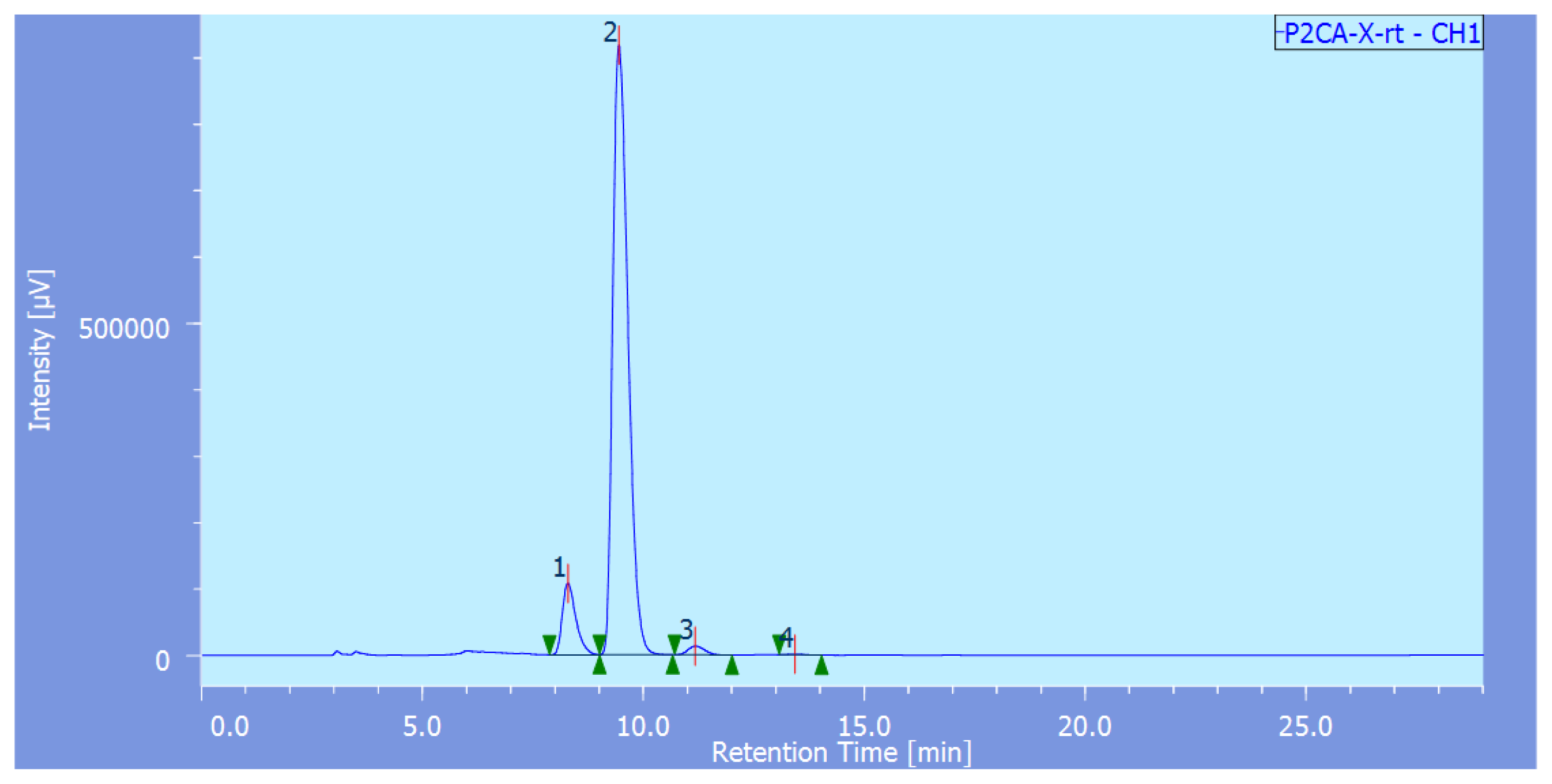

Supplement: Figure S50 — HPLC chromatogram of asymmetric compound, 13 Table 4, entry 1 >99% ee [file tjc-48-04-512s50.tif]

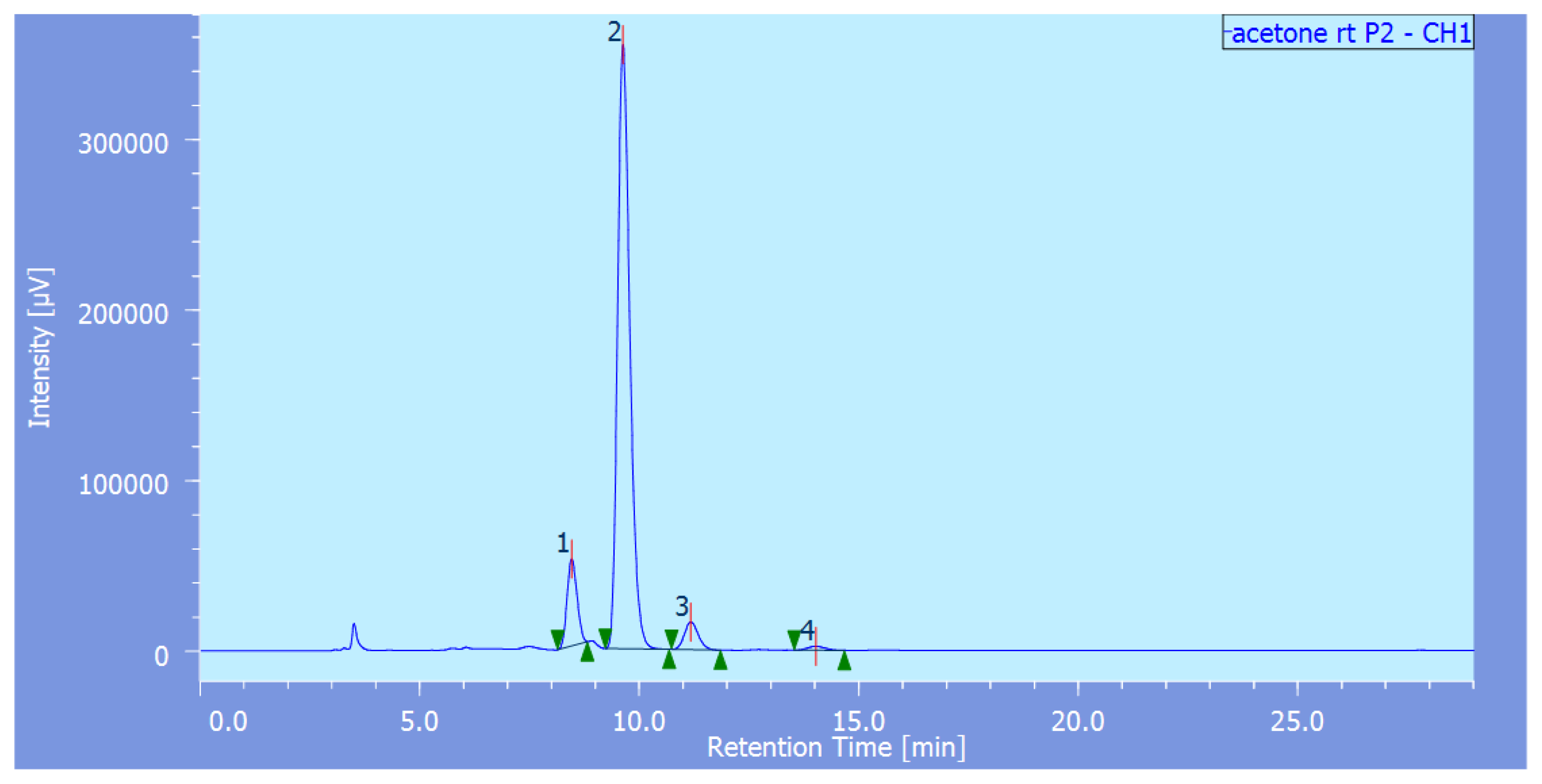

Supplement: Figure S51 — HPLC chromatogram of asymmetric compound, 13 Table 4, entry 2 98% ee [file tjc-48-04-512s51.tif]

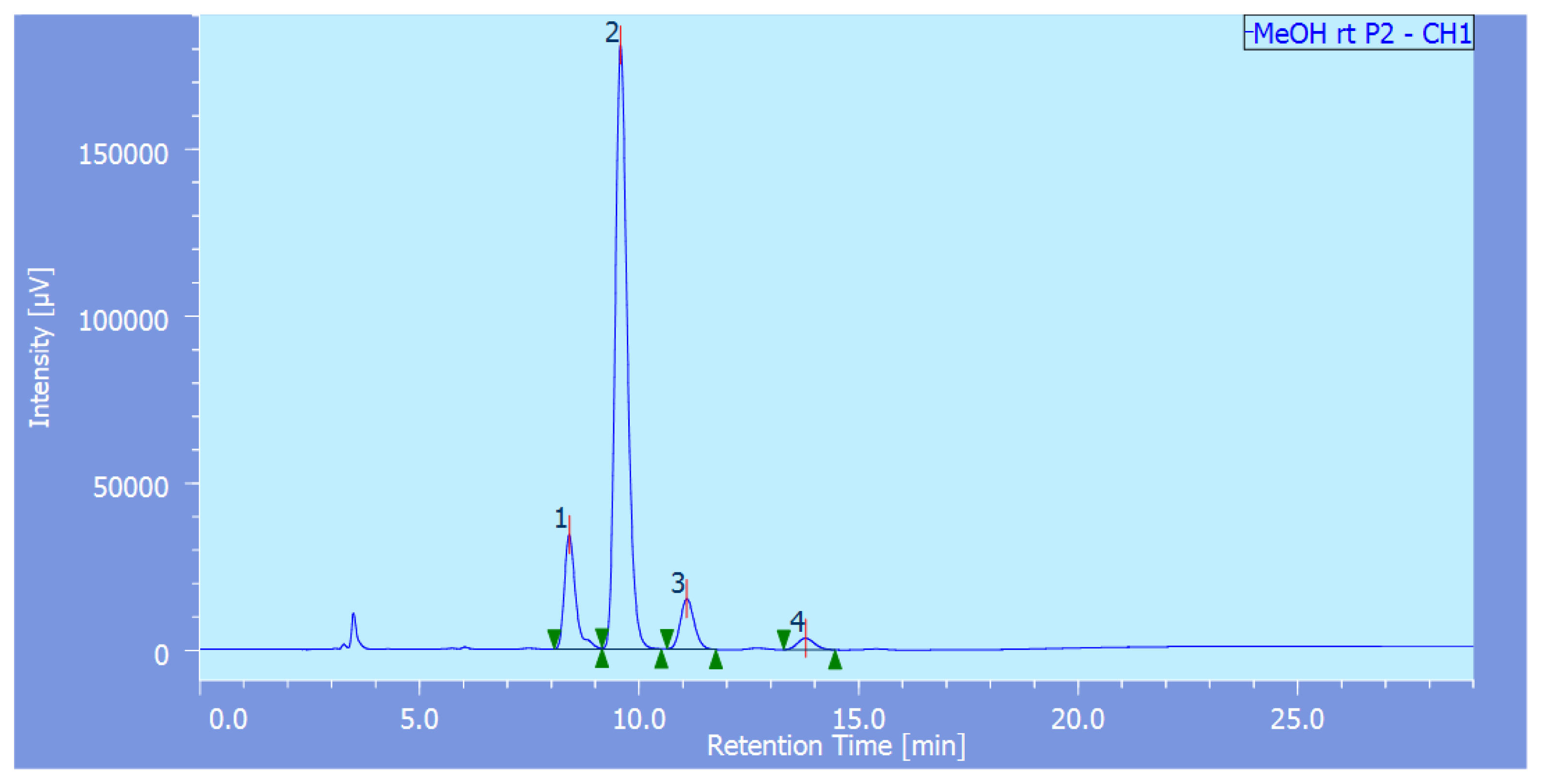

Supplement: Figure S52 — HPLC chromatogram of asymmetric compound, 13 Table 4, entry 3 95% ee [file tjc-48-04-512s52.tif]

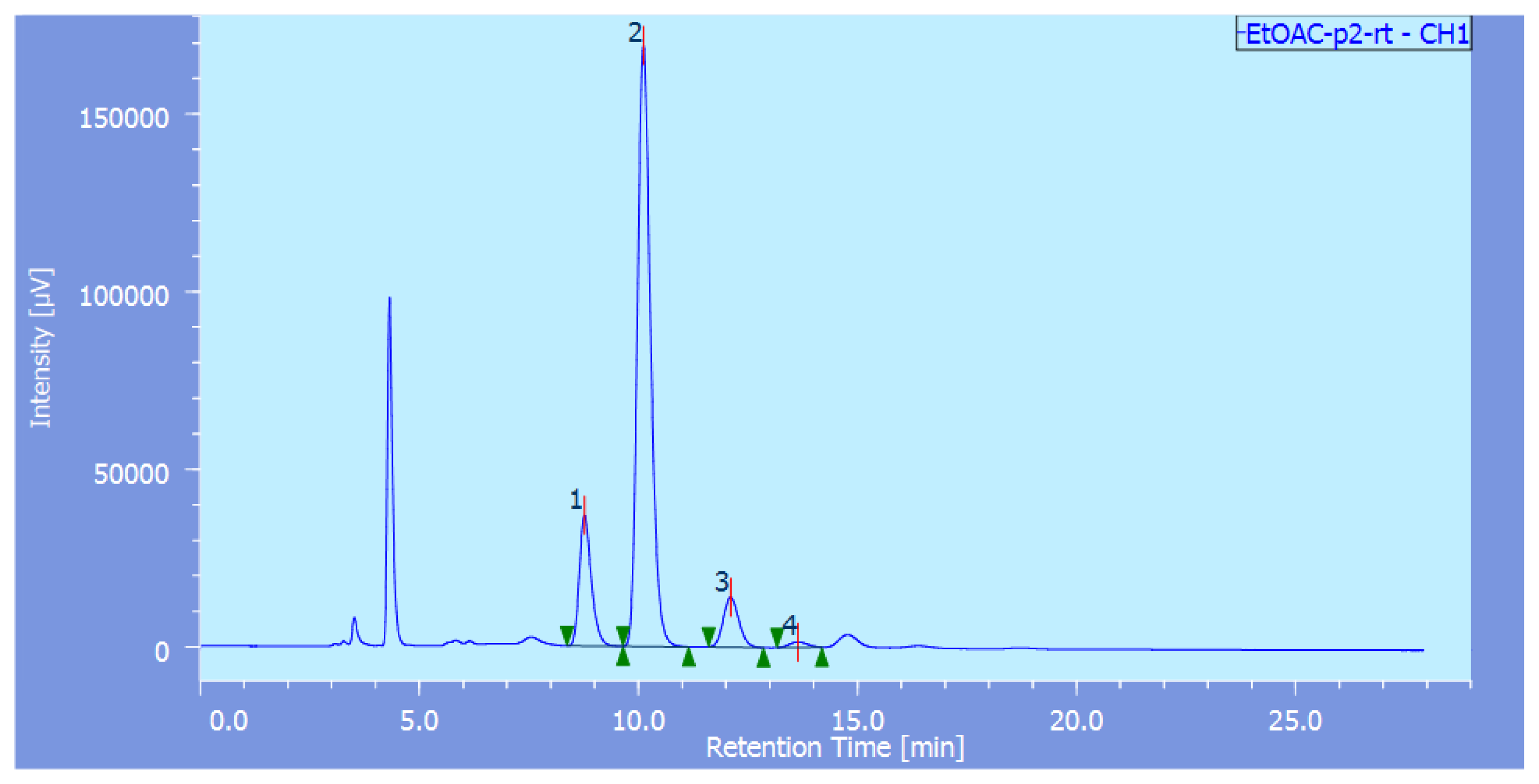

Supplement: Figure S53 — HPLC chromatogram of asymmetric compound, 13 Table 4, entry 4 97% ee [file tjc-48-04-512s53.tif]

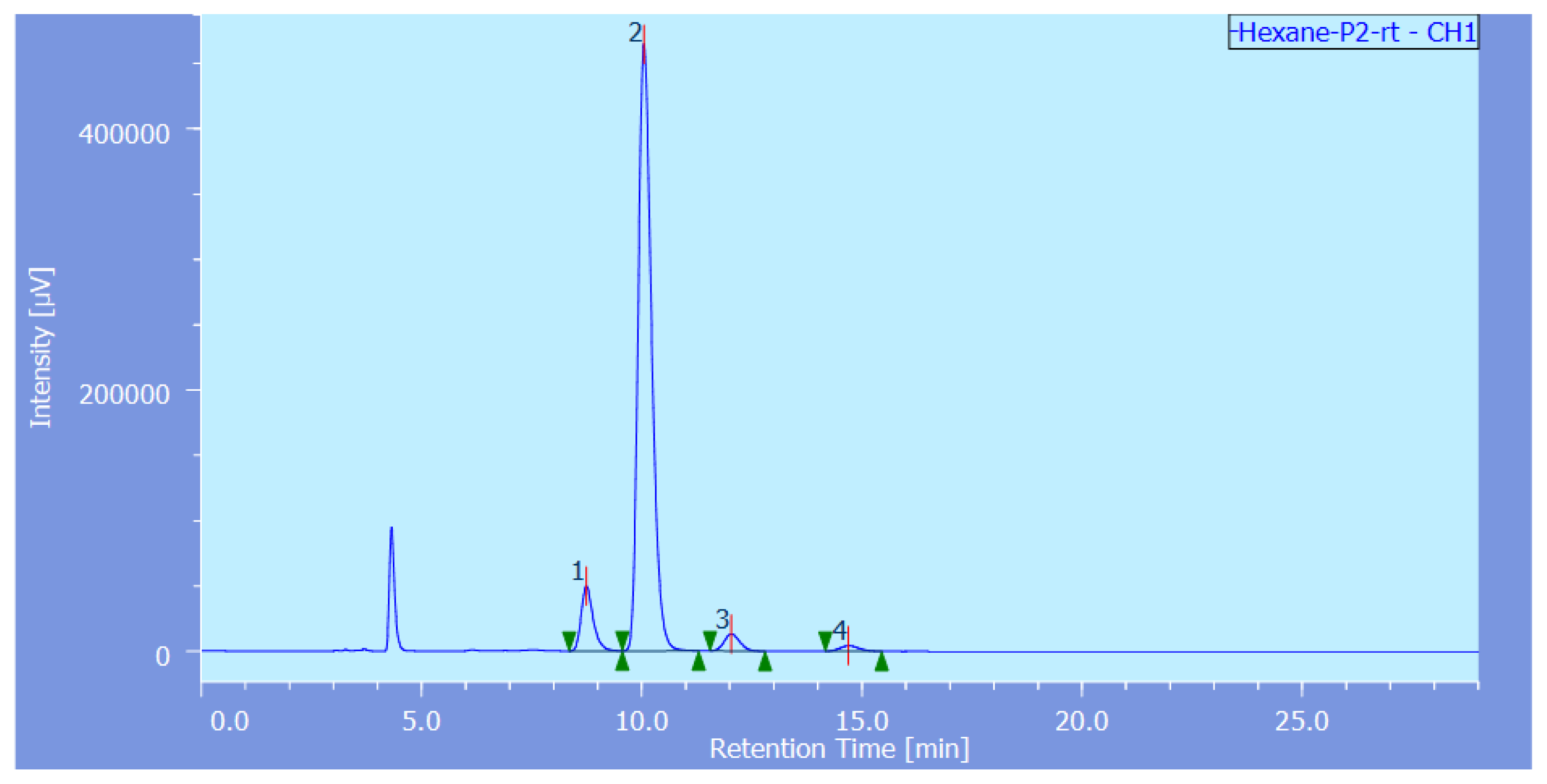

Supplement: Figure S54 — HPLC chromatogram of asymmetric compound, 13 Table 4, entry 5 97% ee [file tjc-48-04-512s54.tif]

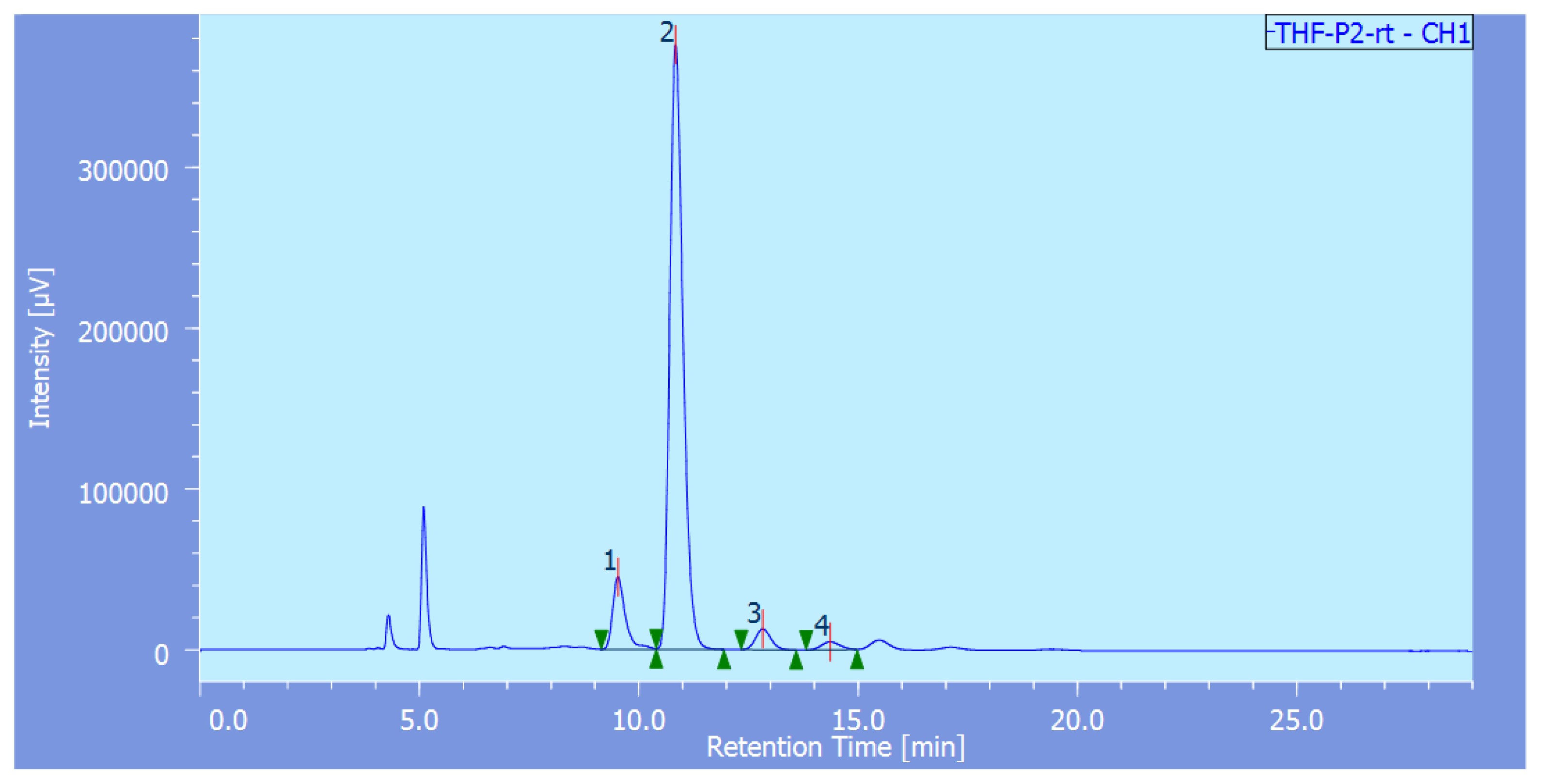

Supplement: Figure S55 — HPLC chromatogram of asymmetric compound, 13 Table 4, entry 6 96% ee [file tjc-48-04-512s55.tif]

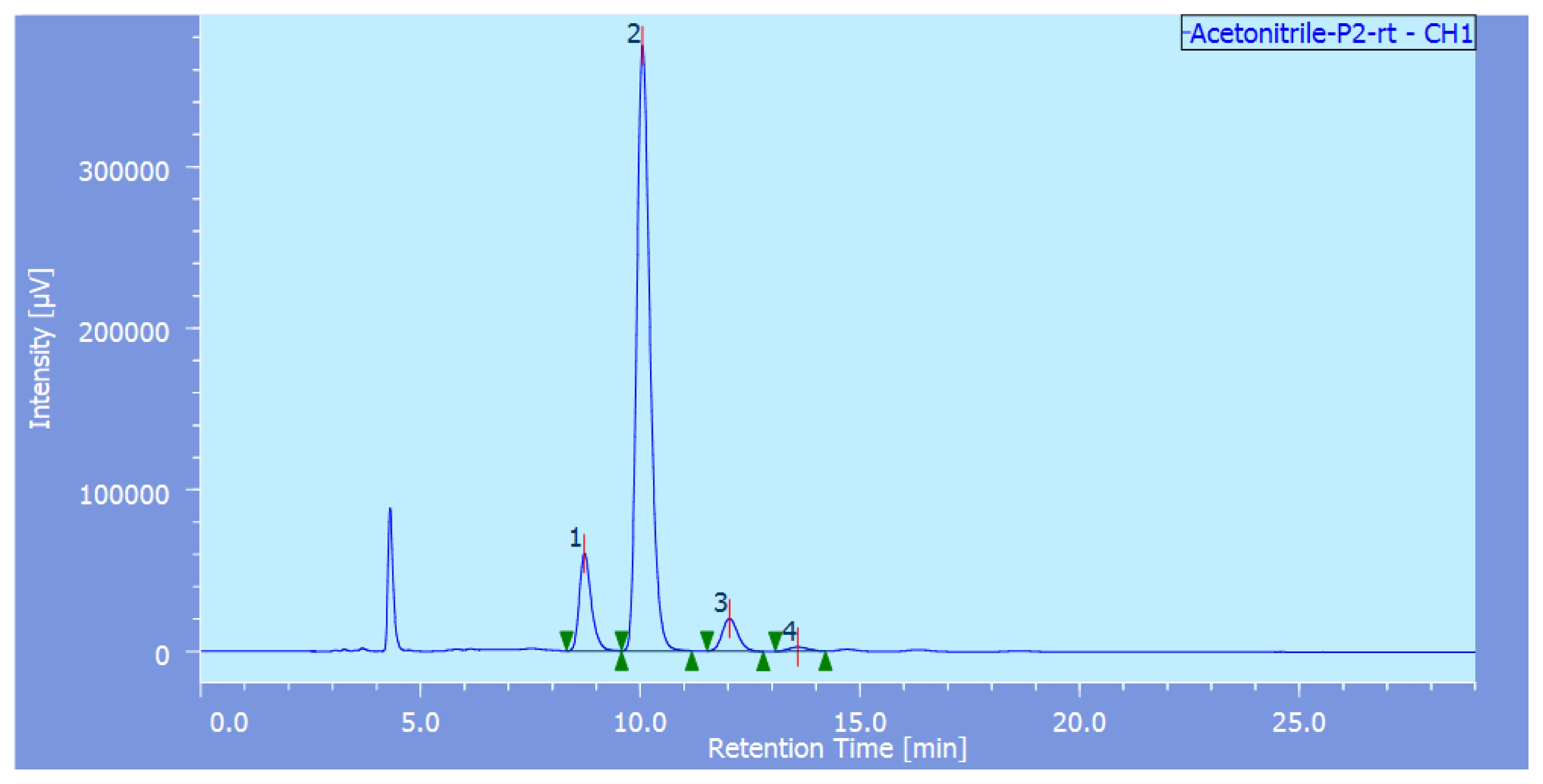

Supplement: Figure S56 — HPLC chromatogram of asymmetric compound, 13 Table 4, entry 7 98% ee [file tjc-48-04-512s56.tif]

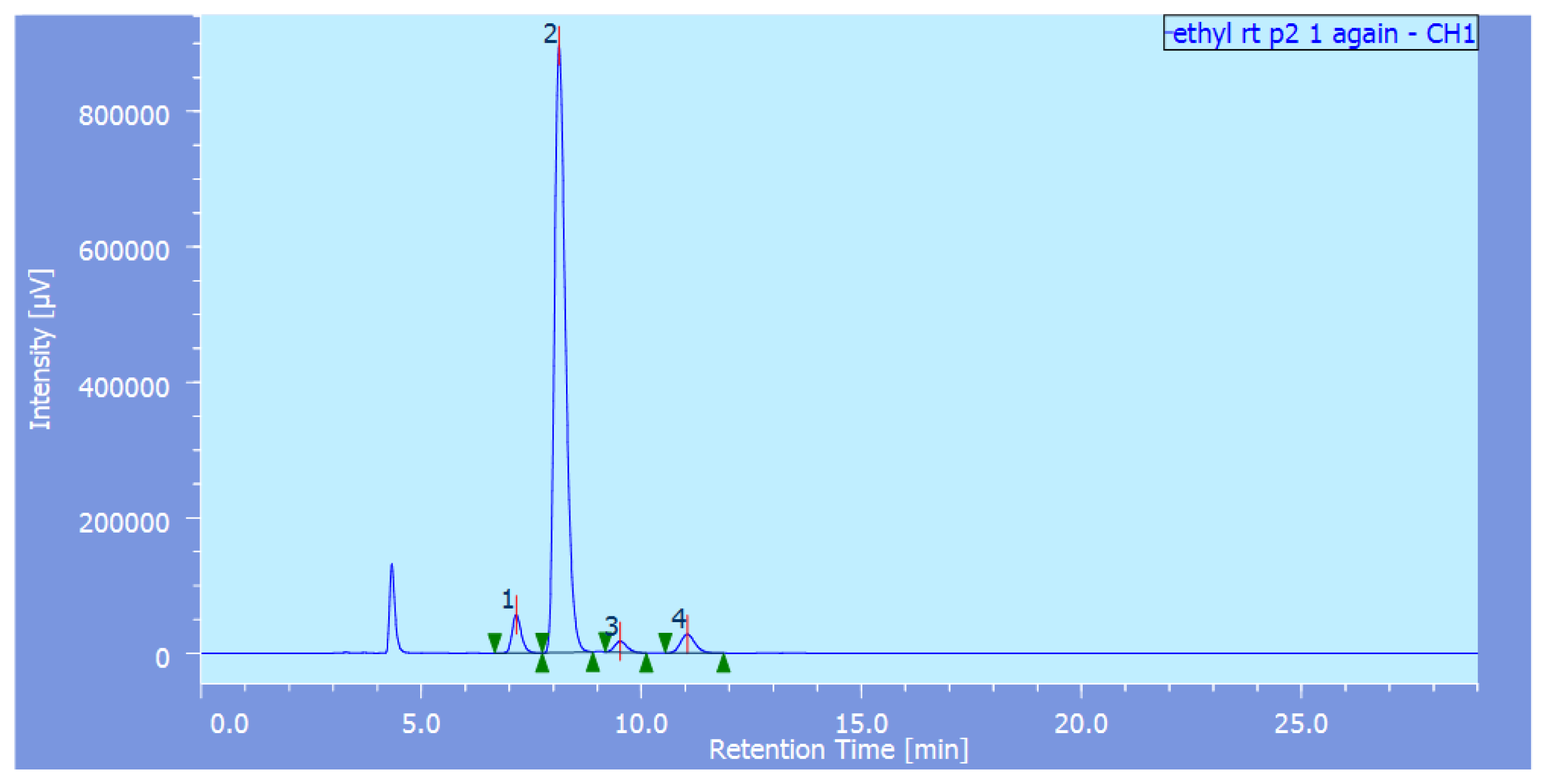

Supplement: Figure S57 — HPLC chromatogram of asymmetric compound, 18 Table 5, entry 1 92% ee [file tjc-48-04-512s57.tif]

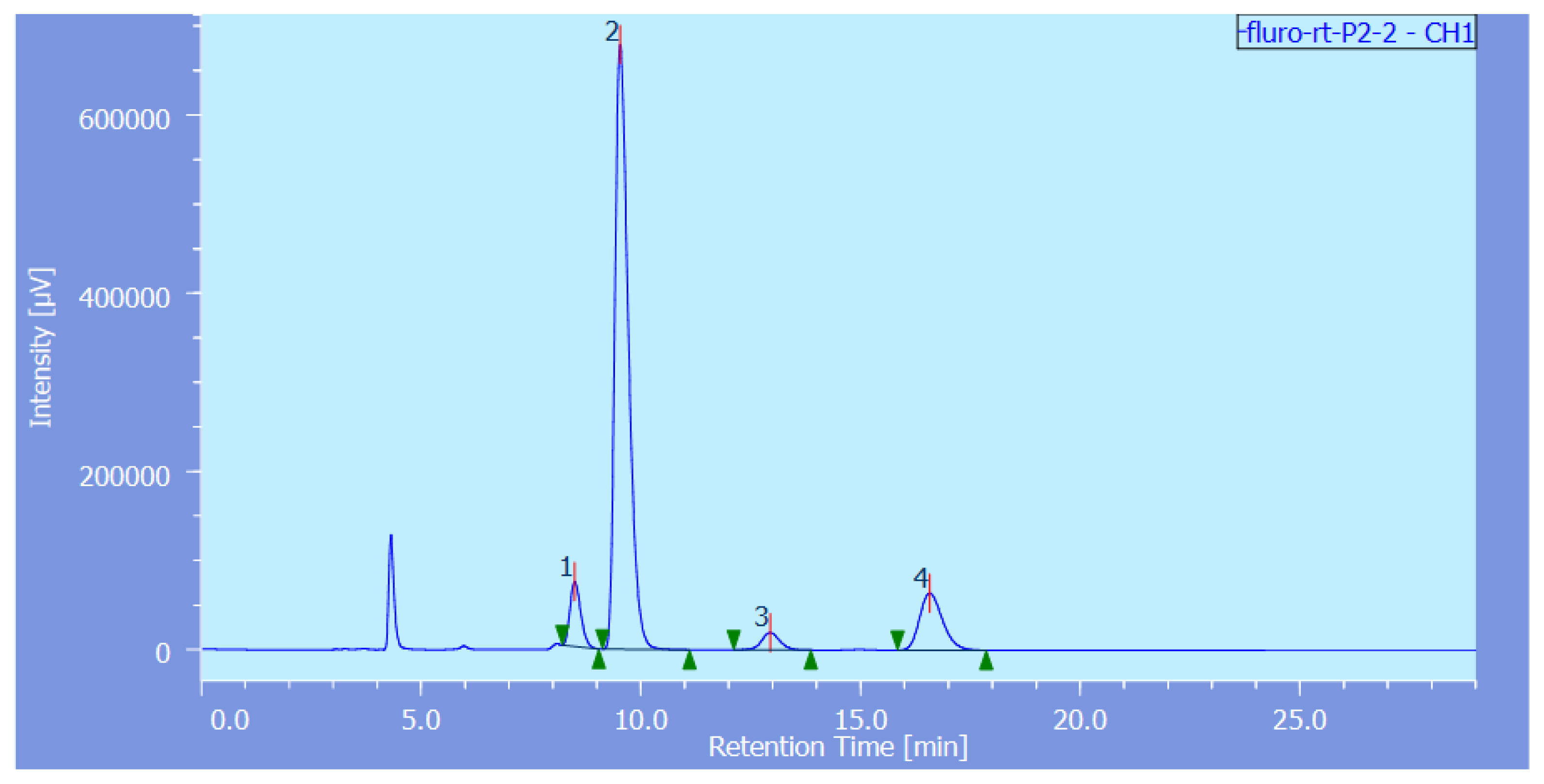

Supplement: Figure S58 — HPLC chromatogram of asymmetric compound, 19 Table 5, entry 2 73% ee [file tjc-48-04-512s58.tif]

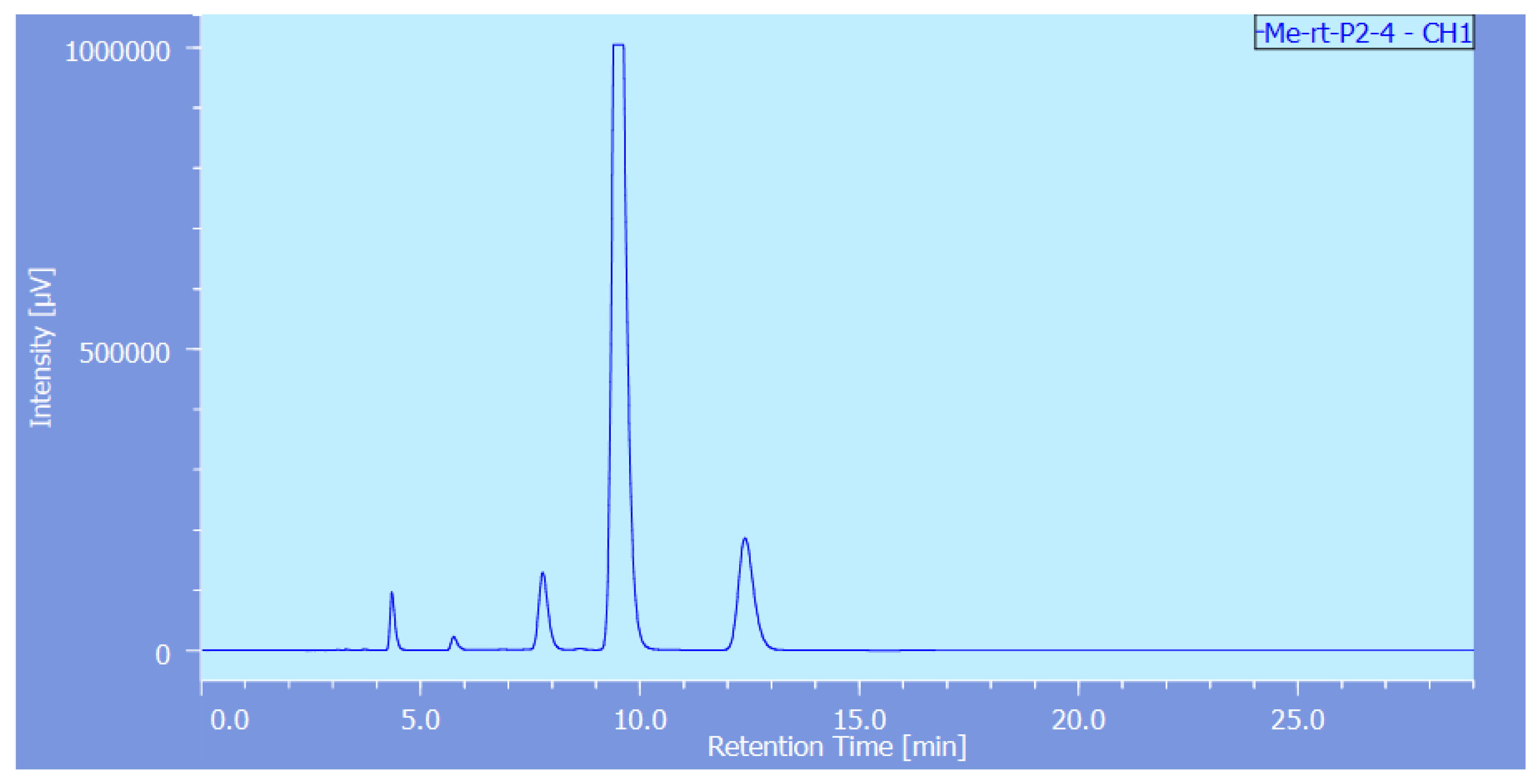

Supplement: Figure S59 — HPLC chromatogram of asymmetric compound, 20 Table 5, entry 3 >99% ee [file tjc-48-04-512s59.tif]

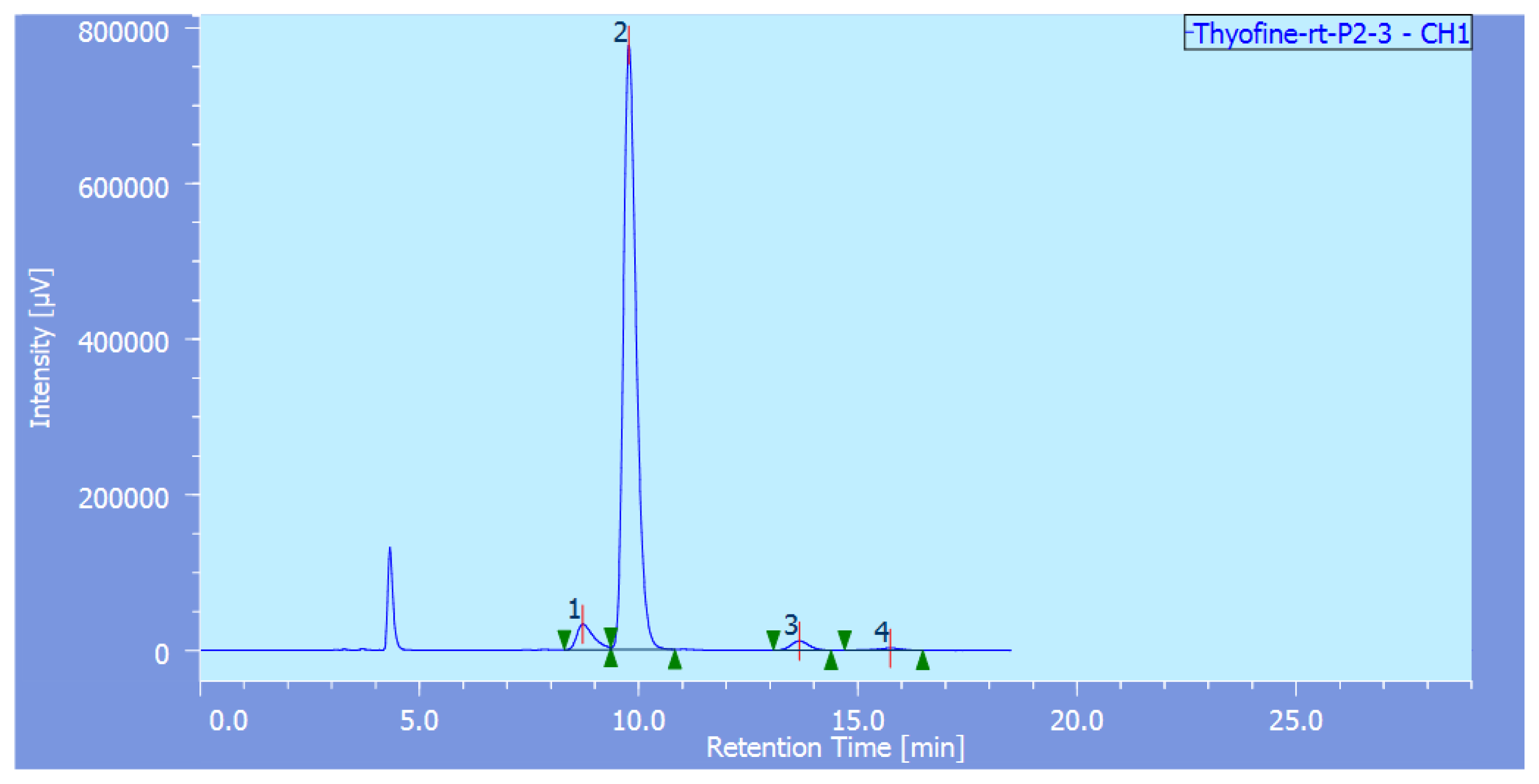

Supplement: Figure S60 — HPLC chromatogram of asymmetric compound, 21 Table 5, entry 4 99% ee [file tjc-48-04-512s60.tif]

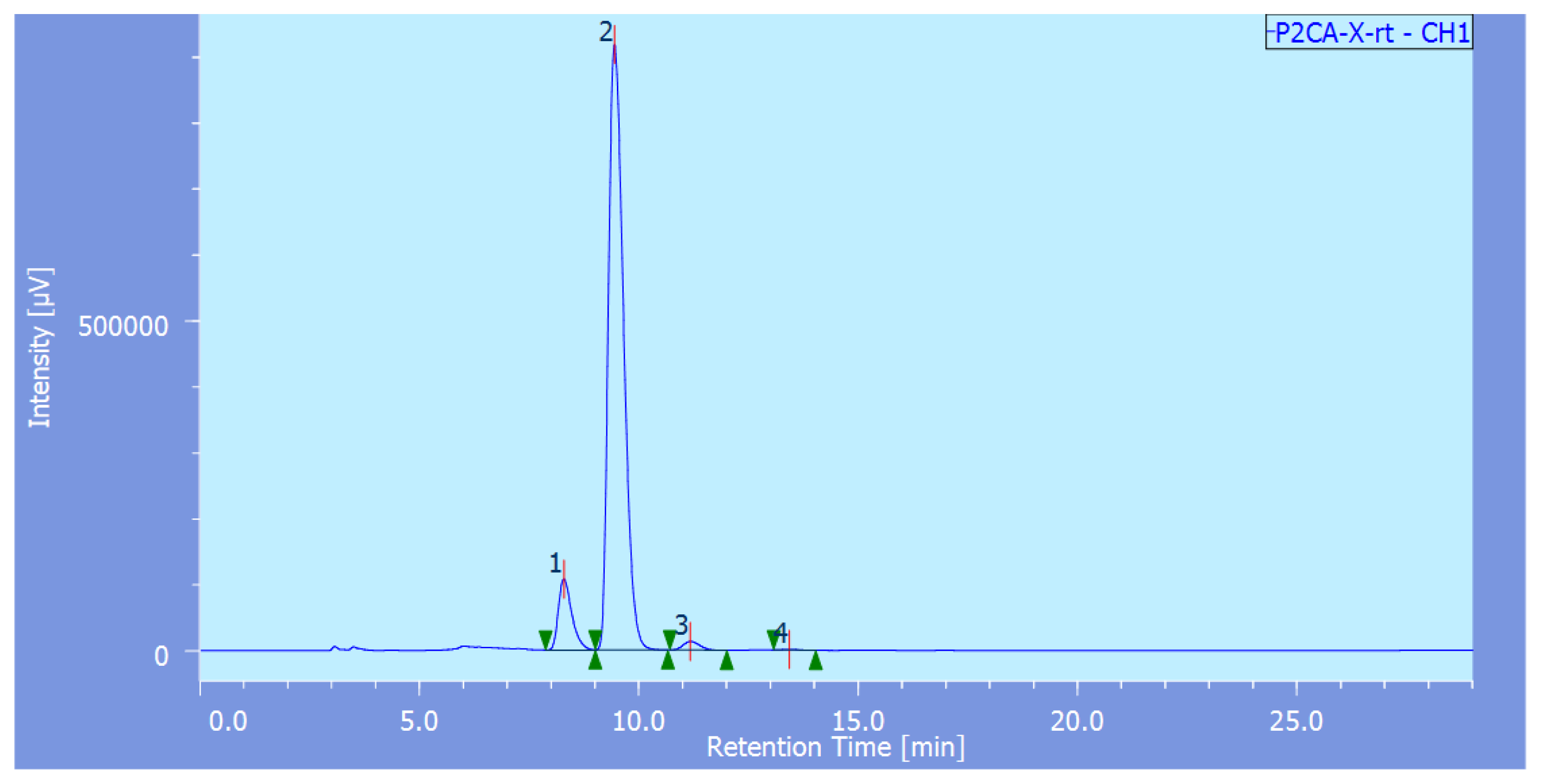

Supplement: Figure S61 — HPLC chromatogram of asymmetric compound, 13 Table 6, entry 1, fresh >99% ee [file tjc-48-04-512s61.tif]

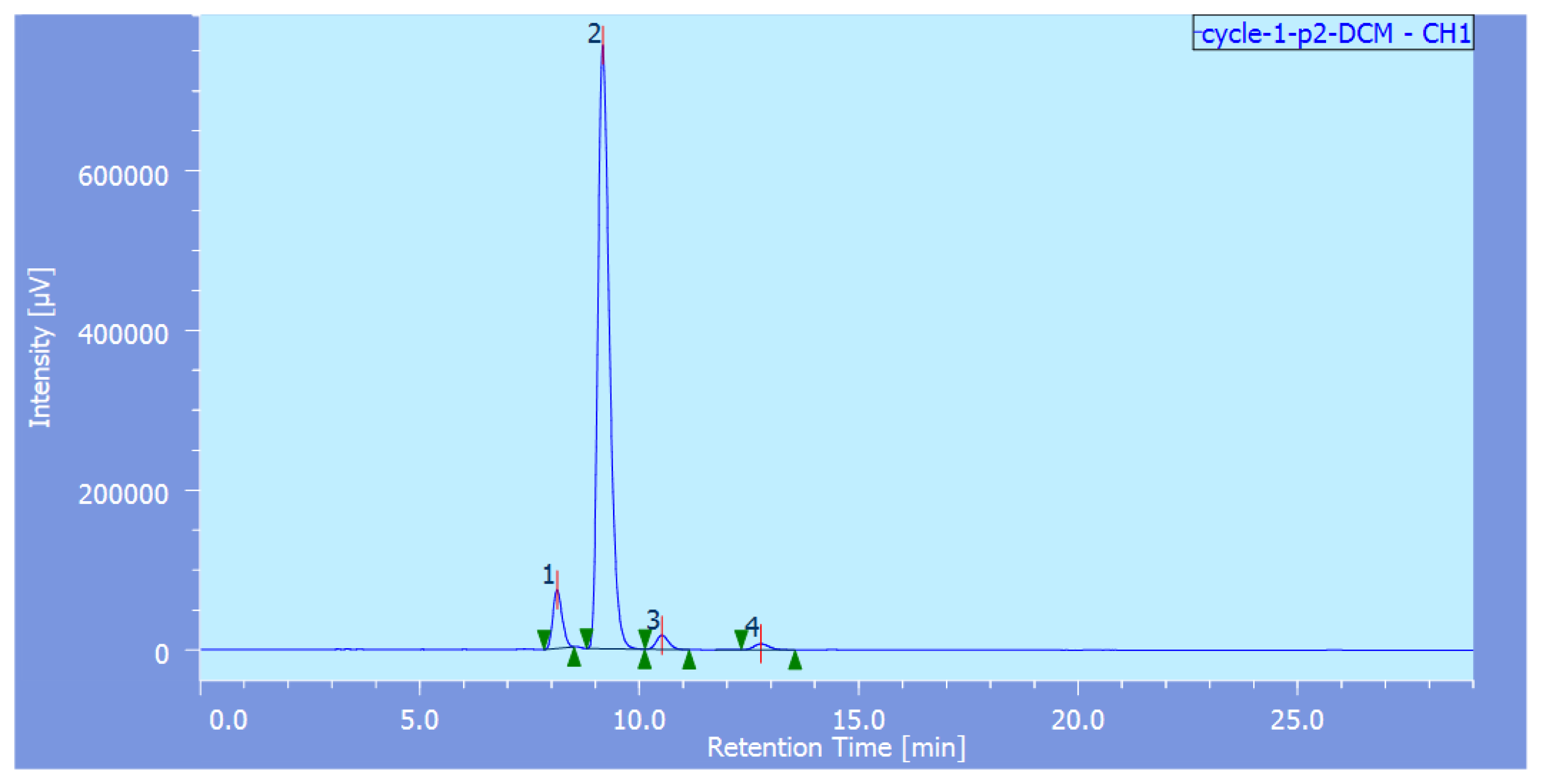

Supplement: Figure S62 — HPLC chromatogram of asymmetric compound, 13 Table 6, entry 2, cycle 1 97% ee [file tjc-48-04-512s62.tif]

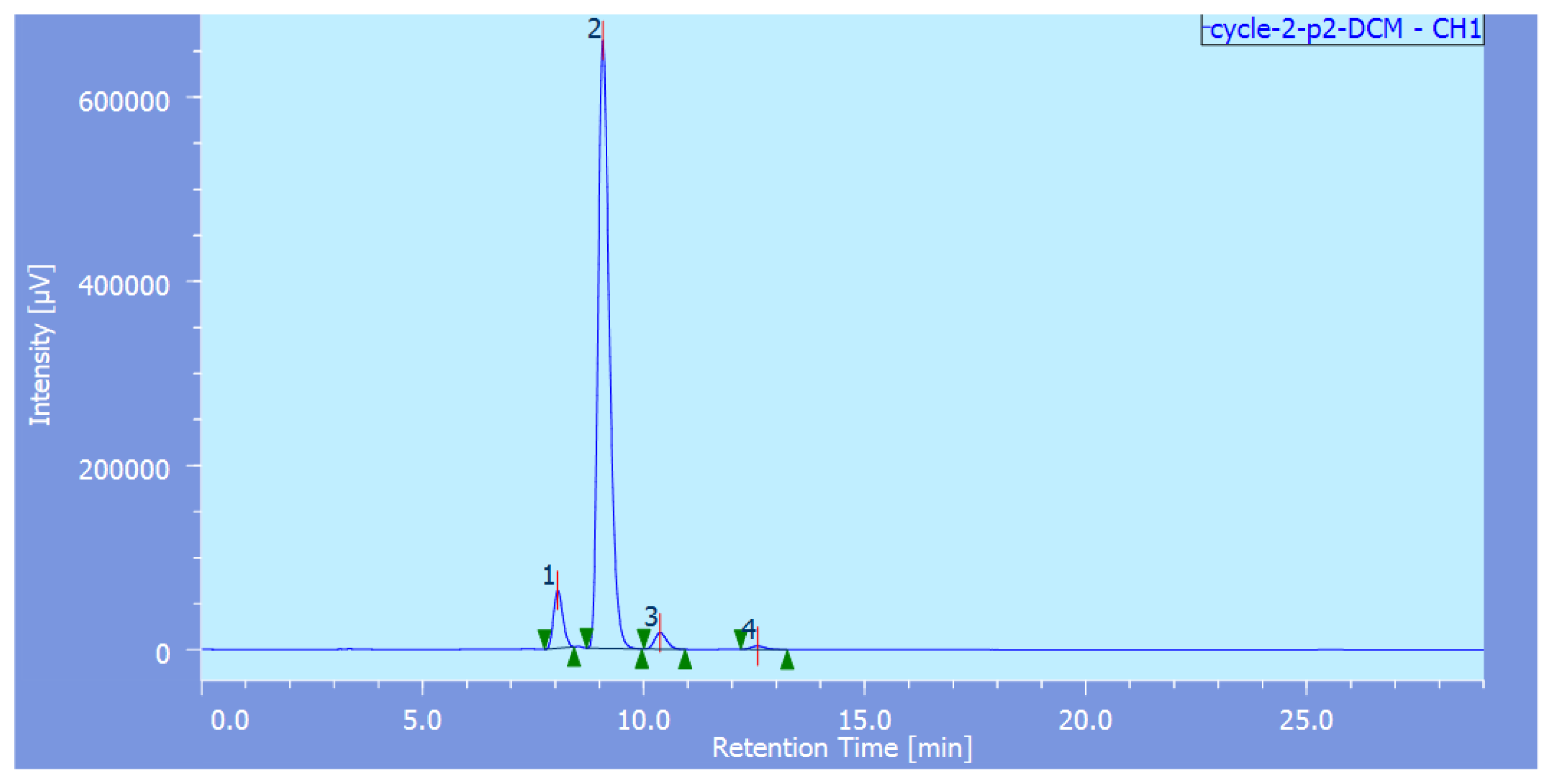

Supplement: Figure S63 — HPLC chromatogram of asymmetric compound, 13 Table 6, entry 3, cycle 2 99% ee [file tjc-48-04-512s63.tif]

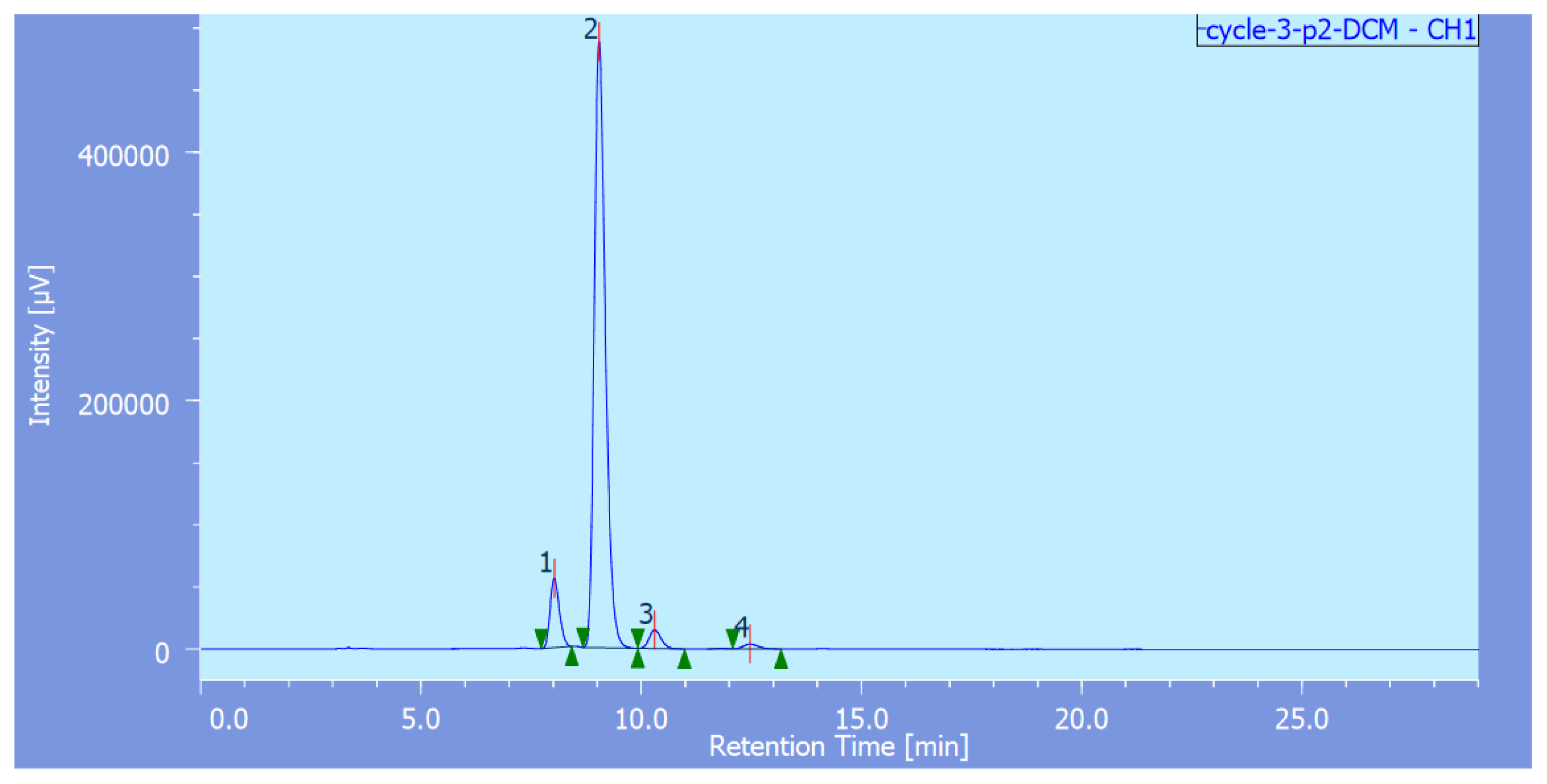

Supplement: Figure S64 — HPLC chromatogram of asymmetric compound, 13 Table 6, entry 4, cycle 3 98% ee [file tjc-48-04-512s64.tif]

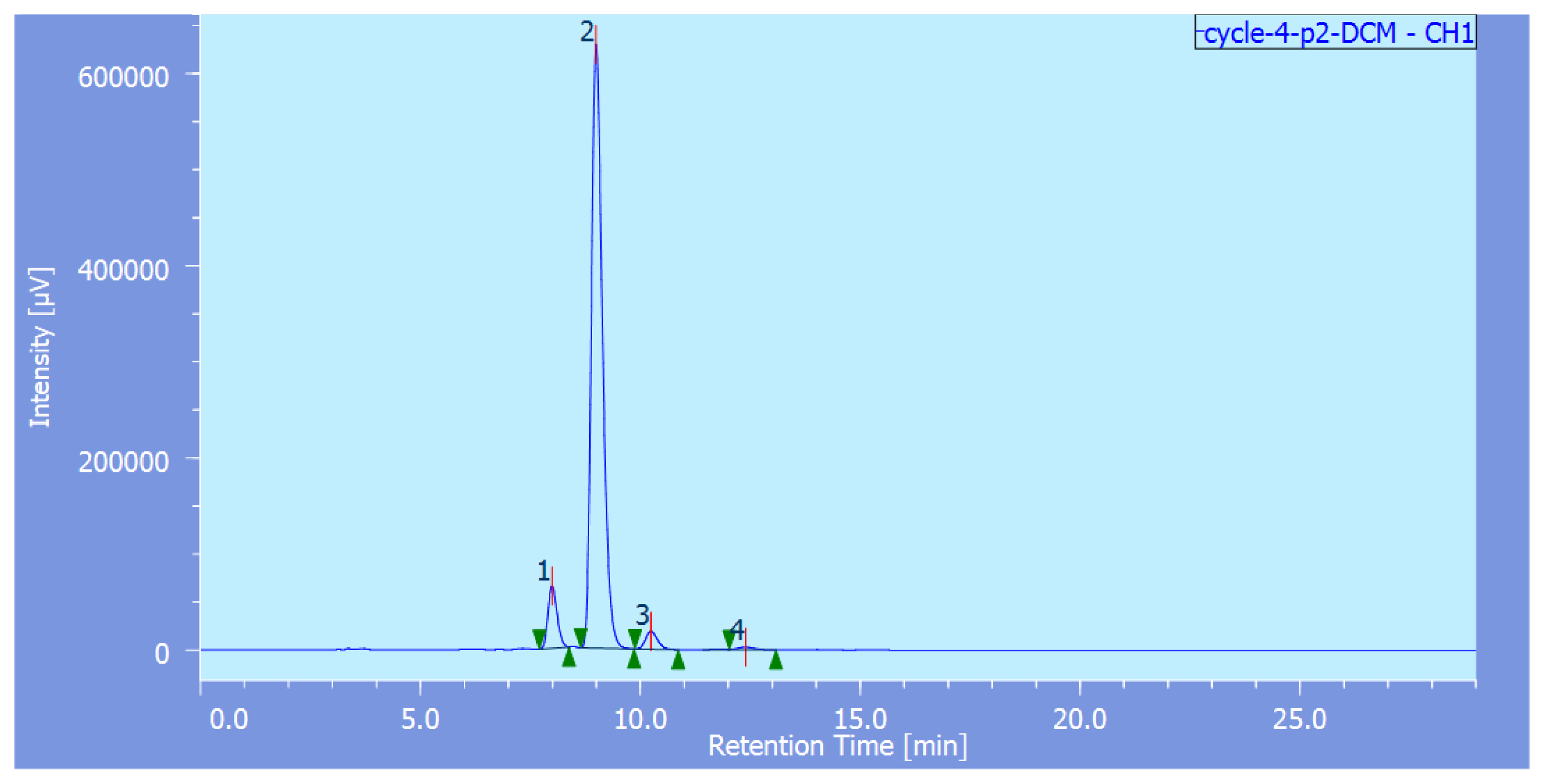

Supplement: Figure S65 — HPLC chromatogram of asymmetric compound, 13 Table 6, entry 5, cycle 4 99% ee [file tjc-48-04-512s65.tif]
